# Respiratory Viruses in Patients With Hematological Malignancy in Boreal Autumn/Winter 2023–2024: EPICOVIDEHA‐EPIFLUEHA Report

**DOI:** 10.1002/ajh.27565

**Published:** 2024-12-23

**Authors:** Jon Salmanton‐García, Francesco Marchesi, Milan Navrátil, Klára Piukovics, Maria Ilaria del Principe, Marianna Criscuolo, Yavuz M. Bilgin, Nicola S. Fracchiolla, Antonio Vena, Alessandra Romano, Iker Falces‐Romero, Nicola Sgherza, Inmaculada Heras‐Fernando, Monika M. Biernat, Verena Petzer, Pavel Žák, Barbora Weinbergerová, Michail Samarkos, Nurettin Erben, Jens van Praet, Alberto López‐García, Jorge Labrador, Tobias Lahmer, Ľuboš Drgoňa, Maria Merelli, Annarosa Cuccaro, Sonia Martín‐Pérez, Julio Dávila‐Valls, Francesca Farina, Chiara Cattaneo, László Imre Pinczés, Ferenc Magyari, Ildefonso Espigado, Caterina Buquicchio, Donald C. Vinh, Igor Stoma, Martin Čerňan, Lucia Prezioso, Mario Virgilio Papa, Gaëtan Plantefeve, Reham Abdelaziz Khedr, Josip Batinić, Gabriele Magliano, Simge Erdem, Sofya Khostelidi, Natasha Čolović, Davide Nappi, Patricia García‐Ramírez, Jakub Góra, Marta Callejas‐Charavia, Jędrzej Tłusty, Martijn Bakker, Elwira Wojtyniak, Darko Antić, Agnieszka Magdziak, Michelina Dargenio, Larisa Idrizović, Nikola Pantić, Zlate Stojanoski, Noha Eisa, Vladimir Otašević, Monia Marchetti, Erica Mackenzie, Carolina Garcia‐Vidal, Avinash Aujayeb, Ahlam Almasari, Carolina Miranda‐Castillo, Eleni Gavriilaki, Nicola Coppola, Alessandro Busca, Tatjana Adžić‐Vukičević, Martin Schönlein, Ditte Stampe Hersby, Stefanie K. Gräfe, Andreas Glenthøj, Tommaso Francesco Aiello, Milche Cvetanoski, Mirjana Mitrović, Claudio Cerchione, Romane Prin, Gina Varricchio, Elena Arellano, Raúl Córdoba, Jiří Mayer, Benjamín Víšek, Dominik Wolf, Amalia N. Anastasopoulou, Mario Delia, Pellegrino Musto, Dario Leotta, Martina Bavastro, Alessandro Limongelli, Mariarita Sciumè, Lukas van den Ven, Luana Fianchi, Sara Caterina Brunetti, Joanna Drozd‐Sokołowska, Anna Dąbrowska‐Iwanicka, Oliver A. Cornely, Livio Pagano

**Affiliations:** ^1^ Faculty of Medicine, University of Cologne and University Hospital Cologne, Institute of Translational Research, Excellence Cluster on Cellular Stress Responses in Aging‐Associated Diseases (CECAD) Cologne Germany; ^2^ Faculty of Medicine, University of Cologne, University Hospital Cologne, Department I of Internal Medicine, Center for Integrated Oncology Aachen Bonn Cologne Duesseldorf (CIO ABCD) and Excellence Center for Medical Mycology (ECMM) Cologne Germany; ^3^ German Centre for Infection Research (DZIF), partner Site Bonn‐Cologne Cologne Germany; ^4^ Hematology and Stem Cell Transplant Unit IRCCS Regina Elena National Cancer Institute Rome Italy; ^5^ Department of Haematooncology University Hospital Ostrava Ostrava Czech Republic; ^6^ Department of Haematooncology, Faculty of Medicine University of Ostrava Ostrava Czech Republic; ^7^ Department of Hematology, South Division of Internal Medicine Clinic, Albert Szent‐Györgyi Health Center University of Szeged Szeged Hungary; ^8^ Hematology, Department of Biomedicine and Prevention University of Rome Tor Vergata Rome Italy; ^9^ Hematology Unit, Fondazione Policlinico Universitario Agostino Gemelli – IRCCS Rome Italy; ^10^ Department of Internal Medicine, ADRZ Goes Netherlands; ^11^ Hematology Unit, Fondazione IRCCS Ca' Granda Ospedale Maggiore Policlinico Milan Italy; ^12^ Department of Health Sciences (DISSAL) University of Genoa Genoa Italy; ^13^ UO Clinica Malattie Infettive, IRCCS Ospedale Policlinico San Martino Genoa Italy; ^14^ AOU Policlinico Rodolico San Marco Catania Italy; ^15^ Microbiology and Parasitology Department University Hospital La Paz Madrid Spain; ^16^ CIBERINFEC, Instituto de Salud Carlos III Madrid Spain; ^17^ Hematology and Stem Cell Transplantation Unit, AOUC Policlinico Bari Italy; ^18^ Department of Hematology and Oncology Hospital Morales Messeguer Murcia Spain; ^19^ Department of Hematology, Blood Neoplasms, and Bone Marrow Transplantation Wroclaw Medical University Wroclaw Poland; ^20^ Department of Hematology and Oncology, Comprehensive Cancer Center Innsbruck (CCCI) Medical University of Innsbruck (MUI) Innsbruck Austria; ^21^ University Hospital Hradec Králové Hradec Králové Czech Republic; ^22^ Department of Internal Medicine, Hematology and Oncology Masaryk University and University Hospital Brno Brno Czech Republic; ^23^ Laikon Hospital, Medical School National and Kapodistrian University of Athens Athens Greece; ^24^ Department of Infectious Diseases and Clinical Microbiology, Faculty of Medicine Eskisehir Osmangazi University Eskisehir Turkey; ^25^ Department of Nephrology and Infectious Diseases AZ Sint‐Jan Brugge‐Oostende AV Brugge Belgium; ^26^ Fundacion Jimenez Diaz University Hospital Health Research Institute IIS‐FJD Madrid Spain; ^27^ Department of Hematology, Research Unit, Hospital Universitario de Burgos Burgos Spain; ^28^ Medizinische Klinik II, Klinikum Rechts der Isar, TU München Munich Germany; ^29^ Comenius University and National Cancer Institute Bratislava Slovakia; ^30^ Azienda Sanitaria Universitaria del Friuli Centrale Udine Italy; ^31^ Hematology Unit, Center for Translational Medicine, AziendaUSL Toscana NordOvest Livorno Italy; ^32^ National Cancer Institute, Fondazione ‘G. Pascale’, IRCCS, Hematology‐Oncology and Stem Cell Transplantation Unit Naples Italy; ^33^ Hospital Nuestra Señora de Sonsoles Ávila Spain; ^34^ IRCCS Ospedale San Raffaele Milan Italy; ^35^ Hematology Unit, ASST‐Spedali Civili Brescia Italy; ^36^ Division of Hematology, Department of Internal Medicine University of Debrecen Debrecen Hungary; ^37^ Department of Hematology University Hospital Virgen Macarena – University Hospital Virgen del Rocío, Instituto de Biomedicina de Sevilla (IBIS/CSIC), Universidad de Sevilla (Departamento de Medicina) Seville Spain; ^38^ Ematologia Con Trapianto, Ospedale Dimiccoli Barletta Barletta Italy; ^39^ McGill University Health Centre Montreal Canada; ^40^ Gomel State Medical University Gomel Belarus; ^41^ University Hospital Olomouc Olomouc Czech Republic; ^42^ Hematology and Bone Marrow Unit Hospital University of Parma Parma Italy; ^43^ Azienda Ospedaliera Sant'Anna e San Sebastiano Caserta Italy; ^44^ Head ICU and CRC, Centre Hospitalier Victor DUPOUY Argenteuil France; ^45^ Department of Pediatric Oncology, National Cancer Institute Cairo University Cairo Egypt; ^46^ Department of Pediatric Oncology Children's Cancer Hospital Cairo Egypt; ^47^ University Hospital Centre Zagreb Zagreb Croatia; ^48^ Croatian Cooperative Group for Hematological Diseases (CROHEM) Zagreb Croatia; ^49^ Faculty of Medicine University of Zagreb Zagreb Croatia; ^50^ ASST Grande Ospedale Metropolitano Niguarda Milan Italy; ^51^ Division of Hematology, Department of Internal Medicine, Faculty of Medicine Istanbul University Istanbul Turkey; ^52^ North‐Western State Medical University Named After Iliá Ilich Méchnikov Saint‐Petersburg Russia; ^53^ University Clinical Center of Serbia Belgrade Serbia; ^54^ Hematology Unit, Istituto Scientifico Romagnolo per lo Studio e la Cura Dei Tumori (IRST) IRCCS Meldola Italy; ^55^ Servicio de Hematología y Hemoterapia, Hospital Universitario Príncipe de Asturias Alcalá de Henares Spain; ^56^ Medical University of Warsaw Warszawa Poland; ^57^ University Medical Center Groningen Groningen The Netherlands; ^58^ Department of Clinical Microbiology, Maria Skłodowska‐Curie National Research Institute of Oncology Warszawa Poland; ^59^ Ospedale Vito Fazzi Lecce Italy; ^60^ University Clinic of Hematology Skopje North Macedonia; ^61^ Faculty of Medicine Mansoura University Mansoura Egypt; ^62^ King Faisal Specialist Hospital Jeddah Saudi Arabia; ^63^ Hematology and Transplant Unit, Azienda Ospedaliera SS Antonio e Biagio e Cesare Arrigo Alessandria Italy; ^64^ University of Kansas Medical Center Kansas City Missouri USA; ^65^ Department of Infectious Diseases, Hospital Clinic de Barcelona University of Barcelona, IDIBAPS Barcelona Spain; ^66^ Northumbria Healthcare Newcastle UK; ^67^ Hospital Rey Juan Carlos Móstoles Spain; ^68^ General Hospital of Thessaloniki “George Papanikolaou” Thessaloniki Greece; ^69^ Department of Mental Health and Public Medicine, Universitry of Campania Naples Italy; ^70^ Stem Cell Transplant Center, AOU Cittá della Salute e della Scienza Turin Italy; ^71^ Department of Oncology, Hematology and Bone Marrow Transplantation With Section of Pneumology University Medical Center Hamburg‐Eppendorf Hamburg Germany; ^72^ Department of Hematology Copenhagen University Hospital – Rigshospitalet Copenhagen Denmark; ^73^ University Medical Center Hamburg‐Eppendorf Hamburg Germany; ^74^ CRA From CRC Centre Hospitalier Victor DUPOUY Argenteuil France; ^75^ Maria Skłodowska‐Curie Institute of Oncology Warszawa Poland; ^76^ Faculty of Medicine University of Cologne and University Hospital Cologne, Clinical Trials Centre Cologne (ZKS Köln) Cologne Germany; ^77^ Hematology Unit Università Cattolica del Sacro Cuore Rome Italy

**Keywords:** antiviral therapy, community‐acquired respiratory viral infection, hematological malignancy, secondary infection, vaccine coverage

## Abstract

Community‐acquired respiratory viral infections (CARV) significantly impact patients with hematological malignancies (HM), leading to high morbidity and mortality. However, large‐scale, real‐world data on CARV in these patients is limited. This study analyzed data from the EPICOVIDEHA‐EPIFLUEHA registry, focusing on patients with HM diagnosed with CARV during the 2023–2024 autumn–winter season. The study assessed epidemiology, clinical characteristics, risk factors, and outcomes. The study examined 1312 patients with HM diagnosed with CARV during the 2023–2024 autumn–winter season. Of these, 59.5% required hospitalization, with 13.5% needing ICU admission. The overall mortality rate was 10.6%, varying by virus: parainfluenza (21.3%), influenza (8.8%), metapneumovirus (7.1%), RSV (5.9%), or SARS‐CoV‐2 (5.0%). Poor outcomes were significantly associated with smoking history, severe lymphopenia, secondary bacterial infections, and ICU admission. This study highlights the severe risk CARV poses to patients with HM, especially those undergoing active treatment. The high rates of hospitalization and mortality stress the need for better prevention, early diagnosis, and targeted therapies. Given the severe outcomes with certain viruses like parainfluenza, tailored strategies are crucial to improving patient outcomes in future CARV seasons.

## Graphical abstract

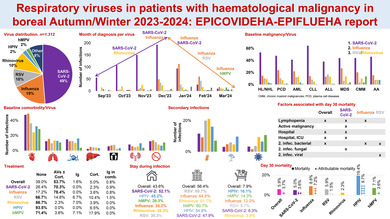

## 1 Introduction

Community‐acquired respiratory viral infections (CARV) are a significant concern for patients with hematological malignancy (HM), whether they have undergone hematopoietic stem cell transplantation (HSCT) or not [1, 2, 3]. CARV can severely compromise the effectiveness of anticancer treatments, making them one of the most challenging complications in HM patients [2, 4]. In recent years, there has been a growing understanding of the importance and the management of CARV in HM. A rising interest is reflected in the increasing body of scientific literature exploring the role and impact of CARV, including consensus guidelines [5, 6, 7]. However, large‐scale data from cooperative registries, which are crucial for developing real‐world, evidence‐based strategies, remain scarce. Such data could be invaluable to the hematology community, providing critical insights into the epidemiology, risk factors, and clinical outcomes of CARV in HM patients. With this knowledge, clinicians could more effectively mitigate the adverse effects of these infections on the overall treatment process.

The EPICOVIDEHA registry [8], established in 2021, has been instrumental in addressing this gap by collecting extensive data on severe acute respiratory syndrome coronavirus 2 (SARS‐CoV‐2) infections in HM patients. The insights from EPICOVIDEHA registry have led to numerous scientific publications that have been vital in guiding clinicians throughout the coronavirus disease 2019 (COVID‐19) pandemic, helping to develop preventive strategies to protect HM patients [9, 10, 11, 12, 13, 14, 15, 16, 17, 18, 19, 20, 21, 22, 23, 24, 25, 26, 27, 28, 29]. Building on the success of the SARS‐CoV‐2‐dedicated data collection, the EPICOVIDEHA registry expanded its scope in 2023 to include other CARV and was renamed as EPICOVIDEHA‐EPIFLUEHA [30]. This expansion aimed to provide a more comprehensive understanding of the epidemiology, risk factors, and clinical outcomes associated with CARV in HM patients.

In this manuscript, we present and analyze CARV cases in HM patients registered during the autumn–winter seasons of 2023–2024. Our goal is to contribute valuable data that can guide future strategies in the ongoing effort to improve care for HM patients facing the threat of respiratory viral infections.

## 2 Methods

### 2.1 Patients

A total of 61 institutions from 24 countries (Figure S1) contributed data on CARV cases diagnosed between September 1, 2023, and March 31, 2024, in HM patients to the EPICOVIDEHA‐EPIFLUEHA registry [8, 30], accessible at www.clinicalsurveys.net (EFS, TIVIAN, Cologne, Germany). EPICOVIDEHA‐EPIFLUEHA, identified by National Clinical Trials Identifier NCT04733729, is an international, web‐based registry focused on HM adult patients who contract CARV. It was established by the European Hematology Association Specialized Working Group (EHA‐SWG) Infections in Hematology.

Patients were eligible if they had an active HM within the last 5 years immediately before the CARV diagnosis, were 18 years or older, had a laboratory‐confirmed CARV, and received their diagnosis between September 1, 2023, and March 31, 2024. The period from September 2023 to March 2024 was chosen as it aligns with the peak season for CARV infections in the Northern hemisphere [31], enabling a representative analysis in terms of comparability and impact on healthcare systems. Excluded were patients with solid tumors, nonmalignant hematological disorders, age < 18 years, those off‐therapy or cured for more than 5 years before their respiratory viral infection, or those diagnosed solely via imaging. No restrictions were imposed concerning the type of pathogenic virus.

For each patient, data collected included baseline conditions prior to the CARV, such as age, biological sex, and HM status at diagnosis. Information on HM management (status and type of last treatment before infection), CARV diagnosis and symptomatology, prophylaxis and treatments (including vaccines against influenza, respiratory syncytial virus (RSV), and SARS‐CoV‐2 administered within the year preceding the onset of infection), stay during infection, and outcomes (mortality and last follow‐up date) were recorded. Patients who received an influenza, RSV, or SARS‐CoV‐2 vaccination more than 1 year prior to their CARV infection diagnosis were classified as “non‐vaccinated” for the purposes of this analysis. The status of HM at infection onset was classified as active (onset, refractory, and stable disease) or controlled (complete response). The severity of respiratory viral infection episodes was categorized as asymptomatic, mild, severe, or critical, as in previous EPICOVIDEHA‐EPIFLUEHA publications [22, 23, 25]. To ensure data accuracy and completeness, a validation process was conducted by experts in hematology and infectious diseases. Contributors were contacted to resolve any pending queries, which helped maintain the integrity and reliability of the registry data. Missing data for variables included in regression analyses led to exclusion from the analysis.

### 2.2 Objectives

The primary objective was to examine the epidemiology and outcomes of HM patients affected by respiratory viruses during the specified period. Secondary objectives included determining the relative frequency of disease severity, intensive care unit (ICU) admissions, overall case‐fatality rate, the impact of cancer treatment phases on outcomes, the effect of vaccine doses on outcomes, and the impact of treatment strategies for respiratory viral infections.

### 2.3 Statistical Analysis

As an exploratory study, no a priori sample size calculation was performed. Data were summarized using frequencies and percentages for categorical variables and median, interquartile range (IQR), and absolute range for continuous variables. A univariable Cox regression model analyzed factors influencing mortality in HM patients with respiratory viral infections. Clinically relevant variables were considered for multivariable analysis, which was conducted using the Wald backward method. Variables were included in the multivariable Cox regression model based on a statistical significance threshold (*p* ≤ 0.05). Mortality per viral pathogen was analyzed using Kaplan–Meier survival plots, with survival probabilities compared via log‐rank test. The Cox proportional hazards model was used to analyze variables impacting mortality, including biological sex, age, vaccination status at infection onset, infecting viral pathogen, comorbidities, neutrophil and lymphocyte counts, baseline HM, HM status at infection diagnosis, last chemotherapy strategy, symptoms at infection onset, treatment strategies, hospitalization during the infection episode, and secondary infections (bacterial, fungal, or other viral). Hazard ratios (HR) and 95% confidence intervals (CI) were reported to quantify associations, with *p* ≤ 0.05 considered statistically significant. Statistical analyses were performed using SPSS version 25.0 (SPSS, IBM Corp, Chicago, IL, USA).

### 2.4 Ethics Statement

The registry received ethical approval from the local ethics committee of the Fondazione Policlinico Universitario Agostino Gemelli, IRCCS, Università Cattolica del Sacro Cuore in Rome, Italy (Study ID: 3226), as well as from the respective ethics committees of participating institutions when required. The registry was further amended at the end of 2023 and named EPICOVIDEHA‐EPIFLUEHA (Study ID: 113/23 del 03/01/2023). The anonymized data that do not contain any personally identifiable information from any sources implies that the informed consent is not applicable.

### 2.5 Role of the Funding Source

This research did not receive any funding. J.S.G., F.M., L.P., and O.A.C. had access to and verified all raw datasets and decided to submit the article.

## 3 Results

During the boreal Autumn–Winter of 2023–2024, EPICOVIDEHA‐EPIFLUEHA registered 1312 patients from 24 countries (Figures S1–S3). Among them, 48.7% (*n* = 639/1312) were diagnosed with SARS‐CoV‐2, 19.1% (*n* = 250/1312) with influenza, 12.3% (*n* = 135/1312) with RSV, and 9.8% (*n* = 128/1312) with rhinovirus. There were also cases of infections from parainfluenza (2.4%, *n* = 31/1312), metapneumovirus (2.1%, *n* = 28/1312), non‐SARS‐CoV‐2 coronaviruses (1.5%, *n* = 20/1312), and enterovirus/rhinovirus (1.4%, *n* = 18/1312). Additionally, 4.5% (*n* = 59/1312) had multiple viral infections, mostly involving influenza (44.1%, *n* = 26/59) or SARS‐CoV‐2 (57.6%, *n* = 34/59). Of these, 93.2% (*n* = 55/59) had two viruses, whereas 6.8% (*n* = 4/59) had three. The most common coinfections were influenza plus SARS‐CoV‐2 (20.3%, *n* = 12/59) and RSV plus SARS‐CoV‐2 (18.6%, *n* = 11/59) (Table 1).

**TABLE 1:** Profile of EPICOVIDEHA‐EPIFLUEHA patients during the Winter season: September 2023–March 2024.

	Overall		SARS‐CoV‐2		Influenza		RSV	
	*n* = 1312, 100.0%		*n* = 639, 48.7%		*n* = 250, 19.1%		*n* = 135, 10.3%	
	*n*	%	*n*	%	*n*	%	*n*	%
Sex								
Female	593	45.2	304	47.6	103	41.2	59	43.7
Male	719	54.8	335	52.4	147	58.8	76	56.3
Age	65 (54–73) [18–96]		66 (57–73) [19–95]		64 (55–73) [18–93]		63 (53–71) [18–87]	
Vaccination at infection onset								
Not vaccinated	1242	94.7	617	96.6	208	83.2	134	99.3
Influenza	44	3.4	0	0.0	42	16.8	0	0.0
RSV	3	0.2	0	0.0	0	0.0	1	0.7
SARS‐CoV‐2	23	1.8	22	3.4	0	0.0	0	0.0
*Days from last vaccination to infection*	79 (54–125) [0–346]		180 (31–319) [0–346]		73 (54–89) [0–344]		80 (80–80) [80–80]	
Comorbidities								
0–1	933	71.1	439	68.7	175	70.0	100	74.1
2+	379	28.9	200	31.3	75	30.0	35	25.9
*Chronic cardiopathy*	558	42.5	304	47.6	123	49.2	49	36.3
*Chronic pulmonary disease*	179	13.6	79	12.4	39	15.6	13	9.6
*Diabetes mellitus*	178	13.6	95	14.9	31	12.4	17	12.6
*Liver disease*	44	3.4	14	2.2	12	4.8	6	4.4
*Obesity (BMI > 30)*	72	5.5	36	5.6	17	6.8	10	7.4
*Renal impairment*	89	6.8	52	8.1	14	5.6	6	4.4
*Smoking history*	151	11.5	79	12.4	34	13.6	15	11.1
Neutrophils								
< 500	128	9.8	50	7.8	23	9.2	23	17.0
500–999	101	7.7	40	6.3	25	10.0	15	11.1
≥ 1000	921	70.2	454	71.0	181	72.4	84	62.2
Lymphocytes								
≤ 200	143	10.9	45	7.0	37	14.8	23	17.0
201–499	194	14.8	87	13.6	46	18.4	27	20.0
≥ 500	800	61.0	405	63.4	146	58.4	73	54.1
Baseline hematological malignancy								
Lymphoma	401	30.6	214	33.5	59	23.6	41	30.4
*Hodgkin lymphoma*	39	3.0	14	2.2	6	2.4	5	3.7
*Non‐Hodgkin lymphoma*	362	27.6	200	31.3	53	21.2	36	26.7
Plasma cell malignancies	293	22.3	140	21.9	70	28.0	31	23.0
*Amyloid light‐chain amyloidosis*	7	0.5	5	0.8	0	0.0	0	0.0
*Multiple myeloma*	286	21.8	135	21.1	70	28.0	31	23.0
Acute myeloid leukemia	259	19.7	107	16.7	57	22.8	31	23.0
Acute lymphoblastic leukemia	84	6.4	32	5.0	11	4.4	14	10.4
Chronic lymphocytic leukemia	121	9.2	78	12.2	17	6.8	10	7.4
*Chronic lymphocytic leukemia*	113	8.6	75	11.7	15	6.0	9	6.7
*Hairy cell leukemia*	8	0.6	3	0.5	2	0.8	1	0.7
Myelodysplastic syndrome	82	6.3	36	5.6	22	8.8	5	3.7
Chronic myeloid malignancies	59	4.5	26	4.1	12	4.8	2	1.5
*Chronic myeloid leukemia*	24	1.8	11	1.7	3	1.2	0	0.0
*Myelofibrosis*	20	1.5	6	0.9	7	2.8	2	1.5
*Essential thrombocythemia*	7	0.5	5	0.8	0	0.0	0	0.0
*Polycythaemia vera*	6	0.5	2	0.3	2	0.8	0	0.0
*Systemic mastocytosis*	2	0.2	2	0.3	0	0.0	0	0.0
Aplastic anemia	13	1.0	6	0.9	2	0.8	1	0.7
Status of baseline hematological malignancy								
Controlled malignancy	651	49.6	311	48.7	119	47.6	67	49.6
Active malignancy	661	50.4	328	51.3	131	52.4	68	50.4
Last chemotherapy strategy before infection diagnosis								
Conventional chemotherapy	238	18.1	101	15.8	48	19.2	32	23.7
*< 3 months*	197	15.0	84	13.1	42	16.8	26	19.3
*> 3 months*	41	3.1	17	2.7	6	2.4	6	4.4
Demethylating agents	86	6.6	36	5.6	29	11.6	8	5.9
*< 3 months*	78	5.9	32	5.0	27	10.8	8	5.9
*> 3 months*	8	0.6	4	0.6	2	0.8	0	0.0
Immuno‐chemotherapy	468	35.7	272	42.6	81	32.4	39	28.9
*< 3 months*	389	29.6	231	36.2	67	26.8	31	23.0
*> 3 months*	79	6.0	41	6.4	14	5.6	8	5.9
Targeted therapy	188	14.3	101	15.8	42	16.8	19	14.1
*< 3 months*	172	13.1	96	15.0	35	14.0	18	13.3
*> 3 months*	16	1.2	5	0.8	7	2.8	1	0.7
alloHSCT	129	9.8	36	5.6	17	6.8	17	12.6
*< 6 months*	55	4.2	14	2.2	7	2.8	8	5.9
*> 6 months*	74	5.6	22	3.4	10	4.0	9	6.7
autoHSCT	47	3.6	16	2.5	8	3.2	6	4.4
*< 6 months*	43	3.3	14	2.2	7	2.8	6	4.4
*> 6 months*	4	0.3	2	0.3	1	0.4	0	0.0
CAR‐T	15	1.1	5	0.8	2	0.8	2	1.5
*< 6 months*	11	0.8	3	0.5	1	0.4	2	1.5
*> 6 months*	4	0.3	2	0.3	1	0.4	0	0.0
No treatment	119	9.1	65	10.2	17	6.8	9	6.7
Supportive measures	23	1.8	7	1.1	7	2.8	3	2.2
Viral diagnosis month								
September 2023	86	6.6	56	8.8	0	0.0	0	0.0
October 2023	151	11.5	108	16.9	4	1.6	6	4.4
November 2023	216	16.5	151	23.6	7	2.8	13	9.6
December 2023	321	24.5	193	30.2	49	19.6	35	25.9
January 2024	280	21.3	94	14.7	113	45.2	27	20.0
February 2024	175	13.3	27	4.2	60	24.0	43	31.9
March 2024	83	6.3	10	1.6	17	6.8	11	8.1
Symptoms at viral infection onset								
No symptoms	512	39.0	169	26.4	43	17.2	90	66.7
Extrapulmonary symptoms	704	53.7	449	70.3	196	78.4	20	14.8
Pulmonary symptoms	21	1.6	0	0.0	0	0.0	9	6.7
Viral infection severity								
Asymptomatic	126	9.6	99	15.5	5	2.0	8	5.9
Mild	872	66.5	427	66.8	163	65.2	81	60.0
Severe	211	16.1	73	11.4	52	20.8	37	27.4
Critical	103	7.9	40	6.3	30	12.0	9	6.7
Viral infection treatment								
No treatment	512	39.0	169	26.4	43	17.2	90	66.7
Antivirals ± corticosteroids	704	53.7	449	70.3	196	78.4	20	14.8
Immunoglobulins	21	1.6	0	0.0	0	0.0	9	6.7
Corticosteroids	65	5.0	15	2.3	9	3.6	14	10.4
Immunoglobulins in combination	10	0.8	6	0.9	2	0.8	2	1.5
Secondary infections	242	18.4	100	15.6	36	14.4	33	24.4
Bacterial	177	13.5	74	11.6	23	9.2	27	20.0
Fungal	53	4.0	20	3.1	12	4.8	8	5.9
Other viral	53	4.0	21	3.3	4	1.6	5	3.7
Patient stay during viral infection								
Home	548	41.8	324	50.7	83	33.2	38	28.1
Hospital	739	56.3	306	47.9	162	64.8	94	69.6
*Hospital, non‐ICU*	636	48.5	266	41.6	132	52.8	85	63.0
*Hospital, ICU*	103	7.9	40	6.3	30	12.0	9	6.7
*Invasive MV*	43	41.7	15	37.5	12	40.0	6	66.7
*Noninvasive MV*	34	33.0	9	22.5	13	43.3	3	33.3
Not reported	25	24.3	9	22.5	5	16.7	3	33.3
Mortality D30	77	5.9	32	5.0	22	8.8	8	5.9
Reason for mortality								
*Hematological malignancy*	50	3.8	23	3.6	12	4.8	6	4.4
*Viral infection*	49	3.7	23	3.6	18	7.2	2	1.5
*Other reasons*	36	2.7	12	1.9	9	3.6	5	3.7

Abbreviations: alloHSCT, allogeneic hematopoietic stem cell transplantation; autoHSCT, autologous hematopoietic stem cell transplantation; BMI, body mass index; CAR‐T, chimeric antigen receptor T‐cell therapy; Dd30, Day 30; ICU, intensive care unit; MV, mechanical ventilation; *n*, number; RSV, respiratory syncytial virus; SARS‐CoV‐2, severe acute respiratory syndrome coronavirus 2.

Among the patients documented, 54.8% (*n* = 719/1312) were male. The highest male‐to‐female ratios were seen in cases of influenza (58.8%, *n* = 147/250) and enterovirus/rhinovirus infections (61.1%, *n* = 11/18). The median age of the patients was 65 years (IQR 54–73, range of 18–96). The youngest patients were those with adenovirus or bocavirus infections, with a median age of 44 years (IQR 36–59, range 32–70), while the oldest were those with SARS‐CoV‐2 infections, with a median age of 66 years (IQR 57–73, range 19–95). Regarding viral infections with an available vaccination schedule, limited to influenza H1N1 and SARS‐CoV‐2, only 5.3% (70 out of 1312) of the patients had been vaccinated against the respective virus., with the vaccination occurring a median of 79 days before the diagnosis of the infection (IQR 54–125, range 0–346). About 28.9% (*n* = 379/1312) of patients had two or more comorbidities. The most common underlying conditions were chronic heart disease (42.5%, *n* = 558/1312), chronic lung disease (13.6%, *n* = 179/1312), diabetes mellitus (13.6%, *n* = 178/1312), and a history of smoking (12.4%, *n* = 79/1312). Chronic heart disease was the most prevalent underlying condition regardless of the viral infection, although other major conditions did vary depending on the virus. Neutropenia (fewer than 500 neutrophils/mm^3^) was observed in 9.8% (*n* = 128/1312) of patients, and lymphopenia (200 or fewer lymphocytes/mm^3^) was seen in 10.9% (*n* = 143/1312) (Tables 1 and S1, and Figure S4).

**FIGURE 1:**
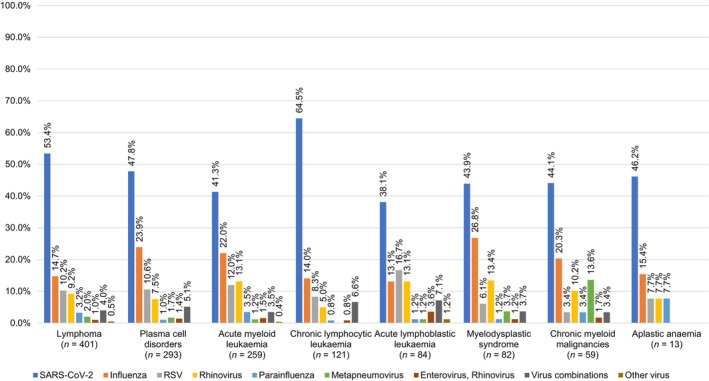
Community‐acquired respiratory viral infection distribution per baseline hematological malignancy in EPICOVIDEHA‐EPIFLUEHA participants with hematological patients diagnosed with respiratory viral infections (September 2023–March 2024).

Lymphomas were the most common underlying HM, affecting 30.6% (*n* = 401/1312) of the patients, with 27.6% (*n* = 362/1312) having non‐Hodgkin lymphoma. Other significant malignancies included plasma cell malignancies (22.3%, *n* = 293/1312) and acute myeloid leukemia (19.7%, *n* = 259/1312). Among influenza patients, plasma cell malignancies were the most common (28.0%, *n* = 70/250), whereas for metapneumovirus infections, lymphomas and plasma cell malignancies were equally prevalent (28.6%, *n* = 8/28 each). At the time of diagnosis of the viral infection, 49.6% (*n* = 651/1312) of the patients had a controlled underlying malignancy, whereas 50.4% (*n* = 661/1312) had an active malignancy. Two thirds of the patients (63.7%, *n* = 836/1312) had undergone drug‐based chemotherapy in the 3 months prior to their infection diagnosis, and 8.3% (*n* = 109/1312) had received either chimeric antigen receptor T‐cell (CAR‐T) therapy or an HSCT in the prior 6 months. Most viral infections were diagnosed between November 2023 (16.5%, *n* = 216/1312) and January 2024 (21.3%, *n* = 280/1312), with a peak in December 2023 (24.5%, *n* = 321/1312). SARS‐CoV‐2 was the most prevalent pathogen from September to December 2023 (60.1%–71.5% of all infections in the period), while influenza was more common from January to March 2024 (20.5%–40.4% of all infections in the period). RSV infections were high in February 2024 (24.6%, *n* = 43/175), and metapneumovirus infections peaked in March 2024 (18.1%, *n* = 15/83) (Tables 1 and S1 and Figure 1 and S3–S5).

Most patients experienced either asymptomatic (9.6%, *n* = 126/1312) or mild infections (66.5%, *n* = 872/1312). Critical infections were reported in 7.9% (*n* = 103/1312) of cases, with higher rates seen in parainfluenza (16.1%, *n* = 5/31), metapneumovirus (14.3%, *n* = 4/28), and influenza infections (12.0%, *n* = 30/250). A total of 56.3% (*n* = 739/1312) of patients required hospital admission, with the highest rate among those with RSV infections (69.6%, *n* = 94/135). A total of 103/1312 patients (7.9%) were admitted to the ICU, with 43/103 (41.7%) requiring mechanical ventilation. The highest ICU admission rates were seen in patients with parainfluenza (16.1%, *n* = 5/31) and metapneumovirus (14.3%, *n* = 4/22) (Table S1). No treatment was given to 39% (*n* = 512/1312) of patients. Among those who did receive treatment, the most common were antivirals, with or without corticosteroids (53.7%, *n* = 704/1312), especially for influenza (78.4%, *n* = 196/250) and SARS‐CoV‐2 infections (70.3%, *n* = 449/639). Secondary infections occurred in 18.4% (*n* = 242/1312) of patients, with bacterial infections being the most common (13.5%, *n* = 177/1312). The highest rates of secondary bacterial infections were observed in enterovirus/rhinovirus co‐infections (38.9%, *n* = 7/18), rhinovirus infections (21.1%, *n* = 27/128), and RSV infections (20.0%, *n* = 27/135). Fungal and other viral secondary infections were less common overall (4%, *n* = 53/1312 each), though they were relatively more frequent in parainfluenza (9.7%, *n* = 3/31) and metapneumovirus infections (10.7%, *n* = 3/28) (Tables 1 and S1).

The overall 30‐day mortality rate was 5.9% (77/1312). Parainfluenza infections had the highest all‐cause mortality rate at 19.4% (6/31), surpassing other common CARV such as influenza (8.8%, 22/250) and SARS‐CoV‐2 (5.0%, 32/639). The highest CARV‐attributable mortality rates were observed in influenza at 81.8% (18/22) and SARS‐CoV‐2 at 63.6% (49/77). Additionally, the progression of the underlying malignancy contributed to 64.9% (50/77) of the total deaths (Tables 1 and S1 and Figure S6).

**FIGURE 2:**
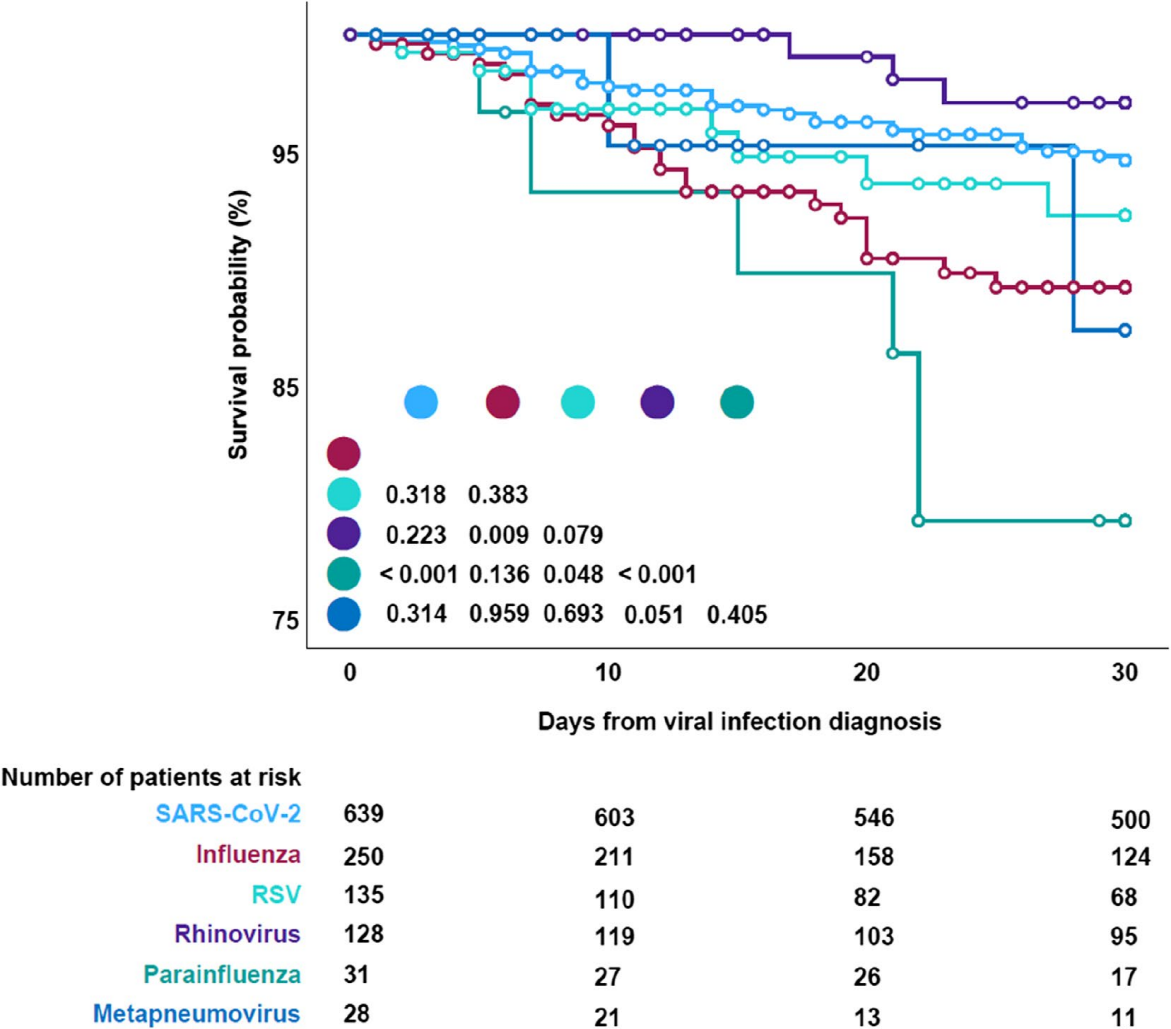
Day 30 survival probability by respiratory viral pathogen.

Moreover, a pool of patients with the most prevalent pathogenic viruses was established, including cases of monoinfection from SARS‐CoV‐2, influenza, RSV, rhinovirus, parainfluenza, and metapneumovirus. The following factors were associated with increased mortality in the multivariable analysis (Table 2): parainfluenza infection (*p* = 0.040, adjusted HR [aHR] 3.326, 95% CI 1.058–10.453), baseline smoking history (*p* = 0.028, aHR 3.867, 95% CI 1.156–12.930), secondary bacterial infection (*p* < 0.001, aHR 4.023, 95% CI 2.110–7.673), and hospital admission, regardless whether to a normal ward (*p* = 0.018, aHR 11.683, 95% CI 1.535–88.929) or to an ICU (*p* < 0.001, aHR 49.946, 95% CI 6.462–386.020). Conversely, reduced mortality was associated with the absence of lymphopenia (201–499 lymphocytes/mm^3^, *p* < 0.001, aHR 0.173, 95% CI 0.063–0.481; 500–999 lymphocytes/mm^3^, *p* = 0.002, aHR 0.381, 95% CI 0.206–0.704) (Table 2). Furthermore, Kaplan–Meier survival plots indicated significantly higher survival probabilities in SARS‐CoV‐2 patients compared with those with influenza (*p* = 0.006) or parainfluenza (*p* < 0.001), also a higher survival probability in rhinovirus patients compared with those with influenza (*p* = 0.009) and parainfluenza (*p* < 0.001), and in RSV patients compared with those with parainfluenza (*p* = 0.048) (Figure 2).

**TABLE 2:** Factors associated with increased mortality in a pool of SARS‐CoV‐2, influenza, respiratory syncytial virus, rhinovirus, parainfluenza, and metapneumovirus.

	Univariable analysis				Multivariable analysis			
	*p*	HR	95% CI		*p*	HR	95% CI	
			Lower	Upper			Lower	Upper
Sex								
Female	—	—	—	—	—	—	—	—
Male	0.110	1.478	0.915	2.386	—	—	—	—
Age	0.053	1.017	1.000	1.034	0.196	1.013	0.993	1.033
Vaccination at infection onset								
Not vaccinated	—	—	—	—	—	—	—	—
Influenza	0.258	1.790	0.653	4.909	—	—	—	—
RSV	0.971	—	—	—	—	—	—	—
SARS‐CoV‐2	0.712	0.690	0.096	4.968	—	—	—	—
Viruses								
SARS‐CoV‐2	—	—	—	—	—	—	—	—
Influenza	0.006*	2.138	1.241	3.681	0.062	1.985	0.966	4.077
RSV	0.312	1.492	0.687	3.240	0.595	0.770	0.294	2.019
Rhinovirus	0.232	0.486	0.149	1.587	0.162	0.343	0.077	1.538
Parainfluenza	0.001*	4.234	1.770	10.130	0.040*	3.326	1.058	10.453
Metapneumovirus	0.320	2.064	0.494	8.621	0.507	1.674	0.365	7.666
Comorbidities								
0–1	—	—	—	—	—	—	—	—
2+	0.228	1.343	0.832	2.168	—	—	—	—
*Chronic cardiopathy*	0.303	0.786	0.497	1.243	—	—	—	—
*Chronic pulmonary disease*	0.599	0.842	0.443	1.598	—	—	—	—
*Diabetes mellitus*	0.071	0.593	0.336	1.046	—	—	—	—
*Liver disease*	0.014*	0.352	0.152	0.810	0.073	0.372	0.126	1.097
*Obesity (BMI > 30)*	0.992	1.005	0.367	2.754	—	—	—	—
*Renal impairment*	0.938	1.037	0.418	2.570	—	—	—	—
*Smoking history*	0.046*	3.247	1.022	10.311	0.028*	3.867	1.156	12.930
Neutrophils								
< 500	—	—	—	—	—	—	—	—
500–999	0.277	0.552	0.189	1.614	0.878	1.096	0.340	3.532
≥ 1000	0.089	0.551	0.278	1.094	0.249	1.671	0.698	4.000
Lymphocytes								
≤ 200	—	—	—	—	—	—	—	—
201–499	< 0.001*	0.179	0.072	0.445	< 0.001*	0.173	0.063	0.481
≥ 500	< 0.001*	0.213	0.122	0.372	0.002*	0.381	0.206	0.704
Baseline hematological malignancy								
Lymphoma	—	—	—	—	—	—	—	—
Plasma cell malignancies	0.136	0.583	0.287	1.185	—	—	—	—
Acute myeloid leukemia	0.182	0.607	0.291	1.263	—	—	—	—
Chronic lymphocytic leukemia	0.917	1.043	0.471	2.313	—	—	—	—
Acute lymphoblastic leukemia	0.862	1.089	0.417	2.844	—	—	—	—
Myelodysplastic syndrome	0.504	1.331	0.576	3.077	—	—	—	—
Chronic myeloid malignancies	0.105	2.001	0.865	4.628	—	—	—	—
Aplastic anemia	0.970	—	—	—	—	—	—	—
Status hematological malignancy at infection onset								
Controlled malignancy	—	—	—	—	—	—	—	—
Active malignancy	< 0.001*	3.940	2.233	6.951	0.068	1.850	0.956	3.579
Last chemotherapy strategy before infection								
Conventional chemotherapy	—	—	—	—	—	—	—	—
Demethylating agents	0.574	0.753	0.279	2.027	0.479	0.558	0.111	2.799
Immunochemotherapy	0.194	0.669	0.365	1.226	0.259	0.611	0.260	1.436
Targeted therapy	0.431	0.740	0.349	1.566	0.651	0.787	0.278	2.224
alloHSCT	0.033*	0.204	0.047	0.878	0.876	0.869	0.149	5.061
autoHSCT	0.962	—	—	—	0.975	—	—	—
CAR‐T	0.966	1.045	0.139	7.828	0.997	—	—	—
No treatment	0.767	1.124	0.519	2.434	0.243	1.852	0.659	5.210
Supportive measures	0.627	0.607	0.081	4.549	0.552	0.492	0.047	5.105
Symptoms at viral infection onset								
No symptoms	—	—	—	—	—	—	—	—
Extrapulmonary symptoms	0.275	0.631	0.276	1.441	0.243	0.551	0.203	1.498
Pulmonary symptoms	0.006*	2.929	1.371	6.260	0.665	1.223	0.492	3.042
Infection treatment								
No treatment	—	—	—	—	—	—	—	—
Antivirals ± corticosteroids	0.282	1.333	0.789	2.251	0.115	1.986	0.846	4.663
Immunoglobulins	0.723	1.437	0.193	10.689	0.360	3.142	0.271	36.402
Corticosteroids	0.008*	3.187	1.355	7.496	0.216	1.955	0.677	5.645
Immunoglobulins in combination	0.031*	4.929	1.155	21.032	0.169	3.457	0.591	20.222
Secondary bacterial infection	< 0.001*	4.605	2.881	7.359	< 0.001*	4.023	2.110	7.673
Secondary fungal infection	< 0.001*	4.406	2.320	8.366	0.331	1.524	0.652	3.565
Secondary viral infection	0.212	1.782	0.719	4.420	—	—	—	—
Patient stay during infection episode								
Home	—	—	—	—	—	—	—	—
Hospital, non‐ICU	< 0.001*	21.494	5.206	88.743	0.018*	11.683	1.535	88.929
Hospital, ICU	< 0.001*	102.053	24.298	428.622	< 0.001*	49.946	6.462	386.020
Not reported	0.971	—	—	—	0.977	—	—	—

*Note*: *Statistically significant difference.

Abbreviations: alloHSCT, allogeneic hematopoietic stem cell transplant; autoHSCT, autologous hematopoietic stem cell transplant; BMI, body mass index; CAR‐T, chimeric antigen receptor T‐cell; CI, confidence interval; HR, hazard ratio; ICU, intensive care unit; RSV, respiratory syncytial virus; SARS‐CoV‐2, severe acute respiratory syndrome coronavirus 2.

Sensitivity analyses were conducted to identify factors associated with increased mortality in patients with SARS‐CoV‐2, influenza, and RSV infections, respectively. For SARS‐CoV‐2 infections, Cox multivariable regression analysis identified myelodysplastic syndrome (*p* = 0.005, aHR 6.102, 95% CI 1.705–21.842), active malignancy (*p* = 0.036, aHR 3.884, 95% CI 1.093–13.519), fungal secondary infection (*p* = 0.037, aHR 3.761, 95% CI 1.084–13.051), and hospital admission, either in a non‐ICU (*p* = 0.014, aHR 13.319, 95% CI 1.704–104.137) or ICU ward (*p* = 0.001, aHR 39.351, 95% CI 4.384–353.185), as factors associated with increased mortality. Conversely, the absence of lymphopenia was a protective factor (201–499 lymphocytes/mm^3^, *p* = 0.011 aHR 0.177, 95% CI 0.047–0.672; 500–999 lymphocytes/mm^3^, *p* = 0.001, aHR 0.170, 95% CI 0.059–0.492). In influenza patients, secondary bacterial infection was associated with increased mortality in the multivariable analysis (*p* < 0.001, aHR 10.837, 95% CI 4.085–28.750), while the absence of lymphopenia was protective (201–499 lymphocytes/mm^3^, *p* = 0.008, aHR 0.058, 95% CI 0.007–0.479; 500–999 lymphocytes/mm^3^, *p* = 0.006, aHR 0.260, 95% CI 0.099–0.684). For RSV infections, multivariable analysis indicated that secondary bacterial (*p* = 0.006, aHR 9.830, 95% CI 1.902–50.815) and viral (*p* = 0.031, aHR 6.298, 95% CI 1.182–33.551) infections were associated with an increased risk of mortality (Tables S2–S4).

## 4 Discussion

Our study highlights the major burden of CARV infections in HM patients, with SARS‐CoV‐2, influenza, and RSV being the most prevalent. Increased mortality has been seen to be linked to active HM, secondary bacterial infections, and ICU admission, while the absence of lymphopenia offers protection. The findings reveal that the impact of these infections varied by CARV, underscoring the need for tailored management strategies. This stresses the importance of adapting treatment approaches and maintaining vigilant monitoring to address the evolving nature of CARV infections in HM patients.

Pathogen distribution and seasonal peaks align with known CARV circulation trends in the Northern hemisphere [2, 3, 5, 6, 7, 32]. From September to December 2023, SARS‐CoV‐2 was the most prevalent virus in our patients, likely driven by highly transmissible variants [33], potentially insufficient vaccine coverage [34, 35], and increased social activities and gatherings during the holidays [36]. Of note, SARS‐CoV‐2 infection levels have been described to be less affected by environmental temperature changes [37]. Conversely, influenza increased from January to March 2024, reflecting its typical seasonal rise [38] and reclaiming prominence after recent years where SARS‐CoV‐2 was the dominant CARV [39, 40]. This shift may be linked to the relaxing of COVID‐19‐related precautions that had previously kept influenza rates low. RSV and metapneumovirus peaked in February and March 2024, respectively, while rhinovirus remained consistently present but at low levels throughout the study. This multi‐pathogen scenario highlights the need for comprehensive preventive measures and increased vigilance, particularly for high‐risk groups such as patients with HM.

The study found a limited vaccination rate for influenza, RSV, and SARS‐CoV‐2—CARV with currently available vaccines— [41] highlighting a major gap in preventive care. This is especially concerning for HM patients, who are at higher risk for severe infection, as compared with the general population. In parallel, the median time between vaccination and infection was 79 days, which could suggest a waning immunity over time. To overcome the situation, strategies like more frequent boosters [42] or passive immunization might be necessary [18]. Besides, understanding the reasons for low vaccination rates—such as safety concerns [43], scheduling conflicts with antineoplastic treatment [44], lack of awareness [45], or vaccine hesitancy [46, 47]—is also crucial. Improving education, refining vaccination protocols, and monitoring immune responses could help increase vaccination rates and better protect these patients [48].

Our study cohort was characterized by a high prevalence of lymphomas, particularly non‐Hodgkin lymphoma, followed by plasma cell malignancies and acute myeloid leukemia. This distribution matches patterns seen previously in CARV [2, 23]. Interestingly, we found no significant differences in 30‐day mortality based on malignancy treatment type, contrasting with some previous reports [6, 32, 49, 50]. Most of our patients had received drug‐based chemotherapy in the past 3 months, indicating uniformly high immunosuppression. Although recent allogeneic HSCT is linked to higher CARV mortality in the literature [6, 32, 49, 50, 51], our study's broader timeframe (6 months) may account for this discrepancy, which also exists in the literature, with reports describing the lack of correlation between HSCT and increased CARV‐related mortality [52, 53]. Despite this, the high rate of active HM causing severe immune dysregulation, so as recent treatments likely contributed to increased vulnerability to viral infections, aligning with existing literature [54].

In addition to HM, a large number of patients had other underlying conditions that increased their risk of severe CARV infections. Chronic heart disease was the most common, followed by chronic lung disease, diabetes, and a history of smoking. These conditions weaken the immune system and complicate CARV infection management. Chronic heart disease was notably prevalent across all viral infections studied. Multiple comorbidities complicate treatment, possibly requiring specific antiviral medications or changes to malignancy treatment regimens. This highlights the need for a multidisciplinary approach involving not only infectious diseases and hematology but also cardiology, pulmonology, or endocrinology. Lymphopenia and prior corticosteroid use are also known to worsen outcomes in patients with CARV and HM [6, 7, 55]. Our study found that severe lymphopenia significantly increased mortality, but we did not collect data on corticosteroid use, limiting our analysis. Notably, smoking history emerged as a significant risk factor for mortality, a finding not widely reported. While smoking has been linked to severe RSV infections in HSCT patients [56], this was not observed in our study. Additionally, we did not find a significant link between neutropenia and mortality, despite prior research suggesting it correlates with RSV progression to lower respiratory tract infections [56].

Most patients had asymptomatic or mild infections, yet nearly 10% of the total required ICU admission, and half of those needed mechanical ventilation. Infection severity varied by pathogen, with influenza, parainfluenza, and metapneumovirus associated with higher rates of severe infection and ICU stays, in line with previous research [52, 53, 55, 57, 58, 59]. This variation could stem from virus pathogenicity, the level of patient immunosuppression, or existing underlying comorbidities. Notably, 39% of patients did not receive antiviral treatment, likely due to the mild nature of the infections, the lack of effective treatments [2], or concerns about drug interactions and toxicity [2]. Antiviral therapy, often combined with corticosteroids, was common for influenza and SARS‐CoV‐2, where effective options exist [25], improving management, outcomes, and burden to the healthcare system. The lack of treatment options for viruses like metapneumovirus, parainfluenza, or RSV highlight the urgent need for improved antiviral prophylaxis and therapies for CARV infections, particularly in HM patients.

Secondary infections occurred in one in five patients, with bacterial infections being the most common, especially in those with rhinovirus and RSV infections. Bacterial coinfections complicate viral infections in HM patients, increasing morbidity and mortality [54, 55, 60, 61, 62, 63]. The high rate of bacterial infections indicates that viral infections often impair mucosal barriers and immune responses, leading to bacterial overgrowth. In our study, bacterial secondary infections were linked to a four‐fold increase in mortality risk, consistent with another research [54, 55, 64]. While less common, the risk of secondary fungal and viral infections highlights the severe immune suppression in this group, particularly after recent treatments like chemotherapy, HSCT, or CAR‐T therapy. Managing these infections requires a careful balance of timely antimicrobial treatment and resistance prevention.

The overall 30‐day mortality rate was 6%, with significant variation among viruses. Both, the overall and attributable mortality rates were highest for parainfluenza, followed by influenza and metapneumovirus. The high mortality with parainfluenza, despite its lower prevalence, highlights the need for better antiviral options and the high vulnerability of HM patients. Key mortality risk factors included parainfluenza infection, smoking history, secondary bacterial infections, and hospital admission, particularly to the ICU, all of which have been widely reported in the literature [2, 3, 6, 7, 32, 54, 55, 56, 65, 66]. Conversely, the absence of lymphopenia was linked to lower mortality, suggesting that maintaining lymphocyte counts may be protective. Progression of the underlying malignancy was responsible for about 65% of deaths, but our focus was on overall mortality, as attributable mortality can be influenced by subjective clinical judgment.

Our registry study has several notable limitations. First, the study design did not allow us to calculate the incidence of CARV by HM type, as we lacked the necessary denominator, hindering our understanding of type‐specific CARV rates. Second, there may be an underestimation of CARV cases due to a likely bias toward documenting more severe infections, which could lead to overestimating their severity. Third, with only about 5% of patients vaccinated, our study was limited in assessing vaccine effectiveness for preventing severe disease or modifying infection outcomes. This finding underscores the urgent need to enhance vaccination efforts among HM patients and their close contacts—including family members and healthcare workers—especially given the seasonal peaks of these respiratory infections. The cyclical pattern and respiratory transmission of CARVs like SARS‐CoV‐2 and influenza indicate that coordinating vaccinations with peak viral circulation periods could offer greater protection. The low vaccination rate reported in our cohort may partly result from underreporting in medical records but also highlights a preventive gap that could leave many patients vulnerable. Expanding vaccine coverage and providing timely boosters when available could strengthen immunity during high‐risk periods, reducing the impact of severe CARV infections in this high‐risk group. Fourth, the observational nature of the study introduces potential selection and reporting biases. Additionally, differences in healthcare access and prescription practices across the participating countries complicate the analysis of CARV treatments, particularly given the lack of standardized protocols for managing these infections. Furthermore, the study did not include detailed data on specific viral strains or variants, which could influence the outcomes, especially for SARS‐CoV‐2 and influenza.

In conclusion, our research shows how CARV pose a significant risk to HM patients, with elevated mortality linked to active malignancies, secondary bacterial infections, ICU admissions, and lymphopenia. The study observed seasonal peaks, with SARS‐CoV‐2 dominating late 2023 and influenza in early 2024, emphasizing the need for seasonally adjusted preventive strategies. Low vaccination rates among HM patients are concerning, highlighting the need for improved vaccine strategies. High comorbidity prevalence, particularly chronic heart and lung diseases, necessitates a multidisciplinary care approach. Severe CARV cases often required ICU care, underscoring the urgent need for better antiviral treatments, particularly for parainfluenza and metapneumovirus, while secondary bacterial infections significantly increased mortality risk.

## Author Contributions

J.S.‐G., F.M., L.P., and O.A.C. contributed to the study design and study supervision. J.S.G. did the statistical plan and analysis. J.S.‐G., F.M., and L.P. interpreted the data and wrote the article. All the authors recruited, and documented participants, critically read, reviewed, and agreed to publish the article. All authors had full access to the data and had final responsibility for the decision to submit for publication.

## Conflicts of Interest

The authors declare no conflicts of interest.

## Supporting information


**Data S1** Supporting Information.

## Data Availability

The corresponding author can provide the data supporting the findings of this study upon a reasonable request.
