# Supplementary material for: Respiratory Viruses in Patients With Hematological Malignancy in Boreal Autumn/Winter 2023–2024: EPICOVIDEHA‐EPIFLUEHA Report
Source: Am J Hematol. 2024 Dec 23;100(3):358–74. doi: 10.1002/ajh.27565 (PMC11803548; doi:10.1002/ajh.27565)
Supplement: Supplementary file 1 — Data S1 Supporting Information. [file AJH-100-358-s001.docx]

**Respiratory Viruses in Patients with Haematological Malignancy in Boreal Autumn/Winter 2023-2024: EPICOVIDEHA-EPIFLUEHA Report**

Jon **SALMANTON-GARCÍA**, 1 * § Francesco **MARCHESI**, 2 * Milan **NAVRÁTIL**, 3 Klára **PIUKOVICS**, 4 Maria Ilaria **DEL PRINCIPE**, 5 Marianna **CRISCUOLO**, 6 Yavuz M. **BILGIN**, 7 Nicola S. **FRACCHIOLLA**, 8 Antonio **VENA**, 9 Alessandra **ROMANO**, 10 Iker **FALCES-ROMERO**, 11 Nicola **SGHERZA**, 12 Inmaculada **HERAS**, 13 Monika M. **BIERNAT**, 14 Verena **PETZER**, 15 Pavel **ŽÁK**, 16 Barbora **WEINBERGEROVÁ**, 17 Michail **SAMARKOS**, 18 Nurettin **ERBEN**, 19 Jens **VAN PRAET**, 20 Alberto **LÓPEZ-GARCÍA**, 21 Jorge **LABRADOR**, 22 Tobias **LAHMER**, 23 Ľuboš **DRGOŇA**, 24 Maria **MERELLI**, 25 Annarosa **CUCCARO**, 26 Sonia **MARTÍN-PÉREZ**, 27 Julio **DÁVILA-VALLS**, 28 Francesca **FARINA**, 29 Chiara **CATTANEO**, 30 László Imre **PINCZÉS**, 31 Ferenc **MAGYARI**, 32 Ildefonso **ESPIGADO**, 33 Caterina **BUQUICCHIO**, 34 Donald C. **VINH**, 35 Igor **STOMA**, 36 Martin **ČERŇAN**, 37 Lucia **PREZIOSO**, 38 Mario Virgilio **PAPA**, 39 Gaëtan **PLANTEFEVE**, 40 Reham Abdelaziz **KHEDR**, 41 Josip **BATINIĆ**, 42 Gabriele **MAGLIANO**, 43 Simge **ERDEM**, 44 Sofya **KHOSTELIDI**, 45 Natasha **ČOLOVIĆ**, 46 Davide **NAPPI**, 47 Patricia **GARCÍA-RAMÍREZ**, 48 Jakub **GÓRA**, 49 Marta **CALLEJAS-CHARAVIA**, 50 Jędrzej **TŁUSTY**, 51 Martijn **BAKKER**, 52 Elwira **WOJTYNIAK**, 53 Darko **ANTIĆ**, 54 Agnieszka **MAGDZIAK**, 55 Michelina **DARGENIO**, 56 Larisa **IDRIZOVIĆ**, 57 Nikola **PANTIĆ**, 58 Zlate **STOJANOSKI**, 59 Noha **EISA**, 60 Vladimir **OTAŠEVIĆ**, 61 Monia **MARCHETTI**, 62 Erica **MACKENZIE**, 63 Carolina **GARCIA-VIDAL**, 64 Avinash **AUJAYEB**, 65 Ahlam **ALMASARI**, 66 Carolina **MIRANDA-CASTILLO**, 67 Eleni **GAVRIILAKI**, 68 Nicola **COPPOLA**, 69 Alessandro **BUSCA**, 70 Tatjana **ADŽIĆ-VUKIČEVIĆ**, 71 Martin **SCHÖNLEIN**, 72 Ditte Stampe **HERSBY**, 73 Stefanie K. **GRÄFE**, 74 Andreas **GLENTHØJ**, 75 Tommaso Francesco **AIELLO**, 76 Milche **CVETANOSKI**, 77 Mirjana **MITROVIĆ**, 78 Claudio **CERCHIONE**, 79 Romane **PRIN**, 80 Gina **VARRICCHIO**, 81 Elena **ARELLANO**, 82 Raúl **CÓRDOBA**, 83 Jiří **MAYER**, 84 Benjamín **VÍŠEK**, 85 Dominik **WOLF**, 86 Amalia N. **ANASTASOPOULOU**, 87 Mario **DELIA**, 88 Pellegrino **MUSTO**, 89 Dario **LEOTTA**, 90 Martina **BAVASTRO**, 91 Alessandro **LIMONGELLI**, 92 Mariarita **SCIUMÈ**, 93 Lukas **VAN DEN VEN**, 94 Luana **FIANCHI**, 95 Sara Caterina **BRUNETTI**, 96 Joanna **DROZD-SOKOŁOWSKA**, 97 Anna **DĄBROWSKA-IWANICKA**, 98 Oliver A. **CORNELY**, 99 # Livio **PAGANO**, 100 # §

* shared junior authorship

# shared senior authorship

§ corresponding author

**Affiliations**

1. University of Cologne, Faculty of Medicine, and University Hospital Cologne, Institute of Translational Research, Cologne Excellence Cluster on Cellular Stress Responses in Aging-Associated Diseases (CECAD), Cologne, Germany

University of Cologne, Faculty of Medicine, University Hospital Cologne, Department I of Internal Medicine, Center for Integrated Oncology Aachen Bonn Cologne Duesseldorf (CIO ABCD) and Excellence Center for Medical Mycology (ECMM), Cologne, Germany

German Centre for Infection Research (DZIF), Partner Site Bonn-Cologne, Cologne, Germany

1. Hematology and Stem Cell Transplant Unit, IRCCS Regina Elena National Cancer Institute, Rome, Italy
2. Department of Haematooncology, University Hospital Ostrava, Czech Republic

Department of Haematooncology, Faculty of Medicine, University of Ostrava, Czech Republic

1. Department of Hematology, South Division of Internal Medicine Clinic, Albert Szent-Györgyi Health Center, University of Szeged, Szeged, Hungary
2. Hematology, Department of Biomedicine and Prevention, University of Rome Tor Vergata, Rome, Italy
3. Hematology Unit, Fondazione Policlinico Universitario Agostino Gemelli - IRCCS, Rome, Italy
4. Department of Internal Medicine, ADRZ, Goes, Netherlands
5. Hematology Unit, Fondazione IRCCS Ca' Granda Ospedale Maggiore Policlinico, Milan, Italy
6. Department of Health Sciences (DISSAL), University of Genoa, Genoa, Italy

UO Clinica Malattie Infettive, IRCCS Ospedale Policlinico San Martino, Genoa, Italy

1. AOU Policlinico Rodolico San Marco, Catania, Italy
2. Microbiology and Parasitology Department, University Hospital La Paz, Madrid, Spain

CIBERINFEC, Instituto de Salud Carlos III, Madrid, Spain

1. Hematology and Stem Cell Transplantation Unit, AOUC Policlinico, Bari, Italy
2. Hospital Morales Messeguer, Department of Hematology and Oncology, Murcia, Spain
3. Department of Hematology, Blood Neoplasms, and Bone Marrow Transplantation Wroclaw Medical University, Poland
4. Department of Hematology and Oncology, Comprehensive Cancer Center Innsbruck (CCCI), Medical University of Innsbruck (MUI), Innsbruck, Austria
5. University Hospital Hradec Králové, Hradec Králové, Czech Republic
6. Masaryk University and University Hospital Brno - Department of Internal Medicine, Hematology and Oncology, Brno, Czech Republic
7. Laikon Hospital, Medical School, National and Kapodistrian University of Athens, Athens, Greece
8. Department of Infectious Diseases and Clinical Microbiology, Faculty of Medicine Eskisehir Osmangazi University, Eskisehir, Turkey
9. Department of Nephrology and Infectious diseases, AZ Sint-Jan Brugge-Oostende AV, Brugge, Belgium
10. Fundacion Jimenez Diaz University Hospital, Health Research Institute IIS-FJD, Madrid, Spain
11. Department of Hematology, Research Unit, Hospital Universitario de Burgos, Burgos, Spain
12. Medizinische Klinik II, Klinikum rechts der Isar, TU München, Munich, Germany
13. Comenius University and National Cancer Institute, Bratislava, Slovakia
14. Azienda Sanitaria Universitaria del Friuli Centrale, Udine, Italy
15. Hematology Unit, Center for Translational Medicine, AziendaUSL Toscana NordOvest, Livorno, Italy

National Cancer Institute, Fondazione ‘G. Pascale’, IRCCS, Hematology-Oncology and Stem Cell Transplantation Unit, Naples, Italy

1. Hospital Nuestra Señora de Sonsoles, Ávila, Spain
2. Hospital Nuestra Señora de Sonsoles, Ávila, Spain
3. IRCCS Ospedale San Raffaele, Milan, Italy
4. Hematology Unit, ASST-Spedali Civili, Brescia, Italy
5. Division of Hematology, Department of Internal Medicine, University of Debrecen, Debrecen, Hungary
6. Division of Hematology, Department of Internal Medicine, University of Debrecen, Debrecen, Hungary
7. Department of Hematology, University Hospital Virgen Macarena - University Hospital Virgen del Rocío, Instituto de Biomedicina de Sevilla (IBIS / CSIC), Universidad de Sevilla (Departamento de Medicina), Seville, Spain
8. Ematologia con Trapianto, Ospedale Dimiccoli Barletta, Barletta, Italy
9. McGill University Health Centre, Montreal, Canada
10. Gomel State Medical University, Gomel, Belarus
11. University Hospital Olomouc, Olomouc, Czech Republic
12. Hospital University of Parma - Hematology and Bone Marrow Unit, Parma, Italy
13. Azienda Ospedaliera Sant'Anna e San Sebastiano, Caserta, Italy
14. Head ICU and CRC, Centre Hospitalier Victor DUPOUY, Argenteuil, France
15. Department of Pediatric Oncology, National Cancer Institute, Cairo University, Cairo, Egypt

Department of Pediatric Oncology, Children's Cancer Hospital, Cairo, Egypt

1. University Hospital Centre Zagreb, Zagreb, Croatia

Croatian Cooperative Group for Hematological Diseases (CROHEM), Croatia

Faculty of Medicine University of Zagreb, Zagreb, Croatia

1. ASST Grande Ospedale Metropolitano Niguarda, Milan, Italy
2. Istanbul University, Faculty of Medicine, Department of Internal Medicine, Division of Hematology, Istanbul, Turkey
3. North-Western State Medical University named after Iliá Ilich Méchnikov, Saint-Petersburg, Russia
4. University Clinical Center Serbia, Belgrade, Serbia
5. Hematology Unit, Istituto Scientifico Romagnolo per lo Studio e la Cura dei Tumori (IRST) IRCCS, Meldola, Italy
6. Servicio de Hematología y Hemoterapia, Hospital Universitario Príncipe de Asturias, Alcalá de Henares, Madrid
7. Medical University of Warsaw, Warszawa, Poland
8. Servicio de Hematología y Hemoterapia, Hospital Universitario Príncipe de Asturias, Alcalá de Henares, Madrid
9. Medical University of Warsaw, Warszawa, Poland
10. University Medical Center Groningen, Groningen, Netherlands
11. Department of Clinical Microbiology, Maria Skłodowska-Curie National Research Institute of Oncology, Warszawa, Poland
12. University Clinical Center of Serbia, Belgrade, Serbia
13. Department of Clinical Microbiology, Maria Skłodowska-Curie National Research Institute of Oncology, Warszawa, Poland
14. Ospedale Vito Fazzi, Lecce, Italy
15. University of Cologne, Faculty of Medicine, and University Hospital Cologne, Institute of Translational Research, Cologne Excellence Cluster on Cellular Stress Responses in Aging-Associated Diseases (CECAD), Cologne, Germany

University of Cologne, Faculty of Medicine, University Hospital Cologne, Department I of Internal Medicine, Center for Integrated Oncology Aachen Bonn Cologne Duesseldorf (CIO ABCD) and Excellence Center for Medical Mycology (ECMM), Cologne, Germany

1. University Clinical Center of Serbia, Belgrade, Serbia
2. University Clinic of Hematology, Skopje, North Macedonia
3. Faculty of Medicine, Mansoura University, Mansoura, Egypt

King Faisal specialist Hospital, Jeddah, Saudi Arabia

1. University Clinical Center of Serbia, Belgrade, Serbia
2. Hematology and Transplant Unit, Azienda Ospedaliera SS Antonio e Biagio e Cesare Arrigo, Alessandria, Italy
3. University of Kansas Medical Center, Kansas City, United States
4. Department of Infectious Diseases, Hospital Clinic de Barcelona, University of Barcelona, IDIBAPS, Barcelona, Spain
5. Northumbria Healthcare, Newcastle, United Kingdom
6. King Faisal Specialist Hospital, Jeddah, Saudi Arabia
7. Hospital Rey Juan Carlos, Móstoles, Spain
8. General Hospital of Thessaloniki "George Papanikolaou", Thessaloniki, Greece
9. Department of Mental Health and Public Medicine, Universitry of Campania, Naples, Italy
10. Stem Cell Transplant Center, AOU Citta' della Salute e della Scienza, Turin, Italy
11. COVID hospital "Batajnica", Belgrade, Serbia
12. Department of Oncology, Hematology and Bone Marrow Transplantation with Section of Pneumology, University Medical Center Hamburg-Eppendorf, Hamburg, Germany
13. Department of Hematology, Copenhagen University Hospital - Rigshospitalet, Copenhagen, Denmark
14. University Medical Center Hamburg-Eppendorf, Hamburg, Germany
15. Department of Hematology, Copenhagen University Hospital - Rigshospitalet, Copenhagen, Denmark
16. Department of Infectious Diseases, Hospital Clinic de Barcelona, University of Barcelona, IDIBAPS, Barcelona, Spain
17. University Clinic of Hematology, Skopje, North Macedonia
18. University Clinical Center of Serbia, Belgrade, Serbia
19. Hematology Unit, Istituto Scientifico Romagnolo per lo Studio e la Cura dei Tumori (IRST) IRCCS, Meldola, Italy
20. CRA from CRC Centre Hospitalier Victor DUPOUY, Argenteuil, France
21. Azienda Ospedaliera Sant'Anna e San Sebastiano, Caserta, Italy
22. Department of Hematology, University Hospital Virgen Macarena - University Hospital Virgen del Rocío, Instituto de Biomedicina de Sevilla (IBIS / CSIC), Universidad de Sevilla (Departamento de Medicina), Seville, Spain
23. Fundacion Jimenez Diaz University Hospital, Health Research Institute IIS-FJD, Madrid, Spain
24. Masaryk University and University Hospital Brno - Department of Internal Medicine, Hematology and Oncology, Brno, Czech Republic
25. University Hospital Hradec Králové, Hradec Králové, Czech Republic
26. Department of Hematology and Oncology, Comprehensive Cancer Center Innsbruck (CCCI), Medical University of Innsbruck (MUI), Innsbruck, Austria
27. Laikon Hospital, Medical School, National and Kapodistrian University of Athens, Athens, Greece
28. Hematology and Stem Cell Transplantation Unit, AOUC Policlinico, Bari, Italy
29. Hematology and Stem Cell Transplantation Unit, AOUC Policlinico, Bari, Italy
30. AOU Policlinico Rodolico San Marco, Catania, Italy
31. Department of Health Sciences (DISSAL), University of Genoa, Genoa, Italy

UO Clinica Malattie Infettive, IRCCS Ospedale Policlinico San Martino, Genoa, Italy

1. Department of Health Sciences (DISSAL), University of Genoa, Genoa, Italy

UO Clinica Malattie Infettive, IRCCS Ospedale Policlinico San Martino, Genoa, Italy

1. Hematology Unit, Fondazione IRCCS Ca' Granda Ospedale Maggiore Policlinico, Milan, Italy
2. University of Cologne, Faculty of Medicine, and University Hospital Cologne, Institute of Translational Research, Cologne Excellence Cluster on Cellular Stress Responses in Aging-Associated Diseases (CECAD), Cologne, Germany

University of Cologne, Faculty of Medicine, University Hospital Cologne, Department I of Internal Medicine, Center for Integrated Oncology Aachen Bonn Cologne Duesseldorf (CIO ABCD) and Excellence Center for Medical Mycology (ECMM), Cologne, Germany

1. Hematology Unit, Fondazione Policlinico Universitario Agostino Gemelli - IRCCS, Rome, Italy
2. Hematology Unit, Fondazione Policlinico Universitario Agostino Gemelli - IRCCS, Rome, Italy
3. Medical University of Warsaw, Warszawa, Poland
4. Maria Skłodowska-Curie Institute of Oncology, Warszawa, Poland
5. University of Cologne, Faculty of Medicine, and University Hospital Cologne, Institute of Translational Research, Cologne Excellence Cluster on Cellular Stress Responses in Aging-Associated Diseases (CECAD), Cologne, Germany

University of Cologne, Faculty of Medicine, University Hospital Cologne, Department I of Internal Medicine, Center for Integrated Oncology Aachen Bonn Cologne Duesseldorf (CIO ABCD) and Excellence Center for Medical Mycology (ECMM), Cologne, Germany

German Centre for Infection Research (DZIF), Partner Site Bonn-Cologne, Cologne, Germany

University of Cologne, Faculty of Medicine and University Hospital Cologne, Clinical Trials Centre Cologne (ZKS Köln), Cologne, Germany

1. Hematology Unit, Fondazione Policlinico Universitario Agostino Gemelli - IRCCS, Rome, Italy

Hematology Unit, Università Cattolica del Sacro Cuore, Rome, Italy

**Corresponding authors**

Dr Jon Salmanton-García

University of Cologne, Faculty of Medicine and University Hospital Cologne, Translational Research, Cologne Excellence Cluster on Cellular Stress Responses in Aging-Associated Diseases (CECAD)

Herderstrasse 52

50931 Cologne, Germany

Tel: (+49) 221 478 32290

Email: [jon.salmanton-garcia@uk-koeln.de](mailto:jon.salmanton-garcia@uk-koeln.de)

Prof. Dr Livio Pagano

Fondazione Policlinico Universitario A. Gemelli - IRCCS - Università Cattolica del Sacro Cuore

Largo Francesco Vito 1

00168 Rome, Italy

E-mail: [livio.pagano@unicatt.it](mailto:livio.pagano@unicatt.it)

**ABSTRACT**

**~~Introduction~~**Community-acquired respiratory viral infections (CARV) significantly impact patients with haematological malignancies (HM), leading to high morbidity and mortality. However, large-scale, real-world data on CARV in these patients is limited. **~~Methods~~**This study analysed data from the EPICOVIDEHA-EPIFLUEHA registry, focusing on patients with HM diagnosed with CARV during the 2023-2024 autumn-winter season. The study assessed epidemiology, clinical characteristics, risk factors, and outcomes. **~~Results~~**The study examined 1,312 patients with HM diagnosed with CARV during the 2023-2024 autumn-winter season. Of these, 59.5% required hospitalization, with 13.5% needing ICU admission. The overall mortality rate was 10.6%, varying by virus: parainfluenza (21.3%), influenza (8.8%), metapneumovirus (7.1%), RSV (5.9%), or SARS-CoV-2 (5.0%). Poor outcomes were significantly associated with smoking history, severe lymphopenia, secondary bacterial infections, and ICU admission. **~~Conclusions~~**This study highlights the severe risk CARV poses to patients with HM, especially those undergoing active treatment. The high rates of hospitalization and mortality stress the need for better prevention, early diagnosis, and targeted therapies. Given the severe outcomes with certain viruses like parainfluenza, tailored strategies are crucial to improving patient outcomes in future CARV seasons.

**Funding**

Not applicable

**Keywords**

community-acquired respiratory viral infection; vaccine coverage; haematological malignancy; antiviral therapy; secondary infection

**INTRODUCTION**

Community-acquired respiratory viral infections (CARV) are a significant concern for patients with haematological malignancy (HM), whether they have undergone hematopoietic stem cell transplantation (HSCT) or not. ^1-3^ CARV can severely compromise the effectiveness of anti-cancer treatments, making them one of the most challenging complications in HM patients. ^2,4^ In recent years, there has been a growing understanding of the importance and the management of CARV in HM. A rising interest is reflected in the increasing body of scientific literature exploring the role and impact of CARV, including consensus guidelines. ^5-7^ However, large-scale data from cooperative registries, which are crucial for developing real-world, evidence-based strategies, remain scarce. Such data could be invaluable to the haematology community, providing critical insights into the epidemiology, risk factors, and clinical outcomes of CARV in HM patients. With this knowledge, clinicians could more effectively mitigate the adverse effects of these infections on the overall treatment process.

The EPICOVIDEHA registry, ^8^ established in 2021, has been instrumental in addressing this gap by collecting extensive data on severe acute respiratory syndrome coronavirus 2 (SARS-CoV-2) infections in HM patients. The insights from EPICOVIDEHA registry have led to numerous scientific publications that have been vital in guiding clinicians throughout the coronavirus disease 2019 (COVID-19) pandemic, helping to develop preventive strategies to protect HM patients. ^9-29^ Building on the success of the SARS-CoV-2-dedicated data collection, the EPICOVIDEHA registry expanded its scope in 2023 to include other CARV and was renamed as EPICOVIDEHA-EPIFLUEHA. ^30^ This expansion aimed to provide a more comprehensive understanding of the epidemiology, risk factors, and clinical outcomes associated with CARV in HM patients.

In this manuscript, we present and analyse CARV cases in HM patients registered during the autumn-winter seasons of 2023-2024. Our goal is to contribute valuable data that can guide future strategies in the ongoing effort to improve care for HM patients facing the threat of respiratory viral infections.

**METHODS**

*Patients*

A total of 61 institutions from 24 countries (Supplementary figure 1) contributed data on CARV cases diagnosed between September 1, 2023, and March 31, 2024, in HM patients to the EPICOVIDEHA-EPIFLUEHA registry, ^8,30^ accessible at www.clinicalsurveys.net (EFS, TIVIAN, Cologne, Germany). EPICOVIDEHA-EPIFLUEHA, identified by National Clinical Trials Identifier NCT04733729, is an international, web-based registry focused on HM adult patients who contract CARV. It was established by the European Hematology Association Specialized Working Group (EHA-SWG) Infections in Hematology.

Patients were eligible if they had an active HM within the last five years immediately before the CARV diagnosis, were 18 years or older, had a laboratory-confirmed CARV, and received their diagnosis between September 1, 2023, and March 31, 2024. The period from September 2023 to March 2024 was chosen as it aligns with the peak season for CARV infections in the Northern hemisphere, ^31^ enabling a representative analysis in terms of comparability and impact on healthcare systems. Excluded were patients with solid tumours, non-malignant haematological disorders, age <18 years, those off-therapy or cured for more than five years before their respiratory viral infection, or those diagnosed solely via imaging. No restrictions were imposed concerning the type of pathogenic virus.

For each patient, data collected included baseline conditions prior to the CARV, such as age, biological sex, and HM status at diagnosis. Information on HM management (status and type of last treatment before infection), CARV diagnosis and symptomatology, prophylaxis and treatments (including vaccines against influenza, respiratory syncytial virus (RSV), and SARS-CoV-2 administered within the year preceding the onset of infection), stay during infection, and outcomes (mortality and last follow-up date) were recorded. Patients who received an influenza, RSV, or SARS-CoV-2 vaccination more than one year prior to their CARV infection diagnosis were classified as "non-vaccinated" for the purposes of this analysis. The status of HM at infection onset was classified as active (onset, refractory and stable disease) or controlled (complete response). The severity of respiratory viral infection episodes was categorized as asymptomatic, mild, severe, or critical, as in previous EPICOVIDEHA-EPIFLUEHA publications. ^22,23,25^ To ensure data accuracy and completeness, a validation process was conducted by experts in haematology and infectious diseases. Contributors were contacted to resolve any pending queries, which helped maintain the integrity and reliability of the registry data. Missing data for variables included in regression analyses led to exclusion from the analysis.

*Objectives*

The primary objective was to examine the epidemiology and outcomes of HM patients affected by respiratory viruses during the specified period. Secondary objectives included determining the relative frequency of disease severity, intensive care unit (ICU) admissions, overall case-fatality rate, the impact of cancer treatment phases on outcomes, the effect of vaccine doses on outcomes, and the impact of treatment strategies for respiratory viral infections.

*Statistical analysis*

As an exploratory study, no a priori sample size calculation was performed. Data were summarized using frequencies and percentages for categorical variables and median, interquartile range (IQR), and absolute range for continuous variables. A univariable Cox regression model analysed factors influencing mortality in HM patients with respiratory viral infections. Clinically relevant variables were considered for multivariable analysis, which was conducted using the Wald backward method. Variables were included in the multivariable Cox regression model based on a statistical significance threshold (p ≤ 0.05). Mortality per viral pathogen was analysed using Kaplan-Meier survival plots, with survival probabilities compared via log-rank test. The Cox proportional hazards model was used to analyse variables impacting mortality, including biological sex, age, vaccination status at infection onset, infecting viral pathogen, comorbidities, neutrophil and lymphocyte counts, baseline HM, HM status at infection diagnosis, last chemotherapy strategy, symptoms at infection onset, treatment strategies, hospitalization during the infection episode, and secondary infections (bacterial, fungal, or other viral). Hazard Ratios (HR) and 95% confidence intervals (CI) were reported to quantify associations, with p ≤ 0.05 considered statistically significant. Statistical analyses were performed using SPSS version 25.0 (SPSS, IBM Corp, Chicago, IL, USA).

*Ethics statement*

The registry received ethical approval from the local ethics committee of the Fondazione Policlinico Universitario Agostino Gemelli, IRCCS, Università Cattolica del Sacro Cuore in Rome, Italy (Study ID: 3226), as well as from the respective ethics committees of participating institutions when required. The registry was further amended at the end of 2023 and named EPICOVIDEHA-EPIFLUEHA (Study ID: 113/23 del 03/01/2023). The anonymized data that do not contain any personally identifiable information from any sources implies that the informed consent is not applicable.

*Role of the funding source*

This research did not receive any funding. JSG, FM, LP, and OAC had access to and verified all raw data sets and decided to submit the manuscript. The corresponding author can provide the data supporting the findings of this study upon a reasonable request.

**RESULTS**

During the boreal fall-winter of 2023-2024, EPICOVIDEHA-EPIFLUEHA registered 1,312 patients from 24 countries (~~Figure 2,~~ Supplementary figures 1, 2, and 3). Among them, 48.7% (n=639/1,312) were diagnosed with SARS-CoV-2, 19.1% (n=250/1,312) with influenza, 12.3% (n=135/1,312) with RSV, and 9.8% (n=128/1,312) with rhinovirus. There were also cases of infections from parainfluenza (2.4%, n=31/1,312), metapneumovirus (2.1%, n=28/1,312), non-SARS-CoV-2 coronaviruses (1.5%, n=20/1,312), and enterovirus/rhinovirus (1.4%, n=18/1,312). Additionally, 4.5% (n=59/1,312) had multiple viral infections, mostly involving influenza (44.1%, n=26/59) or SARS-CoV-2 (57.6%, n=34/59). Of these, 93.2% (n=55/59) had two viruses, while 6.8% (n=4/59) had three. The most common coinfections were influenza plus SARS-CoV-2 (20.3%, n=12/59) and RSV plus SARS-CoV-2 (18.6%, n=11/59) (Table 1).

Among the patients documented, 54.8% (n=719/1,312) were male. The highest male-to-female ratios were seen in cases of influenza (58.8%, n=147/250) and enterovirus/rhinovirus infections (61.1%, n=11/18). The median age of the patients was 65 years (IQR 54-73, range of 18-96). The youngest patients were those with adenovirus or bocavirus infections, with a median age of 44 years (IQR 36-59, range 32-70), while the oldest were those with SARS-CoV-2 infections, with a median age of 66 years (IQR 57-73, range 19-95). Regarding viral infections with an available vaccination schedule, limited to influenza H1N1 and SARS-CoV-2, only 5.3% (70 out of 1,312) of the patients had been vaccinated against the respective virus., with the vaccination occurring a median of 79 days before the diagnosis of the infection (IQR 54-125, range 0-346). About 28.9% (n=379/1,312) of patients had two or more comorbidities. The most common underlying conditions were chronic heart disease (42.5%, n=558/1,312), chronic lung disease (13.6%, n=179/1,312), diabetes mellitus (13.6%, n=178/1,312), and a history of smoking (12.4%, n=79/1,312). Chronic heart disease was the most prevalent underlying condition regardless of the viral infection, although other major conditions did vary depending on the virus. Neutropenia (fewer than 500 neutrophils/mm³) was observed in 9.8% (n=128/1,312) of patients, and lymphopenia (200 or fewer lymphocytes/mm³) was seen in 10.9% (n=143/1,312) (Table 1, Supplementary table 1, Supplementary figure ~~2~~4).

Lymphomas were the most common underlying HM, affecting 30.6% (n=401/1,312) of the patients, with 27.6% (n=362/1,312) having non-Hodgkin lymphoma. Other significant malignancies included plasma cell malignancies (22.3%, n=293/1,312) and acute myeloid leukaemia (19.7%, n=259/1,312). Among influenza patients, plasma cell malignancies were the most common (28.0%, n=70/250), whereas for metapneumovirus infections, lymphomas and plasma cell malignancies were equally prevalent (28.6%, n=8/28 each). At the time of diagnosis of the viral infection, 49.6% (n=651/1,312) of the patients had a controlled underlying malignancy, while 50.4% (n=661/1,312) had an active malignancy. Two-thirds of the patients (63.7%, n=836/1,312) had undergone drug-based chemotherapy in the three months prior to their infection diagnosis, and 8.3% (n=109/1,312) had received either chimeric antigen receptor T-cell (CAR-T) therapy or an HSCT in the prior six months. Most viral infections were diagnosed between November 2023 (16.5%, n=216/1,312) and January 2024 (21.3%, n=280/1,312), with a peak in December 2023 (24.5%, n=321/1,312). SARS-CoV-2 was the most prevalent pathogen from September to December 2023 (60.1% to 71.5% of all infections in the period), while influenza was more common from January to March 2024 (20.5% to 40.4% of all infections in the period). RSV infections were high in February 2024 (24.6%, n=43/175), and metapneumovirus infections peaked in March 2024 (18.1%, n=15/83) (Table 1, Figure ~~2~~1~~figures 2 and 3~~, Supplementary table 1, Supplementary figure 5).

Most patients experienced either asymptomatic (9.6%, n=126/1,312) or mild infections (66.5%, n=872/1,312). Critical infections were reported in 7.9% (n=103/1,312) of cases, with higher rates seen in parainfluenza (16.1%, n=5/31), metapneumovirus (14.3%, n=4/28), and influenza infections (12.0%, n=30/250). A total of 56.3% (n=739/1,312) of patients required hospital admission, with the highest rate among those with RSV infections (69.6%, n=94/135). A total of 103/1,312 patients (7.9%) were admitted to the ICU, with 43/103 (41.7%) requiring mechanical ventilation. The highest ICU admission rates were seen in patients with parainfluenza (16.1%, n=5/31) and metapneumovirus (14.3%, n=4/22) (Supplementary table 1). No treatment was given to 39% (n=512/1,312) of patients. Among those who did receive treatment, the most common were antivirals, with or without corticosteroids (53.7%, n=704/1,312), especially for influenza (78.4%, n=196/250) and SARS-CoV-2 infections (70.3%, n=449/639). Secondary infections occurred in 18.4% (n=242/1,312) of patients, with bacterial infections being the most common (13.5%, n=177/1,312). The highest rates of secondary bacterial infections were observed in enterovirus/rhinovirus co-infections (38.9%, n=7/18), rhinovirus infections (21.1%, n=27/128), and RSV infections (20.0%, n=27/135). Fungal and other viral secondary infections were less common overall (4%, n=53/1,312 each), though they were relatively more frequent in parainfluenza (9.7%, n=3/31) and metapneumovirus infections (10.7%, n=3/28) (Table 1, Supplementary table 1).

The overall 30-day mortality rate was 5.9% (77/1,312). Parainfluenza infections had the highest all-cause mortality rate at 19.4% (6/31), surpassing other common CARV such as influenza (8.8%, 22/250) and SARS-CoV-2 (5.0%, 32/639). The highest CARV-attributable mortality rates were observed in influenza at 81.8% (18/22) and SARS-CoV-2 at 63.6% (49/77). Additionally, the progression of the underlying malignancy contributed to 64.9% (50/77) of the total deaths (Table 1, Supplementary table 1, Supplementary figure ~~3~~6).

Moreover, a pool of patients with the most prevalent pathogenic viruses was established, including cases of monoinfection from SARS-CoV-2, influenza, RSV, rhinovirus, parainfluenza, and metapneumovirus. The following factors were associated with increased mortality in the multivariable analysis (Table 2): parainfluenza infection (p=0.040, adjusted HR [aHR] 3.326, 95% CI 1.058 - 10.453), baseline smoking history (p=0.028, aHR 3.867, 95% CI 1.156 - 12.930), secondary bacterial infection (p<0.001, aHR 4.023, 95% CI 2.110 - 7.673), and hospital admission, regardless whether to a normal ward (p=0.018, aHR 11.683, 95% CI 1.535 - 88.929) or to an ICU (p<0.001, aHR 49.946, 95% CI 6.462 - 386.020). Conversely, reduced mortality was associated with the absence of lymphopenia (201-499 lymphocytes/mm³, p<0.001, aHR 0.173, 95% CI 0.063 - 0.481; 500-999 lymphocytes/mm³, p=0.002, aHR 0.381, 95% CI 0.206 - 0.704) (Table 2). Furthermore, Kaplan-Meier survival plots indicated significantly higher survival probabilities in SARS-CoV-2 patients compared to those with influenza (p=0.006) or parainfluenza (p<0.001), also a higher survival probability in rhinovirus patients compared to those with influenza (p=0.009) and parainfluenza (p<0.001), and in RSV patients compared to those with parainfluenza (p=0.048) (Figure ~~5~~2).

Sensitivity analyses were conducted to identify factors associated with increased mortality in patients with SARS-CoV-2, influenza, and RSV infections, respectively. For SARS-CoV-2 infections, Cox multivariable regression analysis identified myelodysplastic syndrome (p=0.005, aHR 6.102, 95% CI 1.705 - 21.842), active malignancy (p=0.036, aHR 3.884, 95% CI 1.093 - 13.519), fungal secondary infection (p=0.037, aHR 3.761, 95% CI 1.084 - 13.051), and hospital admission, either in a non-ICU (p=0.014, aHR 13.319, 95% CI 1.704 - 104.137) or ICU ward (p=0.001, aHR 39.351, 95% CI 4.384 - 353.185), as factors associated with increased mortality. Conversely, the absence of lymphopenia was a protective factor (201-499 lymphocytes/mm³, p=0.011 aHR 0.177, 95% CI 0.047 - 0.672; 500-999 lymphocytes/mm³, p=0.001, aHR 0.170, 95% CI 0.059 - 0.492). In influenza patients, secondary bacterial infection was associated with increased mortality in the multivariable analysis (p<0.001, aHR 10.837, 95% CI 4.085 - 28.750), while the absence of lymphopenia was protective (201-499 lymphocytes/mm³, p=0.008, aHR 0.058, 95% CI 0.007 - 0.479; 500-999 lymphocytes/mm³, p=0.006, aHR 0.260, 95% CI 0.099 - 0.684). For RSV infections, multivariable analysis indicated that secondary bacterial (p=0.006, aHR 9.830, 95% CI 1.902 - 50.815) and viral (p=0.031, aHR 6.298, 95% CI 1.182 - 33.551) infections were associated with an increased risk of mortality (Supplementary tables 2, 3, and 4).

**DISCUSSION**

Our study highlights the major burden of CARV infections in HM patients, with SARS-CoV-2, influenza, and RSV being the most prevalent. Increased mortality has been seen to be linked to active HM, secondary bacterial infections, and ICU admission, while the absence of lymphopenia offers protection. The findings reveal that the impact of these infections varied by CARV, underscoring the need for tailored management strategies. This stresses the importance of adapting treatment approaches and maintaining vigilant monitoring to address the evolving nature of CARV infections in HM patients.

Pathogen distribution and seasonal peaks align with known CARV circulation trends in the Northern hemisphere. ^2,3,5-7,32^ From September to December 2023, SARS-CoV-2 was the most prevalent virus in our patients, likely driven by highly transmissible variants, ^33^ potentially insufficient vaccine coverage, ^34,35^ and increased social activities and gatherings during the holidays. ^36^ Of note, SARS-CoV-2 infection levels have been described to be less affected by environmental temperature changes. ^37^ Conversely, influenza increased from January to March 2024, reflecting its typical seasonal rise ^38^ and reclaiming prominence after recent years where SARS-CoV-2 was the dominant CARV. ^39,40^ This shift may be linked to the relaxing of COVID-19-related precautions that had previously kept influenza rates low. RSV and metapneumovirus peaked in February and March 2024, respectively, while rhinovirus remained consistently present but at low levels throughout the study. This multi-pathogen scenario highlights the need for comprehensive preventive measures and increased vigilance, particularly for high-risk groups such as patients with HM.

The study found a limited vaccination rate for influenza, RSV, and SARS-CoV-2 – CARV with currently available vaccines – ^41^ highlighting a major gap in preventive care. This is especially concerning for HM patients, who are at higher risk for severe infection, as compared to the general population. In parallel, the median time between vaccination and infection was 79 days, which could suggest a waning immunity over time. To overcome the situation, strategies like more frequent boosters ^42^ or passive immunization might be necessary. ^18^ Besides, understanding the reasons for low vaccination rates – such as safety concerns, ^43^ scheduling conflicts with antineoplastic treatment, ^44^ lack of awareness, ^45^ or vaccine hesitancy ^46^ ^47^ – is also crucial. Improving education, refining vaccination protocols, and monitoring immune responses could help increase vaccination rates and better protect these patients. ^48^

Our study cohort was characterized by a high prevalence of lymphomas, particularly non-Hodgkin lymphoma, followed by plasma cell malignancies and acute myeloid leukaemia. This distribution matches patterns seen previously in CARV. ^2,23^ Interestingly, we found no significant differences in 30-day mortality based on malignancy treatment type, contrasting with some previous reports. ^6,32,49,50^ Most of our patients had received drug-based chemotherapy in the past three months, indicating uniformly high immunosuppression. Although recent allogeneic HSCT is linked to higher CARV mortality in the literature, ^6,32,49-51^ our study's broader timeframe (six months) may account for this discrepancy, which also exists in the literature, with reports describing the lack of correlation between HSCT and increased CARV-related mortality. ^52,53^ Despite this, the high rate of active HM causing severe immune dysregulation, so as recent treatments likely contributed to increased vulnerability to viral infections, aligning with existing literature. ^54^

In addition to HM, a large number of patients had other underlying conditions that increased their risk of severe CARV infections. Chronic heart disease was the most common, followed by chronic lung disease, diabetes, and a history of smoking. These conditions weaken the immune system and complicate CARV infection management. Chronic heart disease was notably prevalent across all viral infections studied. Multiple comorbidities complicate treatment, possibly requiring specific antiviral medications or changes to malignancy treatment regimens. This highlights the need for a multidisciplinary approach involving not only infectious diseases and haematology but also cardiology, pulmonology, or endocrinology. Lymphopenia and prior corticosteroid use are also known to worsen outcomes in patients with CARV and HM. ^6,7,55^ Our study found that severe lymphopenia significantly increased mortality, but we did not collect data on corticosteroid use, limiting our analysis. Notably, smoking history emerged as a significant risk factor for mortality, a finding not widely reported. While smoking has been linked to severe RSV infections in HSCT patients, ^56^ this was not observed in our study. Additionally, we did not find a significant link between neutropenia and mortality, despite prior research suggesting it correlates with RSV progression to lower respiratory tract infections. ^56^

Most patients had asymptomatic or mild infections, yet nearly 10% of the total required ICU admission, and half of those needed mechanical ventilation. Infection severity varied by pathogen, with influenza, parainfluenza, and metapneumovirus associated with higher rates of severe infection and ICU stays, in line with previous research. ^52,53,55,57-59^ This variation could stem from virus pathogenicity, the level of patient immunosuppression, or existing underlying comorbidities. Notably, 39% of patients did not receive antiviral treatment, likely due to the mild nature of the infections, the lack of effective treatments, ^2^ or concerns about drug interactions and toxicity. ^2^ Antiviral therapy, often combined with corticosteroids, was common for influenza and SARS-CoV-2, where effective options exist, ^25^ improving management, outcomes, and burden to the healthcare system. The lack of treatment options for viruses like metapneumovirus, parainfluenza or RSV highlight the urgent need for improved antiviral prophylaxis and therapies for CARV infections, particularly in HM patients.

Secondary infections occurred in one in five patients, with bacterial infections being the most common, especially in those with rhinovirus and RSV infections. Bacterial co-infections complicate viral infections in HM patients, increasing morbidity and mortality. ^54,55,60-63^ The high rate of bacterial infections indicates that viral infections often impair mucosal barriers and immune responses, leading to bacterial overgrowth. In our study, bacterial secondary infections were linked to a four-fold increase in mortality risk, consistent with another research. ^54,55,64^ While less common, the risk of secondary fungal and viral infections highlights the severe immune suppression in this group, particularly after recent treatments like chemotherapy, HSCT, or CAR-T therapy. Managing these infections requires a careful balance of timely antimicrobial treatment and resistance prevention.

The overall 30-day mortality rate was 6%, with significant variation among viruses. Both, the overall and attributable mortality rates were highest for parainfluenza, followed by influenza and metapneumovirus. The high mortality with parainfluenza, despite its lower prevalence, highlights the need for better antiviral options and the high vulnerability of HM patients. Key mortality risk factors included parainfluenza infection, smoking history, secondary bacterial infections, and hospital admission, particularly to the ICU, all of which have been widely reported in the literature. ^2,3,6,7,32,54-56,65,66^ Conversely, the absence of lymphopenia was linked to lower mortality, suggesting that maintaining lymphocyte counts may be protective. Progression of the underlying malignancy was responsible for about 65% of deaths, but our focus was on overall mortality, as attributable mortality can be influenced by subjective clinical judgment.

Our registry study has several notable limitations. First, the study design did not allow us to calculate the incidence of CARV by HM type, as we lacked the necessary denominator, hindering our understanding of type-specific CARV rates. Second, there may be an underestimation of CARV cases due to a likely bias toward documenting more severe infections, which could lead to overestimating their severity. Third, with only about 5% of patients vaccinated, ~~we could not reliably assess vaccine effectiveness in preventing severe disease or altering infection outcomes~~, our study was limited in assessing vaccine effectiveness for preventing severe disease or modifying infection outcomes. This finding underscores the urgent need to enhance vaccination efforts among HM patients and their close contacts—including family members and healthcare workers—especially given the seasonal peaks of these respiratory infections. The cyclical pattern and respiratory transmission of CARVs like SARS-CoV-2 and influenza indicate that coordinating vaccinations with peak viral circulation periods could offer greater protection. The low vaccination rate reported in our cohort may partly result from underreporting in medical records but also highlights a preventive gap that could leave many patients vulnerable. Expanding vaccine coverage and providing timely boosters when available could strengthen immunity during high-risk periods, reducing the impact of severe CARV infections in this high-risk group. Fourth, the observational nature of the study introduces potential selection and reporting biases. Additionally, differences in healthcare access and prescription practices across the participating countries complicate the analysis of CARV treatments, particularly given the lack of standardized protocols for managing these infections. Furthermore, the study did not include detailed data on specific viral strains or variants, which could influence the outcomes, especially for SARS-CoV-2 and influenza.

In conclusion, our research shows how CARV pose a significant risk to HM patients, with elevated mortality linked to active malignancies, secondary bacterial infections, ICU admissions, and lymphopenia. The study observed seasonal peaks, with SARS-CoV-2 dominating late 2023 and influenza in early 2024, emphasizing the need for seasonally adjusted preventive strategies. Low vaccination rates among HM patients are concerning, highlighting the need for improved vaccine strategies. High comorbidity prevalence, particularly chronic heart and lung diseases, necessitates a multidisciplinary care approach. Severe CARV cases often required ICU care, underscoring the urgent need for better antiviral treatments, particularly for parainfluenza and metapneumovirus, while secondary bacterial infections significantly increased mortality risk.

***Contributors***

JSG, FM, LP and OAC contributed to the study design and study supervision. JSG did the statistical plan and analysis. JSG, FM, and LP interpreted the data and wrote the paper. All the authors recruited, and documented participants, critically read, reviewed, and agreed to publish the manuscript.

***Data sharing statement***

The corresponding author can provide the data supporting the findings of this study upon a reasonable request.

***Declaration of interests***

Authors declare no competing interest related to the submitted work.

All authors had full access to the data and had final responsibility for the decision to submit for publication.

***Acknowledgments***

We would like to express our deepest gratitude to everyone who contributed to the development of this manuscript. In particular, we wish to pay special tribute to Dr Alberto López-García. His dedication, knowledge, and unwavering support have been instrumental in advancing the EPICOVIDEHA-EPIFLUEHA research since its inception. His legacy will endure through this work, and he will always be remembered with great appreciation and respect.

**REFERENCES**

1. Martino R, Ramila E, Rabella N, et al. Respiratory virus infections in adults with hematologic malignancies: a prospective study. *Clin Infect Dis* 2003; **36**(1): 1-8.

2. Gabutti G, De Motoli F, Sandri F, Toffoletto MV, Stefanati A. Viral Respiratory Infections in Hematological Patients. *Infect Dis Ther* 2020; **9**(3): 495-510.

3. Fontana L, Strasfeld L. Respiratory Virus Infections of the Stem Cell Transplant Recipient and the Hematologic Malignancy Patient. *Infect Dis Clin North Am* 2019; **33**(2): 523-44.

4. Popescu CM, Ursache AL, Feketea G, et al. Are Community Acquired Respiratory Viral Infections an Underestimated Burden in Hematology Patients? *Microorganisms* 2019; **7**(11).

5. von Lilienfeld-Toal M, Berger A, Christopeit M, et al. Community acquired respiratory virus infections in cancer patients-Guideline on diagnosis and management by the Infectious Diseases Working Party of the German Society for haematology and Medical Oncology. *Eur J Cancer* 2016; **67**: 200-12.

6. Hirsch HH, Martino R, Ward KN, Boeckh M, Einsele H, Ljungman P. Fourth European Conference on Infections in Leukaemia (ECIL-4): guidelines for diagnosis and treatment of human respiratory syncytial virus, parainfluenza virus, metapneumovirus, rhinovirus, and coronavirus. *Clin Infect Dis* 2013; **56**(2): 258-66.

7. Engelhard D, Mohty B, de la Camara R, Cordonnier C, Ljungman P. European guidelines for prevention and management of influenza in hematopoietic stem cell transplantation and leukemia patients: summary of ECIL-4 (2011), on behalf of ECIL, a joint venture of EBMT, EORTC, ICHS, and ELN. *Transpl Infect Dis* 2013; **15**(3): 219-32.

8. Salmanton-Garcia J, Busca A, Cornely OA, et al. EPICOVIDEHA: A Ready to Use Platform for Epidemiological Studies in Hematological Patients With COVID-19. *Hemasphere* 2021; **5**(7): e612.

9. Aiello TF, Salmanton-Garcia J, Marchesi F, et al. Dexamethasone treatment for COVID-19 is related to increased mortality in hematologic malignancy patients: results from the EPICOVIDEHA registry. *Haematologica* 2024; **109**(8): 2693-700.

10. Blennow O, Salmanton-Garcia J, Nowak P, et al. Outcome of infection with omicron SARS-CoV-2 variant in patients with hematological malignancies: An EPICOVIDEHA survey report. *Am J Hematol* 2022; **97**(8): E312-E7.

11. Busca A, Salmanton-Garcia J, Marchesi F, et al. Outcome of COVID-19 in allogeneic stem cell transplant recipients: Results from the EPICOVIDEHA registry. *Front Immunol* 2023; **14**: 1125030.

12. Cattaneo C, Salmanton-Garcia J, Marchesi F, et al. Simultaneous Onset of Haematological Malignancy and COVID: An Epicovideha Survey. *Cancers (Basel)* 2022; **14**(22).

13. Criscuolo M, Salmanton-Garcia J, Fracchiolla N, et al. SARS-CoV-2 Infection In Patients With Mastocytosis: An EPICOVIDEHA Report. *J Investig Allergol Clin Immunol* 2023; **33**(3): 225-7.

14. El-Ashwah S, Salmanton-Garcia J, Bilgin YM, et al. The mortality of COVID-19 in CML patients from 2020 until 2022: results from the EPICOVIDEHA survey. *Leuk Lymphoma* 2024; **65**(2): 199-208.

15. Infante MS, Salmanton-Garcia J, Fernandez-Cruz A, et al. B-cell malignancies treated with targeted drugs and SARS-CoV-2 infection: A European Hematology Association Survey (EPICOVIDEHA). *Front Oncol* 2022; **12**: 992137.

16. Lahmer T, Salmanton-Garcia J, Marchesi F, et al. Need for ICU and outcome of critically ill patients with COVID-19 and haematological malignancies: results from the EPICOVIDEHA survey. *Infection* 2024; **52**(3): 1125-41.

17. Lamure S, Salmanton-Garcia J, Robin-Marieton E, et al. COVID-19 and hairy-cell leukemia: an EPICOVIDEHA survey. *Blood Adv* 2022; **6**(13): 3870-4.

18. Marchesi F, Salmanton-Garcia J, Buquicchio C, et al. Passive pre-exposure immunization by tixagevimab/cilgavimab in patients with hematological malignancy and COVID-19: matched-paired analysis in the EPICOVIDEHA registry. *J Hematol Oncol* 2023; **16**(1): 32.

19. Marchesi F, Salmanton-Garcia J, Emarah Z, et al. COVID-19 in adult acute myeloid leukemia patients: a long-term follow-up study from the European Hematology Association survey (EPICOVIDEHA). *Haematologica* 2023; **108**(1): 22-33.

20. Marchetti M, Salmanton-Garcia J, El-Ashwah S, et al. Outcomes of SARS-CoV-2 infection in Ph-neg chronic myeloproliferative neoplasms: results from the EPICOVIDEHA registry. *Ther Adv Hematol* 2023; **14**: 20406207231154706.

21. Musto P, Salmanton-Garcia J, Sgherza N, et al. Survival in multiple myeloma and SARS-COV-2 infection through the COVID-19 pandemic: Results from the EPICOVIDEHA registry. *Hematol Oncol* 2024; **42**(1): e3240.

22. Pagano L, Salmanton-Garcia J, Marchesi F, et al. Breakthrough COVID-19 in vaccinated patients with hematologic malignancies: results from the EPICOVIDEHA survey. *Blood* 2022; **140**(26): 2773-87.

23. Pagano L, Salmanton-Garcia J, Marchesi F, et al. COVID-19 infection in adult patients with hematological malignancies: a European Hematology Association Survey (EPICOVIDEHA). *J Hematol Oncol* 2021; **14**(1): 168.

24. Rossi G, Salmanton-Garcia J, Cattaneo C, et al. Age, successive waves, immunization, and mortality in elderly COVID-19 hematological patients: EPICOVIDEHA findings. *Int J Infect Dis* 2023; **137**: 98-110.

25. Salmanton-Garcia J, Marchesi F, Farina F, et al. Decoding the historical tale: COVID-19 impact on haematological malignancy patients-EPICOVIDEHA insights from 2020 to 2022. *EClinicalMedicine* 2024; **71**: 102553.

26. Salmanton-Garcia J, Marchesi F, Glenthoj A, et al. Improved Clinical Outcome of COVID-19 in Hematologic Malignancy Patients Receiving a Fourth Dose of Anti-SARS-CoV-2 Vaccine: An EPICOVIDEHA Report. *Hemasphere* 2022; **6**(11): e789.

27. Salmanton-Garcia J, Marchesi F, Gomes da Silva M, et al. Nirmatrelvir/ritonavir in COVID-19 patients with haematological malignancies: a report from the EPICOVIDEHA registry. *EClinicalMedicine* 2023; **58**: 101939.

28. Salmanton-Garcia J, Marchesi F, Koehler P, et al. Molnupiravir compared to nirmatrelvir/ritonavir for COVID-19 in high-risk patients with haematological malignancy in Europe. A matched-paired analysis from the EPICOVIDEHA registry. *Int J Antimicrob Agents* 2023; **62**(4): 106952.

29. van Doesum JA, Salmanton-Garcia J, Marchesi F, et al. Impact of SARS-CoV-2 vaccination and monoclonal antibodies on outcome post-CD19-directed CAR T-cell therapy: an EPICOVIDEHA survey. *Blood Adv* 2023; **7**(11): 2645-55.

30. Salmanton-Garcia J, Marchesi F, Itri F, et al. Unveiling the Hidden Burden: From EPICOVIDEHA to EPIFLUEHA, Exploring the Epidemiology of Respiratory Viral Infections in Hematological Patients. *Hemasphere* 2023; **7**(11): e970.

31. Garcia-Arroyo L, Prim N, Del Cuerpo M, et al. Prevalence and seasonality of viral respiratory infections in a temperate climate region: A 24-year study (1997-2020). *Influenza Other Respir Viruses* 2022; **16**(4): 756-66.

32. Atalla E, Kalligeros M, Mylona EK, et al. Impact of Influenza Infection Among Adult and Pediatric Populations With Hematologic Malignancy and Hematopoietic Stem Cell Transplant: A Systematic Review and Meta-Analysis. *Clin Ther* 2021; **43**(5): e66-e85.

33. Kaku Y, Okumura K, Padilla-Blanco M, et al. Virological characteristics of the SARS-CoV-2 JN.1 variant. *Lancet Infect Dis* 2024; **24**(2): e82.

34. Goldberg Y, Huppert A. To boost or not to boost: navigating post-pandemic COVID-19 vaccination. *Lancet Respir Med* 2023; **11**(12): 1039-41.

35. Lazarus JV, White TM, Wyka K, et al. Influence of COVID-19 on trust in routine immunization, health information sources and pandemic preparedness in 23 countries in 2023. *Nat Med* 2024; **30**(6): 1559-63.

36. Liu C, Huang J, Chen S, et al. The impact of crowd gatherings on the spread of COVID-19. *Environ Res* 2022; **213**: 113604.

37. Gur-Arie L, Stein M, Sefty H, et al. Hospital surveillance of respiratory viruses during the COVID-19 pandemic and beyond: contribution to the WHO mosaic framework, Israel, 2020 to 2023. *Euro Surveill* 2024; **29**(32).

38. Chemaly RF, Shah DP, Boeckh MJ. Management of respiratory viral infections in hematopoietic cell transplant recipients and patients with hematologic malignancies. *Clin Infect Dis* 2014; **59 Suppl 5**(Suppl 5): S344-51.

39. Lee SS, Viboud C, Petersen E. Understanding the rebound of influenza in the post COVID-19 pandemic period holds important clues for epidemiology and control. *Int J Infect Dis* 2022; **122**: 1002-4.

40. Pendrey CG, Strachan J, Peck H, et al. The re-emergence of influenza following the COVID-19 pandemic in Victoria, Australia, 2021 to 2022. *Euro Surveill* 2023; **28**(37).

41. CDC: Centers for Disease Control and Prevention. Immunization Recommendations for the 2023–2024 Respiratory Disease Season: At-A-Glance11/24/2023 CS-343797A INFLUENZA • COVID-19 • RSV. <https://www.cdc.gov/respiratory-viruses/tools-resources/downloads/respiratory-disease-at-a-glance-508.pdf> (Last accessed August 28, 2024). 2023.

42. Das Barshan A, Matsumoto-Takahashi ELA. Efficacy of COVID-19 Vaccines in Patients with Hematological Malignancy Compared to Healthy Controls: A Systematic Review and Meta-analysis. *JMA J* 2024; **7**(2): 153-71.

43. Lai CC, Chen IT, Chao CM, Lee PI, Ko WC, Hsueh PR. COVID-19 vaccines: concerns beyond protective efficacy and safety. *Expert Rev Vaccines* 2021; **20**(8): 1013-25.

44. Cesaro S, Mikulska M, Hirsch HH, et al. Update of recommendations for the management of COVID-19 in patients with haematological malignancies, haematopoietic cell transplantation and CAR T therapy, from the 2022 European Conference on Infections in Leukaemia (ECIL 9). *Leukemia* 2023; **37**(9): 1933-8.

45. La EM, Bunniran S, Garbinsky D, et al. Respiratory syncytial virus knowledge, attitudes, and perceptions among adults in the United States. *Hum Vaccin Immunother* 2024; **20**(1): 2303796.

46. Schumacher S, Salmanton-Garcia J, Liekweg A, et al. Increasing influenza vaccination coverage in healthcare workers: analysis of an intensified on-site vaccination campaign during the COVID-19 pandemic. *Infection* 2023; **51**(5): 1417-29.

47. Schumacher S, Salmanton-Garcia J, Cornely OA, Mellinghoff SC. Increasing influenza vaccination coverage in healthcare workers: a review on campaign strategies and their effect. *Infection* 2021; **49**(3): 387-99.

48. Cremer LM, Bethe B, Borchmann P, et al. Immunogenicity of COVID-19 vaccination in immunocompromised patients (Auto-COVID-VACC): Protocol for a multicenter prospective non-interventional study. *JMIR Research Protocols* 2024.

49. Mulroney CM, Abid MB, Bashey A, et al. Incidence and impact of community respiratory viral infections in post-transplant cyclophosphamide-based graft-versus-host disease prophylaxis and haploidentical stem cell transplantation. *Br J Haematol* 2021; **194**(1): 145-57.

50. Vakil E, Evans SE. Viral Pneumonia in Patients with Hematologic Malignancy or Hematopoietic Stem Cell Transplantation. *Clin Chest Med* 2017; **38**(1): 97-111.

51. Shah JN, Chemaly RF. Management of RSV infections in adult recipients of hematopoietic stem cell transplantation. *Blood* 2011; **117**(10): 2755-63.

52. Shah DP, Shah PK, Azzi JM, El Chaer F, Chemaly RF. Human metapneumovirus infections in hematopoietic cell transplant recipients and hematologic malignancy patients: A systematic review. *Cancer Lett* 2016; **379**(1): 100-6.

53. Shah DP, Shah PK, Azzi JM, Chemaly RF. Parainfluenza virus infections in hematopoietic cell transplant recipients and hematologic malignancy patients: A systematic review. *Cancer Lett* 2016; **370**(2): 358-64.

54. Unal S, Schnitzler P, Giesen N, Wedde M, Durrwald R, Tabatabai J. Molecular epidemiology and disease severity of influenza virus infection in patients with haematological disorders. *J Med Virol* 2023; **95**(6): e28835.

55. Tabatabai J, Schnitzler P, Prifert C, et al. Parainfluenza virus infections in patients with hematological malignancies or stem cell transplantation: Analysis of clinical characteristics, nosocomial transmission and viral shedding. *PLoS One* 2022; **17**(7): e0271756.

56. Khawaja F, Chemaly RF. Respiratory syncytial virus in hematopoietic cell transplant recipients and patients with hematologic malignancies. *Haematologica* 2019; **104**(7): 1322-31.

57. Lefeuvre C, Salmona M, Bondeelle L, et al. Frequent lower respiratory tract disease in hematological patients with parainfluenza virus type 3 infection. *J Med Virol* 2021; **93**(11): 6371-6.

58. Pochon C, Voigt S. Respiratory Virus Infections in Hematopoietic Cell Transplant Recipients. *Front Microbiol* 2018; **9**: 3294.

59. Hakim H, Dallas R, Zhou Y, et al. Acute respiratory infections in children and adolescents with acute lymphoblastic leukemia. *Cancer* 2016; **122**(5): 798-805.

60. Vanderbeke L, Spriet I, Breynaert C, Rijnders BJA, Verweij PE, Wauters J. Invasive pulmonary aspergillosis complicating severe influenza: epidemiology, diagnosis and treatment. *Curr Opin Infect Dis* 2018; **31**(6): 471-80.

61. Goka E, Vallely P, Mutton K, Klapper P. Influenza A viruses dual and multiple infections with other respiratory viruses and risk of hospitalisation and mortality. *Influenza Other Respir Viruses* 2013; **7**(6): 1079-87.

62. Garcia-Vidal C, Barba P, Arnan M, et al. Invasive aspergillosis complicating pandemic influenza A (H1N1) infection in severely immunocompromised patients. *Clin Infect Dis* 2011; **53**(6): e16-9.

63. Schauwvlieghe A, Rijnders BJA, Philips N, et al. Invasive aspergillosis in patients admitted to the intensive care unit with severe influenza: a retrospective cohort study. *Lancet Respir Med* 2018; **6**(10): 782-92.

64. Rachow T, Lamik T, Kalkreuth J, et al. Detection of community-acquired respiratory viruses in allogeneic stem-cell transplant recipients and controls-A prospective cohort study. *Transpl Infect Dis* 2020; **22**(6): e13415.

65. Sanli K, Ayer M, Alacam S, Gumus A, Karabulut N. Retrospective analysis of respiratory virus infections in adults with hematologic malignancies. *Eur Rev Med Pharmacol Sci* 2023; **27**(21): 10785-97.

66. Abbas S, Raybould JE, Sastry S, de la Cruz O. Respiratory viruses in transplant recipients: more than just a cold. Clinical syndromes and infection prevention principles. *Int J Infect Dis* 2017; **62**: 86-93.

**TABLES**

**Table 1.** Profile of EPICOVIDEHA-EPIFLUEHA patients during the Winter season: September 2023 - March 2024

|  | **Overall** | | **SARS-CoV-2** | | **Influenza** | | **RSV** | |
| --- | --- | --- | --- | --- | --- | --- | --- | --- |
|  | n=1312, 100.0% | | n=639, 48.7% | | n=250, 19.1% | | n=135, 10.3% | |
|  | n | % | n | % | n | % | n | % |
| **Sex** |  |  |  |  |  |  |  |  |
| Female | 593 | 45.2 | 304 | 47.6 | 103 | 41.2 | 59 | 43.7 |
| Male | 719 | 54.8 | 335 | 52.4 | 147 | 58.8 | 76 | 56.3 |
| **Age** | 65 (54-73) [18-96] | | 66 (57-73) [19-95] | | 64 (55-73) [18-93] | | 63 (53-71) [18-87] | |
| **Vaccination at infection onset** |  | |  | |  | |  | |
| Not vaccinated | 1242 | 94.7 | 617 | 96.6 | 208 | 83.2 | 134 | 99.3 |
| Influenza | 44 | 3.4 | 0 | 0.0 | 42 | 16.8 | 0 | 0.0 |
| RSV | 3 | 0.2 | 0 | 0.0 | 0 | 0.0 | 1 | 0.7 |
| SARS-CoV-2 | 23 | 1.8 | 22 | 3.4 | 0 | 0.0 | 0 | 0.0 |
| *Days from last vaccination to infection* | 79 (54-125) [0-346] | | 180 (31-319) [0-346] | | 73 (54-89) [0-344] | | 80 (80-80) [80-80] | |
| **Comorbidities** |  |  |  |  |  |  |  |  |
| 0-1 | 933 | 71.1 | 439 | 68.7 | 175 | 70.0 | 100 | 74.1 |
| 2+ | 379 | 28.9 | 200 | 31.3 | 75 | 30.0 | 35 | 25.9 |
| *Chronic cardiopathy* | 558 | 42.5 | 304 | 47.6 | 123 | 49.2 | 49 | 36.3 |
| *Chronic pulmonary disease* | 179 | 13.6 | 79 | 12.4 | 39 | 15.6 | 13 | 9.6 |
| *Diabetes mellitus* | 178 | 13.6 | 95 | 14.9 | 31 | 12.4 | 17 | 12.6 |
| *Liver disease* | 44 | 3.4 | 14 | 2.2 | 12 | 4.8 | 6 | 4.4 |
| *Obesity (BMI >30)* | 72 | 5.5 | 36 | 5.6 | 17 | 6.8 | 10 | 7.4 |
| *Renal impairment* | 89 | 6.8 | 52 | 8.1 | 14 | 5.6 | 6 | 4.4 |
| *Smoking history* | 151 | 11.5 | 79 | 12.4 | 34 | 13.6 | 15 | 11.1 |
| **Neutrophils** |  |  |  |  |  |  |  |  |
| < 500 | 128 | 9.8 | 50 | 7.8 | 23 | 9.2 | 23 | 17.0 |
| 500-999 | 101 | 7.7 | 40 | 6.3 | 25 | 10.0 | 15 | 11.1 |
| ≥ 1000 | 921 | 70.2 | 454 | 71.0 | 181 | 72.4 | 84 | 62.2 |
| **Lymphocytes** |  |  |  |  |  |  |  |  |
| ≤ 200 | 143 | 10.9 | 45 | 7.0 | 37 | 14.8 | 23 | 17.0 |
| 201-499 | 194 | 14.8 | 87 | 13.6 | 46 | 18.4 | 27 | 20.0 |
| ≥ 500 | 800 | 61.0 | 405 | 63.4 | 146 | 58.4 | 73 | 54.1 |
| **Baseline haematological malignancy** |  |  |  |  |  |  |  |  |
| Lymphoma | 401 | 30.6 | 214 | 33.5 | 59 | 23.6 | 41 | 30.4 |
| *Hodgkin lymphoma* | 39 | 3.0 | 14 | 2.2 | 6 | 2.4 | 5 | 3.7 |
| *Non-Hodgkin lymphoma* | 362 | 27.6 | 200 | 31.3 | 53 | 21.2 | 36 | 26.7 |
| Plasma cell malignancies | 293 | 22.3 | 140 | 21.9 | 70 | 28.0 | 31 | 23.0 |
| *Amyloid light-chain amyloidosis* | 7 | 0.5 | 5 | 0.8 | 0 | 0.0 | 0 | 0.0 |
| *Multiple myeloma* | 286 | 21.8 | 135 | 21.1 | 70 | 28.0 | 31 | 23.0 |
| Acute myeloid leukaemia | 259 | 19.7 | 107 | 16.7 | 57 | 22.8 | 31 | 23.0 |
| Acute lymphoblastic leukaemia | 84 | 6.4 | 32 | 5.0 | 11 | 4.4 | 14 | 10.4 |
| Chronic lymphocytic leukaemia | 121 | 9.2 | 78 | 12.2 | 17 | 6.8 | 10 | 7.4 |
| *Chronic lymphocytic leukaemia* | 113 | 8.6 | 75 | 11.7 | 15 | 6.0 | 9 | 6.7 |
| *Hairy cell leukaemia* | 8 | 0.6 | 3 | 0.5 | 2 | 0.8 | 1 | 0.7 |
| Myelodysplastic syndrome | 82 | 6.3 | 36 | 5.6 | 22 | 8.8 | 5 | 3.7 |
| Chronic myeloid malignancies | 59 | 4.5 | 26 | 4.1 | 12 | 4.8 | 2 | 1.5 |
| *Chronic myeloid leukaemia* | 24 | 1.8 | 11 | 1.7 | 3 | 1.2 | 0 | 0.0 |
| *Myelofibrosis* | 20 | 1.5 | 6 | 0.9 | 7 | 2.8 | 2 | 1.5 |
| *Essential thrombocythemia* | 7 | 0.5 | 5 | 0.8 | 0 | 0.0 | 0 | 0.0 |
| *Polycythaemia vera* | 6 | 0.5 | 2 | 0.3 | 2 | 0.8 | 0 | 0.0 |
| *Systemic mastocytosis* | 2 | 0.2 | 2 | 0.3 | 0 | 0.0 | 0 | 0.0 |
| Aplastic anaemia | 13 | 1.0 | 6 | 0.9 | 2 | 0.8 | 1 | 0.7 |
| **Status of baseline haematological malignancy** |  |  |  |  |  |  |  |  |
| Controlled malignancy | 651 | 49.6 | 311 | 48.7 | 119 | 47.6 | 67 | 49.6 |
| Active malignancy | 661 | 50.4 | 328 | 51.3 | 131 | 52.4 | 68 | 50.4 |
| **Last chemotherapy strategy before infection diagnosis** |  |  |  |  |  |  |  |  |
| Conventional chemotherapy | 238 | 18.1 | 101 | 15.8 | 48 | 19.2 | 32 | 23.7 |
| *< 3 months* | 197 | 15.0 | 84 | 13.1 | 42 | 16.8 | 26 | 19.3 |
| *> 3 months* | 41 | 3.1 | 17 | 2.7 | 6 | 2.4 | 6 | 4.4 |
| Demethylating agents | 86 | 6.6 | 36 | 5.6 | 29 | 11.6 | 8 | 5.9 |
| *< 3 months* | 78 | 5.9 | 32 | 5.0 | 27 | 10.8 | 8 | 5.9 |
| *> 3 months* | 8 | 0.6 | 4 | 0.6 | 2 | 0.8 | 0 | 0.0 |
| Immuno-chemotherapy | 468 | 35.7 | 272 | 42.6 | 81 | 32.4 | 39 | 28.9 |
| *< 3 months* | 389 | 29.6 | 231 | 36.2 | 67 | 26.8 | 31 | 23.0 |
| *> 3 months* | 79 | 6.0 | 41 | 6.4 | 14 | 5.6 | 8 | 5.9 |
| Targeted therapy | 188 | 14.3 | 101 | 15.8 | 42 | 16.8 | 19 | 14.1 |
| *< 3 months* | 172 | 13.1 | 96 | 15.0 | 35 | 14.0 | 18 | 13.3 |
| *> 3 months* | 16 | 1.2 | 5 | 0.8 | 7 | 2.8 | 1 | 0.7 |
| alloHSCT | 129 | 9.8 | 36 | 5.6 | 17 | 6.8 | 17 | 12.6 |
| *< 6 months* | 55 | 4.2 | 14 | 2.2 | 7 | 2.8 | 8 | 5.9 |
| *> 6 months* | 74 | 5.6 | 22 | 3.4 | 10 | 4.0 | 9 | 6.7 |
| autoHSCT | 47 | 3.6 | 16 | 2.5 | 8 | 3.2 | 6 | 4.4 |
| *< 6 months* | 43 | 3.3 | 14 | 2.2 | 7 | 2.8 | 6 | 4.4 |
| *> 6 months* | 4 | 0.3 | 2 | 0.3 | 1 | 0.4 | 0 | 0.0 |
| CAR-T | 15 | 1.1 | 5 | 0.8 | 2 | 0.8 | 2 | 1.5 |
| *< 6 months* | 11 | 0.8 | 3 | 0.5 | 1 | 0.4 | 2 | 1.5 |
| *> 6 months* | 4 | 0.3 | 2 | 0.3 | 1 | 0.4 | 0 | 0.0 |
| No treatment | 119 | 9.1 | 65 | 10.2 | 17 | 6.8 | 9 | 6.7 |
| Supportive measures | 23 | 1.8 | 7 | 1.1 | 7 | 2.8 | 3 | 2.2 |
| **Viral diagnosis month** |  |  |  |  |  |  |  |  |
| September 2023 | 86 | 6.6 | 56 | 8.8 | 0 | 0.0 | 0 | 0.0 |
| October 2023 | 151 | 11.5 | 108 | 16.9 | 4 | 1.6 | 6 | 4.4 |
| November 2023 | 216 | 16.5 | 151 | 23.6 | 7 | 2.8 | 13 | 9.6 |
| December 2023 | 321 | 24.5 | 193 | 30.2 | 49 | 19.6 | 35 | 25.9 |
| January 2024 | 280 | 21.3 | 94 | 14.7 | 113 | 45.2 | 27 | 20.0 |
| February 2024 | 175 | 13.3 | 27 | 4.2 | 60 | 24.0 | 43 | 31.9 |
| March 2024 | 83 | 6.3 | 10 | 1.6 | 17 | 6.8 | 11 | 8.1 |
| **Symptoms at viral infection onset** |  |  |  |  |  |  |  |  |
| No symptoms | 512 | 39.0 | 169 | 26.4 | 43 | 17.2 | 90 | 66.7 |
| Extrapulmonary symptoms | 704 | 53.7 | 449 | 70.3 | 196 | 78.4 | 20 | 14.8 |
| Pulmonary symptoms | 21 | 1.6 | 0 | 0.0 | 0 | 0.0 | 9 | 6.7 |
| **Viral infection severity** |  |  |  |  |  |  |  |  |
| Asymptomatic | 126 | 9.6 | 99 | 15.5 | 5 | 2.0 | 8 | 5.9 |
| Mild | 872 | 66.5 | 427 | 66.8 | 163 | 65.2 | 81 | 60.0 |
| Severe | 211 | 16.1 | 73 | 11.4 | 52 | 20.8 | 37 | 27.4 |
| Critical | 103 | 7.9 | 40 | 6.3 | 30 | 12.0 | 9 | 6.7 |
| **Viral infection treatment** |  |  |  |  |  |  |  |  |
| No treatment | 512 | 39.0 | 169 | 26.4 | 43 | 17.2 | 90 | 66.7 |
| Antivirals ± corticosteroids | 704 | 53.7 | 449 | 70.3 | 196 | 78.4 | 20 | 14.8 |
| Immunoglobulins | 21 | 1.6 | 0 | 0.0 | 0 | 0.0 | 9 | 6.7 |
| Corticosteroids | 65 | 5.0 | 15 | 2.3 | 9 | 3.6 | 14 | 10.4 |
| Immunoglobulins in combination | 10 | 0.8 | 6 | 0.9 | 2 | 0.8 | 2 | 1.5 |
| **Secondary infections** | 242 | 18.4 | 100 | 15.6 | 36 | 14.4 | 33 | 24.4 |
| Bacterial | 177 | 13.5 | 74 | 11.6 | 23 | 9.2 | 27 | 20.0 |
| Fungal | 53 | 4.0 | 20 | 3.1 | 12 | 4.8 | 8 | 5.9 |
| Other viral | 53 | 4.0 | 21 | 3.3 | 4 | 1.6 | 5 | 3.7 |
| **Patient stay during viral infection** |  |  |  |  |  |  |  |  |
| Home | 548 | 41.8 | 324 | 50.7 | 83 | 33.2 | 38 | 28.1 |
| Hospital | 739 | 56.3 | 306 | 47.9 | 162 | 64.8 | 94 | 69.6 |
| *Hospital, non-ICU* | 636 | 48.5 | 266 | 41.6 | 132 | 52.8 | 85 | 63.0 |
| *Hospital, ICU* | 103 | 7.9 | 40 | 6.3 | 30 | 12.0 | 9 | 6.7 |
| *Invasive MV* | 43 | 41.7 | 15 | 37.5 | 12 | 40.0 | 6 | 66.7 |
| *Non-invasive MV* | 34 | 33.0 | 9 | 22.5 | 13 | 43.3 | 3 | 33.3 |
| Not reported | 25 | 24.3 | 9 | 22.5 | 5 | 16.7 | 3 | 33.3 |
| **Mortality. d30** | 77 | 5.9 | 32 | 5.0 | 22 | 8.8 | 8 | 5.9 |
| Reason for mortality |  |  |  |  |  |  |  |  |
| *Haematological malignancy* | 50 | 3.8 | 23 | 3.6 | 12 | 4.8 | 6 | 4.4 |
| *Viral infection* | 49 | 3.7 | 23 | 3.6 | 18 | 7.2 | 2 | 1.5 |
| *Other reasons* | 36 | 2.7 | 12 | 1.9 | 9 | 3.6 | 5 | 3.7 |

alloHSCT, allogeneic hematopoietic stem cell transplantation; autoHSCT, autologous hematopoietic stem cell transplantation; BMI, body mass index; CAR-T, chimeric antigen receptor T-cell therapy; d30, day 30; ICU, intensive care unit; n, number; MV, mechanical ventilation; RSV, respiratory syncytial virus; SARS-CoV-2, severe acute respiratory syndrome coronavirus 2

**Table 2.** Factors associated with increased mortality in a pool of SARS-CoV-2, influenza, respiratory syncytial virus, rhinovirus, parainfluenza, and metapneumovirus

|  | **Univariable analysis** | | | | **Multivariable analysis** | | | |
| --- | --- | --- | --- | --- | --- | --- | --- | --- |
|  | **p** | **HR** | **95% CI** | | **p** | **HR** | **95% CI** | |
|  |  |  | **Lower** | **Upper** |  |  | **Lower** | **Upper** |
| **Sex** |  |  |  |  |  |  |  |  |
| Female | - | - | - | - | - | - | - | - |
| Male | 0.110 | 1.478 | 0.915 | 2.386 | - | - | - | - |
| **Age** | 0.053 | 1.017 | 1.000 | 1.034 | 0.196 | 1.013 | 0.993 | 1.033 |
| **Vaccination at infection onset** |  |  |  |  |  |  |  |  |
| Not vaccinated | - | - | - | - | - | - | - | - |
| Influenza | 0.258 | 1.790 | 0.653 | 4.909 | - | - | - | - |
| RSV | 0.971 | - | - | - | - | - | - | - |
| SARS-CoV-2 | 0.712 | 0.690 | 0.096 | 4.968 | - | - | - | - |
| **Viruses** |  |  |  |  |  |  |  |  |
| SARS-CoV-2 | - | - | - | - | - | - | - | - |
| Influenza | **0.006 *** | 2.138 | 1.241 | 3.681 | 0.062 | 1.985 | 0.966 | 4.077 |
| RSV | 0.312 | 1.492 | 0.687 | 3.240 | 0.595 | 0.770 | 0.294 | 2.019 |
| Rhinovirus | 0.232 | 0.486 | 0.149 | 1.587 | 0.162 | 0.343 | 0.077 | 1.538 |
| Parainfluenza | **0.001 *** | 4.234 | 1.770 | 10.130 | **0.040 *** | 3.326 | 1.058 | 10.453 |
| Metapneumovirus | 0.320 | 2.064 | 0.494 | 8.621 | 0.507 | 1.674 | 0.365 | 7.666 |
| **Comorbidities** |  |  |  |  |  |  |  |  |
| 0-1 | - | - | - | - | - | - | - | - |
| 2+ | 0.228 | 1.343 | 0.832 | 2.168 | - | - | - | - |
| *Chronic cardiopathy* | 0.303 | 0.786 | 0.497 | 1.243 | - | - | - | - |
| *Chronic pulmonary disease* | 0.599 | 0.842 | 0.443 | 1.598 | - | - | - | - |
| *Diabetes mellitus* | 0.071 | 0.593 | 0.336 | 1.046 | - | - | - | - |
| *Liver disease* | **0.014 *** | 0.352 | 0.152 | 0.810 | 0.073 | 0.372 | 0.126 | 1.097 |
| *Obesity (BMI >30)* | 0.992 | 1.005 | 0.367 | 2.754 | - | - | - | - |
| *Renal impairment* | 0.938 | 1.037 | 0.418 | 2.570 | - | - | - | - |
| *Smoking history* | **0.046 *** | 3.247 | 1.022 | 10.311 | **0.028 *** | 3.867 | 1.156 | 12.930 |
| **Neutrophils** |  |  |  |  |  |  |  |  |
| < 500 | - | - | - | - | - | - | - | - |
| 500-999 | 0.277 | 0.552 | 0.189 | 1.614 | 0.878 | 1.096 | 0.340 | 3.532 |
| ≥ 1000 | 0.089 | 0.551 | 0.278 | 1.094 | 0.249 | 1.671 | 0.698 | 4.000 |
| **Lymphocytes** |  |  |  |  |  |  |  |  |
| ≤ 200 | - | - | - | - | - | - | - | - |
| 201-499 | **<0.001 *** | 0.179 | 0.072 | 0.445 | **<0.001 *** | 0.173 | 0.063 | 0.481 |
| ≥ 500 | **<0.001 *** | 0.213 | 0.122 | 0.372 | **0.002 *** | 0.381 | 0.206 | 0.704 |
| **Baseline haematological malignancy** |  |  |  |  |  |  |  |  |
| Lymphoma | - | - | - | - | - | - | - | - |
| Plasma cell malignancies | 0.136 | 0.583 | 0.287 | 1.185 | - | - | - | - |
| Acute myeloid leukaemia | 0.182 | 0.607 | 0.291 | 1.263 | - | - | - | - |
| Chronic lymphocytic leukaemia | 0.917 | 1.043 | 0.471 | 2.313 | - | - | - | - |
| Acute lymphoblastic leukaemia | 0.862 | 1.089 | 0.417 | 2.844 | - | - | - | - |
| Myelodysplastic syndrome | 0.504 | 1.331 | 0.576 | 3.077 | - | - | - | - |
| Chronic myeloid malignancies | 0.105 | 2.001 | 0.865 | 4.628 | - | - | - | - |
| Aplastic anaemia | 0.970 | - | - | - | - | - | - | - |
| **Status haematological malignancy at infection onset** | | | | | | | | |
| Controlled malignancy | - | - | - | - | - | - | - | - |
| Active malignancy | **<0.001 *** | 3.940 | 2.233 | 6.951 | 0.068 | 1.850 | 0.956 | 3.579 |
| **Last chemotherapy strategy before infection** |  |  |  |  |  |  |  |  |
| Conventional chemotherapy | - | - | - | - | - | - | - | - |
| Demethylating agents | 0.574 | 0.753 | 0.279 | 2.027 | 0.479 | 0.558 | 0.111 | 2.799 |
| Immuno-chemotherapy | 0.194 | 0.669 | 0.365 | 1.226 | 0.259 | 0.611 | 0.260 | 1.436 |
| Targeted therapy | 0.431 | 0.740 | 0.349 | 1.566 | 0.651 | 0.787 | 0.278 | 2.224 |
| alloHSCT | **0.033 *** | 0.204 | 0.047 | 0.878 | 0.876 | 0.869 | 0.149 | 5.061 |
| autoHSCT | 0.962 | - | - | - | 0.975 | - | - | - |
| CAR-T | 0.966 | 1.045 | 0.139 | 7.828 | 0.997 | - | - | - |
| No treatment | 0.767 | 1.124 | 0.519 | 2.434 | 0.243 | 1.852 | 0.659 | 5.210 |
| Supportive measures | 0.627 | 0.607 | 0.081 | 4.549 | 0.552 | 0.492 | 0.047 | 5.105 |
| **Symptoms at viral infection onset** |  |  |  |  |  |  |  |  |
| No symptoms | - | - | - | - | - | - | - | - |
| Extrapulmonary symptoms | 0.275 | 0.631 | 0.276 | 1.441 | 0.243 | 0.551 | 0.203 | 1.498 |
| Pulmonary symptoms | **0.006 *** | 2.929 | 1.371 | 6.260 | 0.665 | 1.223 | 0.492 | 3.042 |
| **Infection treatment** |  |  |  |  |  |  |  |  |
| No treatment | - | - | - | - | - | - | - | - |
| Antivirals ± corticosteroids | 0.282 | 1.333 | 0.789 | 2.251 | 0.115 | 1.986 | 0.846 | 4.663 |
| Immunoglobulins | 0.723 | 1.437 | 0.193 | 10.689 | 0.360 | 3.142 | 0.271 | 36.402 |
| Corticosteroids | **0.008 *** | 3.187 | 1.355 | 7.496 | 0.216 | 1.955 | 0.677 | 5.645 |
| Immunoglobulins in combination | **0.031 *** | 4.929 | 1.155 | 21.032 | 0.169 | 3.457 | 0.591 | 20.222 |
| **Secondary bacterial infection** | **<0.001 *** | 4.605 | 2.881 | 7.359 | **<0.001 *** | 4.023 | 2.110 | 7.673 |
| **Secondary fungal infection** | **<0.001 *** | 4.406 | 2.320 | 8.366 | 0.331 | 1.524 | 0.652 | 3.565 |
| **Secondary viral infection** | 0.212 | 1.782 | 0.719 | 4.420 | - | - | - | - |
| **Patient stay during infection episode** |  |  |  |  |  |  |  |  |
| Home | - | - | - | - | - | - | - | - |
| Hospital, non-ICU | **<0.001 *** | 21.494 | 5.206 | 88.743 | **0.018 *** | 11.683 | 1.535 | 88.929 |
| Hospital, ICU | **<0.001 *** | 102.053 | 24.298 | 428.622 | **<0.001 *** | 49.946 | 6.462 | 386.020 |
| Not reported | 0.971 | - | - | - | 0.977 | - | - | - |

* statistically significant difference

alloHSCT, allogeneic hematopoietic stem cell transplant; autoHSCT, autologous hematopoietic stem cell transplant; BMI, body mass index; CAR-T, chimeric antigen receptor T-cell; CI, confidence interval; HR, hazard ratio; ICU, intensive care unit; RSV, respiratory syncytial virus; SARS-CoV-2, severe acute respiratory syndrome coronavirus 2

**FIGURES**

**~~Figure 1.~~** ~~Study population flowchart detailing the patients included to analysis in EPICOVIDEHA-EPIFLUEHA participants with hematological patients diagnosed with respiratory viral infections (September 2023 – March 2024).~~

~~CARV, community acquired respiratory viral infection~~

**~~Figure 2.~~** ~~Distribution of community-acquired respiratory viral infections per month in EPICOVIDEHA-EPIFLUEHA participants with hematological patients diagnosed with respiratory viral infections (September 2023 – March 2024).~~

**Figure 1~~3~~.** Community-aquired respiratory viral infection distribution per baseline haematological malignancy in EPICOVIDEHA-EPIFLUEHA participants with hematological patients diagnosed with respiratory viral infections (September 2023 – March 2024)..

**~~Figure 4.~~** ~~Treatments of the baseline haematological malignancy by month of infection diagnosis in EPICOVIDEHA-EPIFLUEHA participants with hematological patients diagnosed with respiratory viral infections (September 2023 – March 2024).~~

**~~Figure 5~~Figure 2. Day 30 survival probability by respiratory viral pathogen.**


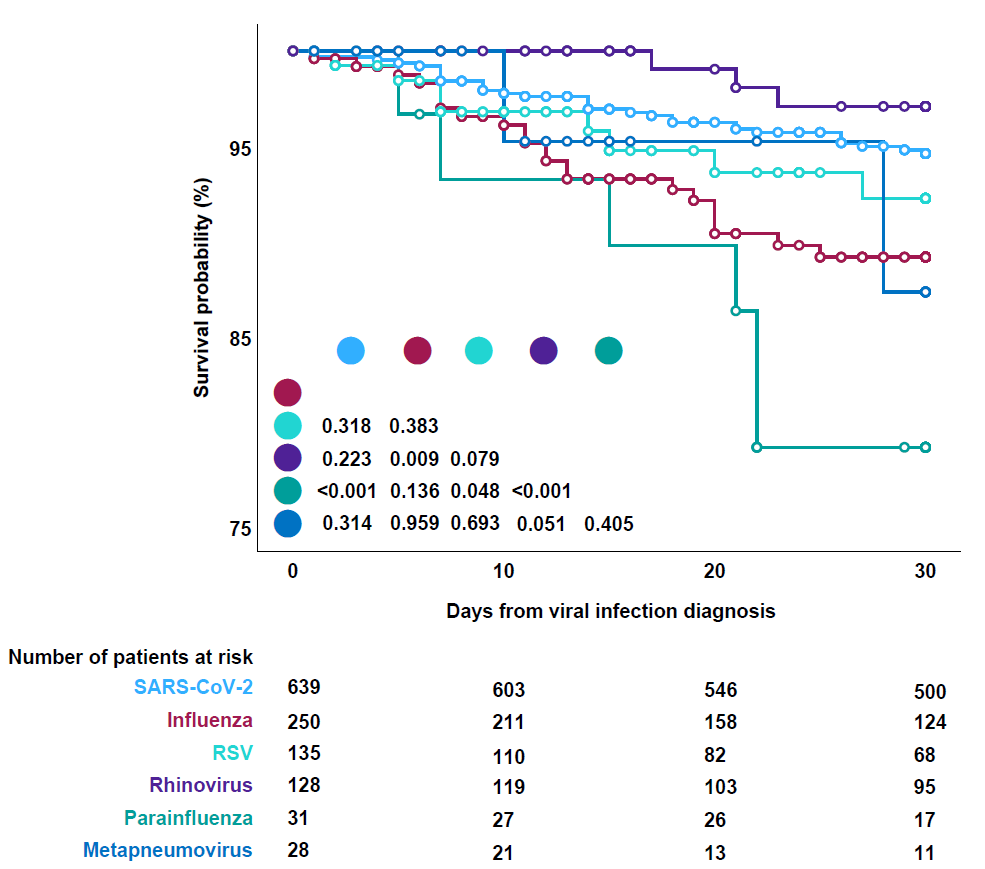


**~~SUPPLEMENTARY TABLES~~**

**~~Supplementary table 1.~~** ~~Profile of EPICOVIDEHA-EPIFLUEHA patients during the Winter season: September 2023 - March 2024: rhinovirus, parainfluenza, metapneumovirus, coronavirus non-SARS-CoV-2, enterovirus/rhinovirus, virus combinations and other virus.~~

|  | **~~Rhinovirus~~** | | **~~Parainfluenza~~** | | **~~Metapneumovirus~~** | | **~~Coronavirus non~~**  **~~SARS-CoV-2~~** | | **~~Enterovirus/~~**  **~~Rhinovirus~~** | | **~~Virus combinations *~~** | | **~~Other virus~~**  **~~**~~** | |
| --- | --- | --- | --- | --- | --- | --- | --- | --- | --- | --- | --- | --- | --- | --- |
|  | ~~n=128, 9.8%~~ | | ~~n=31, 2.4%~~ | | ~~n=28, 2.1%~~ | | ~~n=20, 1.5%~~ | | ~~n=18, 1.4%~~ | | ~~n=59, 4.5%~~ | | ~~n=4, 0.3%~~ | |
|  | ~~n~~ | ~~%~~ | ~~n~~ | ~~%~~ | ~~n~~ | ~~%~~ | ~~n~~ | ~~%~~ | ~~n~~ | ~~%~~ | ~~n~~ | ~~%~~ | ~~n~~ | ~~%~~ |
| **~~Sex~~** |  |  |  |  |  |  |  |  |  |  |  |  |  |  |
| ~~Female~~ | ~~55~~ | ~~43.0~~ | ~~13~~ | ~~41.9~~ | ~~13~~ | ~~46.4~~ | ~~10~~ | ~~50.0~~ | ~~7~~ | ~~38.9~~ | ~~27~~ | ~~45.8~~ | ~~2~~ | ~~50.0~~ |
| ~~Male~~ | ~~73~~ | ~~57.0~~ | ~~18~~ | ~~58.1~~ | ~~15~~ | ~~53.6~~ | ~~10~~ | ~~50.0~~ | ~~11~~ | ~~61.1~~ | ~~32~~ | ~~54.2~~ | ~~2~~ | ~~50.0~~ |
| **~~Age~~** | ~~59 (46-68)~~  ~~[18-96]~~ | | ~~61 (40-67)~~  ~~[22-84]~~ | | ~~60 (38-69)~~  ~~[18-95]~~ | | ~~56 (33-65)~~  ~~[19-86]~~ | | ~~58.5 (56-71)~~  ~~[36-83]~~ | | ~~65 (51-75)~~  ~~[18-90]~~ | | ~~44 (36-59)~~  ~~[32-70]~~ | |
| **~~Vaccination at infection onset~~** |  | |  | |  | |  | |  | |  | |  | |
| ~~Not vaccinated~~ | ~~128~~ | ~~100.0~~ | ~~31~~ | ~~100.0~~ | ~~28~~ | ~~100.0~~ | ~~20~~ | ~~100.0~~ | ~~18~~ | ~~100.0~~ | ~~54~~ | ~~91.5~~ | ~~4~~ | ~~100.0~~ |
| ~~Influenza~~ | ~~0~~ | ~~0.0~~ | ~~0~~ | ~~0.0~~ | ~~0~~ | ~~0.0~~ | ~~0~~ | ~~0.0~~ | ~~0~~ | ~~0.0~~ | ~~2~~ | ~~3.4~~ | ~~0~~ | ~~0.0~~ |
| ~~RSV~~ | ~~0~~ | ~~0.0~~ | ~~0~~ | ~~0.0~~ | ~~0~~ | ~~0.0~~ | ~~0~~ | ~~0.0~~ | ~~0~~ | ~~0.0~~ | ~~2~~ | ~~3.4~~ | ~~0~~ | ~~0.0~~ |
| ~~SARS-CoV-2~~ | ~~0~~ | ~~0.0~~ | ~~0~~ | ~~0.0~~ | ~~0~~ | ~~0.0~~ | ~~0~~ | ~~0.0~~ | ~~0~~ | ~~0.0~~ | ~~1~~ | ~~1.7~~ | ~~0~~ | ~~0.0~~ |
| *~~Days from last vaccination to infection~~* |  |  |  |  |  |  |  |  |  |  | ~~104 (79-125)~~  ~~[71-210]~~ | |  |  |
| **~~Comorbidities~~** |  |  |  |  |  |  |  |  |  |  |  |  |  |  |
| ~~0-1~~ | ~~102~~ | ~~79.7~~ | ~~23~~ | ~~74.2~~ | ~~23~~ | ~~82.1~~ | ~~17~~ | ~~85.0~~ | ~~8~~ | ~~44.4~~ | ~~43~~ | ~~72.9~~ | ~~3~~ | ~~75.0~~ |
| ~~2+~~ | ~~26~~ | ~~20.3~~ | ~~8~~ | ~~25.8~~ | ~~5~~ | ~~17.9~~ | ~~3~~ | ~~15.0~~ | ~~10~~ | ~~55.6~~ | ~~16~~ | ~~27.1~~ | ~~1~~ | ~~25.0~~ |
| *~~Chronic cardiopathy~~* | ~~31~~ | ~~24.2~~ | ~~10~~ | ~~32.3~~ | ~~8~~ | ~~28.6~~ | ~~5~~ | ~~25.0~~ | ~~9~~ | ~~50.0~~ | ~~18~~ | ~~30.5~~ | ~~1~~ | ~~25.0~~ |
| *~~Chronic pulmonary disease~~* | ~~17~~ | ~~13.3~~ | ~~4~~ | ~~12.9~~ | ~~3~~ | ~~10.7~~ | ~~2~~ | ~~10.0~~ | ~~7~~ | ~~38.9~~ | ~~14~~ | ~~23.7~~ | ~~1~~ | ~~25.0~~ |
| *~~Diabetes mellitus~~* | ~~13~~ | ~~10.2~~ | ~~5~~ | ~~16.1~~ | ~~1~~ | ~~3.6~~ | ~~0~~ | ~~0.0~~ | ~~6~~ | ~~33.3~~ | ~~10~~ | ~~16.9~~ | ~~0~~ | ~~0.0~~ |
| *~~Liver disease~~* | ~~5~~ | ~~3.9~~ | ~~1~~ | ~~3.2~~ | ~~0~~ | ~~0.0~~ | ~~1~~ | ~~5.0~~ | ~~1~~ | ~~5.6~~ | ~~3~~ | ~~5.1~~ | ~~1~~ | ~~25.0~~ |
| *~~Obesity (BMI >30)~~* | ~~3~~ | ~~2.3~~ | ~~1~~ | ~~3.2~~ | ~~0~~ | ~~0.0~~ | ~~2~~ | ~~10.0~~ | ~~2~~ | ~~11.1~~ | ~~1~~ | ~~1.7~~ | ~~0~~ | ~~0.0~~ |
| *~~Renal impairment~~* | ~~7~~ | ~~5.5~~ | ~~3~~ | ~~9.7~~ | ~~1~~ | ~~3.6~~ | ~~1~~ | ~~5.0~~ | ~~0~~ | ~~0.0~~ | ~~5~~ | ~~8.5~~ | ~~0~~ | ~~0.0~~ |
| *~~Smoking history~~* | ~~10~~ | ~~7.8~~ | ~~2~~ | ~~6.5~~ | ~~3~~ | ~~10.7~~ | ~~4~~ | ~~20.0~~ | ~~3~~ | ~~16.7~~ | ~~1~~ | ~~1.7~~ | ~~0~~ | ~~0.0~~ |
| **~~Neutrophils~~** |  |  |  |  |  |  |  |  |  |  |  |  |  |  |
| ~~< 500~~ | ~~13~~ | ~~10.2~~ | ~~5~~ | ~~16.1~~ | ~~1~~ | ~~3.6~~ | ~~4~~ | ~~20.0~~ | ~~2~~ | ~~11.1~~ | ~~6~~ | ~~10.2~~ | ~~1~~ | ~~25.0~~ |
| ~~500-999~~ | ~~9~~ | ~~7.0~~ | ~~4~~ | ~~12.9~~ | ~~3~~ | ~~10.7~~ | ~~1~~ | ~~5.0~~ | ~~1~~ | ~~5.6~~ | ~~2~~ | ~~3.4~~ | ~~1~~ | ~~25.0~~ |
| ~~≥ 1000~~ | ~~92~~ | ~~71.9~~ | ~~19~~ | ~~61.3~~ | ~~19~~ | ~~67.9~~ | ~~12~~ | ~~60.0~~ | ~~15~~ | ~~83.3~~ | ~~43~~ | ~~72.9~~ | ~~2~~ | ~~50.0~~ |
| **~~Lymphocytes~~** |  |  |  |  |  |  |  |  |  |  |  |  |  |  |
| ~~≤ 200~~ | ~~17~~ | ~~13.3~~ | ~~5~~ | ~~16.1~~ | ~~4~~ | ~~14.3~~ | ~~1~~ | ~~5.0~~ | ~~1~~ | ~~5.6~~ | ~~8~~ | ~~13.6~~ | ~~2~~ | ~~50.0~~ |
| ~~201-499~~ | ~~16~~ | ~~12.5~~ | ~~5~~ | ~~16.1~~ | ~~0~~ | ~~0.0~~ | ~~3~~ | ~~15.0~~ | ~~2~~ | ~~11.1~~ | ~~8~~ | ~~13.6~~ | ~~0~~ | ~~0.0~~ |
| ~~≥ 500~~ | ~~77~~ | ~~60.2~~ | ~~17~~ | ~~54.8~~ | ~~18~~ | ~~64.3~~ | ~~14~~ | ~~70.0~~ | ~~13~~ | ~~72.2~~ | ~~35~~ | ~~59.3~~ | ~~2~~ | ~~50.0~~ |
| **~~Baseline haematological malignancy~~** |  |  |  |  |  |  |  |  |  |  |  |  |  |  |
| ~~Lymphoma~~ | ~~37~~ | ~~28.9~~ | ~~13~~ | ~~41.9~~ | ~~8~~ | ~~28.6~~ | ~~7~~ | ~~35.0~~ | ~~4~~ | ~~22.2~~ | ~~16~~ | ~~27.1~~ | ~~2~~ | ~~50.0~~ |
| *~~Hodgkin lymphoma~~* | ~~6~~ | ~~4.7~~ | ~~2~~ | ~~6.5~~ | ~~4~~ | ~~14.3~~ | ~~0~~ | ~~0.0~~ | ~~1~~ | ~~5.6~~ | ~~1~~ | ~~1.7~~ | ~~0~~ | ~~0.0~~ |
| *~~Non-Hodgkin lymphoma~~* | ~~31~~ | ~~24.2~~ | ~~11~~ | ~~35.5~~ | ~~4~~ | ~~14.3~~ | ~~7~~ | ~~35.0~~ | ~~3~~ | ~~16.7~~ | ~~15~~ | ~~25.4~~ | ~~2~~ | ~~50.0~~ |
| ~~Plasma cell malignancies~~ | ~~22~~ | ~~17.2~~ | ~~3~~ | ~~9.7~~ | ~~5~~ | ~~17.9~~ | ~~3~~ | ~~15.0~~ | ~~4~~ | ~~22.2~~ | ~~15~~ | ~~25.4~~ | ~~0~~ | ~~0.0~~ |
| *~~Amyloid light-chain amyloidosis~~* | ~~1~~ | ~~0.8~~ | ~~0~~ | ~~0.0~~ | ~~1~~ | ~~3.6~~ | ~~0~~ | ~~0.0~~ | ~~0~~ | ~~0.0~~ | ~~0~~ | ~~0.0~~ | ~~0~~ | ~~0.0~~ |
| *~~Multiple myeloma~~* | ~~21~~ | ~~16.4~~ | ~~3~~ | ~~9.7~~ | ~~4~~ | ~~14.3~~ | ~~3~~ | ~~15.0~~ | ~~4~~ | ~~22.2~~ | ~~15~~ | ~~25.4~~ | ~~0~~ | ~~0.0~~ |
| ~~Acute myeloid leukaemia~~ | ~~34~~ | ~~26.6~~ | ~~9~~ | ~~29.0~~ | ~~3~~ | ~~10.7~~ | ~~4~~ | ~~20.0~~ | ~~4~~ | ~~22.2~~ | ~~9~~ | ~~15.3~~ | ~~1~~ | ~~25.0~~ |
| ~~Acute lymphoblastic leukaemia~~ | ~~11~~ | ~~8.6~~ | ~~1~~ | ~~3.2~~ | ~~1~~ | ~~3.6~~ | ~~4~~ | ~~20.0~~ | ~~3~~ | ~~16.7~~ | ~~6~~ | ~~10.2~~ | ~~1~~ | ~~25.0~~ |
| ~~Chronic lymphocytic leukaemia~~ | ~~6~~ | ~~4.7~~ | ~~1~~ | ~~3.2~~ | ~~0~~ | ~~0.0~~ | ~~0~~ | ~~0.0~~ | ~~1~~ | ~~5.6~~ | ~~8~~ | ~~13.6~~ | ~~0~~ | ~~0.0~~ |
| *~~Chronic lymphocytic leukaemia~~* | ~~5~~ | ~~3.9~~ | ~~1~~ | ~~3.2~~ | ~~0~~ | ~~0.0~~ | ~~0~~ | ~~0.0~~ | ~~0~~ | ~~0.0~~ | ~~8~~ | ~~13.6~~ | ~~0~~ | ~~0.0~~ |
| *~~Hairy cell leukaemia~~* | ~~1~~ | ~~0.8~~ | ~~0~~ | ~~0.0~~ | ~~0~~ | ~~0.0~~ | ~~0~~ | ~~0.0~~ | ~~1~~ | ~~5.6~~ | ~~0~~ | ~~0.0~~ | ~~0~~ | ~~0.0~~ |
| ~~Myelodysplastic syndrome~~ | ~~11~~ | ~~8.6~~ | ~~1~~ | ~~3.2~~ | ~~3~~ | ~~10.7~~ | ~~0~~ | ~~0.0~~ | ~~1~~ | ~~5.6~~ | ~~3~~ | ~~5.1~~ | ~~0~~ | ~~0.0~~ |
| ~~Chronic myeloid malignancies~~ | ~~6~~ | ~~4.7~~ | ~~2~~ | ~~6.5~~ | ~~8~~ | ~~28.6~~ | ~~0~~ | ~~0.0~~ | ~~1~~ | ~~5.6~~ | ~~2~~ | ~~3.4~~ | ~~0~~ | ~~0.0~~ |
| *~~Chronic myeloid leukaemia~~* | ~~4~~ | ~~3.1~~ | ~~2~~ | ~~6.5~~ | ~~3~~ | ~~10.7~~ | ~~0~~ | ~~0.0~~ | ~~0~~ | ~~0.0~~ | ~~1~~ | ~~1.7~~ | ~~0~~ | ~~0.0~~ |
| *~~Myelofibrosis~~* | ~~1~~ | ~~0.8~~ | ~~0~~ | ~~0.0~~ | ~~3~~ | ~~10.7~~ | ~~0~~ | ~~0.0~~ | ~~1~~ | ~~5.6~~ | ~~0~~ | ~~0.0~~ | ~~0~~ | ~~0.0~~ |
| *~~Essential thrombocythemia~~* | ~~0~~ | ~~0.0~~ | ~~0~~ | ~~0.0~~ | ~~1~~ | ~~3.6~~ | ~~0~~ | ~~0.0~~ | ~~0~~ | ~~0.0~~ | ~~1~~ | ~~1.7~~ | ~~0~~ | ~~0.0~~ |
| *~~Polycythaemia vera~~* | ~~1~~ | ~~0.8~~ | ~~0~~ | ~~0.0~~ | ~~1~~ | ~~3.6~~ | ~~0~~ | ~~0.0~~ | ~~0~~ | ~~0.0~~ | ~~0~~ | ~~0.0~~ | ~~0~~ | ~~0.0~~ |
| *~~Systemic mastocytosis~~* | ~~0~~ | ~~0.0~~ | ~~0~~ | ~~0.0~~ | ~~0~~ | ~~0.0~~ | ~~0~~ | ~~0.0~~ | ~~0~~ | ~~0.0~~ | ~~0~~ | ~~0.0~~ | ~~0~~ | ~~0.0~~ |
| ~~Aplastic anaemia~~ | ~~1~~ | ~~0.8~~ | ~~1~~ | ~~3.2~~ | ~~0~~ | ~~0.0~~ | ~~2~~ | ~~10.0~~ | ~~0~~ | ~~0.0~~ | ~~0~~ | ~~0.0~~ | ~~0~~ | ~~0.0~~ |
| **~~Status haematological malignancy at infection onset~~** |  |  |  |  |  |  |  |  |  |  |  |  |  |  |
| ~~Controlled malignancy~~ | ~~71~~ | ~~55.5~~ | ~~12~~ | ~~38.7~~ | ~~12~~ | ~~42.9~~ | ~~12~~ | ~~60.0~~ | ~~13~~ | ~~72.2~~ | ~~31~~ | ~~52.5~~ | ~~3~~ | ~~75.0~~ |
| ~~Active malignancy~~ | ~~57~~ | ~~44.5~~ | ~~19~~ | ~~61.3~~ | ~~16~~ | ~~57.1~~ | ~~8~~ | ~~40.0~~ | ~~5~~ | ~~27.8~~ | ~~28~~ | ~~47.5~~ | ~~1~~ | ~~25.0~~ |
| **~~Last chemotherapy strategy before infection~~** |  |  |  |  |  |  |  |  |  |  |  |  |  |  |
| ~~Conventional chemotherapy~~ | ~~28~~ | ~~21.9~~ | ~~8~~ | ~~25.8~~ | ~~5~~ | ~~17.9~~ | ~~3~~ | ~~15.0~~ | ~~1~~ | ~~5.6~~ | ~~11~~ | ~~18.6~~ | ~~1~~ | ~~25.0~~ |
| *~~< 3 months~~* | ~~22~~ | ~~17.2~~ | ~~7~~ | ~~22.6~~ | ~~4~~ | ~~14.3~~ | ~~3~~ | ~~15.0~~ | ~~1~~ | ~~5.6~~ | ~~7~~ | ~~11.9~~ | ~~1~~ | ~~25.0~~ |
| *~~> 3 months~~* | ~~6~~ | ~~4.7~~ | ~~1~~ | ~~3.2~~ | ~~1~~ | ~~3.6~~ | ~~0~~ | ~~0.0~~ | ~~0~~ | ~~0.0~~ | ~~4~~ | ~~6.8~~ | ~~0~~ | ~~0.0~~ |
| ~~Demethylating agents~~ | ~~4~~ | ~~3.1~~ | ~~1~~ | ~~3.2~~ | ~~3~~ | ~~10.7~~ | ~~0~~ | ~~0.0~~ | ~~3~~ | ~~16.7~~ | ~~2~~ | ~~3.4~~ | ~~0~~ | ~~0.0~~ |
| *~~< 3 months~~* | ~~4~~ | ~~3.1~~ | ~~1~~ | ~~3.2~~ | ~~2~~ | ~~7.1~~ | ~~0~~ | ~~0.0~~ | ~~3~~ | ~~16.7~~ | ~~1~~ | ~~1.7~~ | ~~0~~ | ~~0.0~~ |
| *~~> 3 months~~* | ~~0~~ | ~~0.0~~ | ~~0~~ | ~~0.0~~ | ~~1~~ | ~~3.6~~ | ~~0~~ | ~~0.0~~ | ~~0~~ | ~~0.0~~ | ~~1~~ | ~~1.7~~ | ~~0~~ | ~~0.0~~ |
| ~~Immuno-chemotherapy~~ | ~~23~~ | ~~18.0~~ | ~~11~~ | ~~35.5~~ | ~~4~~ | ~~14.3~~ | ~~8~~ | ~~40.0~~ | ~~6~~ | ~~33.3~~ | ~~23~~ | ~~39.0~~ | ~~1~~ | ~~25.0~~ |
| *~~< 3 months~~* | ~~21~~ | ~~16.4~~ | ~~7~~ | ~~22.6~~ | ~~3~~ | ~~10.7~~ | ~~6~~ | ~~30.0~~ | ~~6~~ | ~~33.3~~ | ~~17~~ | ~~28.8~~ | ~~0~~ | ~~0.0~~ |
| *~~> 3 months~~* | ~~2~~ | ~~1.6~~ | ~~4~~ | ~~12.9~~ | ~~1~~ | ~~3.6~~ | ~~2~~ | ~~10.0~~ | ~~0~~ | ~~0.0~~ | ~~6~~ | ~~10.2~~ | ~~1~~ | ~~25.0~~ |
| ~~Targeted therapy~~ | ~~15~~ | ~~11.7~~ | ~~3~~ | ~~9.7~~ | ~~2~~ | ~~7.1~~ | ~~0~~ | ~~0.0~~ | ~~2~~ | ~~11.1~~ | ~~4~~ | ~~6.8~~ | ~~0~~ | ~~0.0~~ |
| *~~< 3 months~~* | ~~13~~ | ~~10.2~~ | ~~2~~ | ~~6.5~~ | ~~2~~ | ~~7.1~~ | ~~0~~ | ~~0.0~~ | ~~2~~ | ~~11.1~~ | ~~4~~ | ~~6.8~~ | ~~0~~ | ~~0.0~~ |
| *~~> 3 months~~* | ~~2~~ | ~~1.6~~ | ~~1~~ | ~~3.2~~ | ~~0~~ | ~~0.0~~ | ~~0~~ | ~~0.0~~ | ~~0~~ | ~~0.0~~ | ~~0~~ | ~~0.0~~ | ~~0~~ | ~~0.0~~ |
| ~~alloHSCT~~ | ~~32~~ | ~~25.0~~ | ~~3~~ | ~~9.7~~ | ~~8~~ | ~~28.6~~ | ~~5~~ | ~~25.0~~ | ~~3~~ | ~~16.7~~ | ~~7~~ | ~~11.9~~ | ~~1~~ | ~~25.0~~ |
| *~~< 6 months~~* | ~~16~~ | ~~12.5~~ | ~~2~~ | ~~6.5~~ | ~~3~~ | ~~10.7~~ | ~~1~~ | ~~5.0~~ | ~~2~~ | ~~11.1~~ | ~~1~~ | ~~1.7~~ | ~~1~~ | ~~25.0~~ |
| *~~> 6 months~~* | ~~16~~ | ~~12.5~~ | ~~1~~ | ~~3.2~~ | ~~5~~ | ~~17.9~~ | ~~4~~ | ~~20.0~~ | ~~1~~ | ~~5.6~~ | ~~6~~ | ~~10.2~~ | ~~0~~ | ~~0.0~~ |
| ~~autoHSCT~~ | ~~10~~ | ~~7.8~~ | ~~2~~ | ~~6.5~~ | ~~1~~ | ~~3.6~~ | ~~1~~ | ~~5.0~~ | ~~1~~ | ~~5.6~~ | ~~2~~ | ~~3.4~~ | ~~0~~ | ~~0.0~~ |
| *~~< 6 months~~* | ~~10~~ | ~~7.8~~ | ~~1~~ | ~~3.2~~ | ~~1~~ | ~~3.6~~ | ~~1~~ | ~~5.0~~ | ~~1~~ | ~~5.6~~ | ~~2~~ | ~~3.4~~ | ~~0~~ | ~~0.0~~ |
| *~~> 6 months~~* | ~~0~~ | ~~0.0~~ | ~~1~~ | ~~3.2~~ | ~~0~~ | ~~0.0~~ | ~~0~~ | ~~0.0~~ | ~~0~~ | ~~0.0~~ | ~~0~~ | ~~0.0~~ | ~~0~~ | ~~0.0~~ |
| ~~CAR-T~~ | ~~1~~ | ~~0.8~~ | ~~2~~ | ~~6.5~~ | ~~0~~ | ~~0.0~~ | ~~1~~ | ~~5.0~~ | ~~1~~ | ~~5.6~~ | ~~0~~ | ~~0.0~~ | ~~1~~ | ~~25.0~~ |
| *~~< 6 months~~* | ~~1~~ | ~~0.8~~ | ~~2~~ | ~~6.5~~ | ~~0~~ | ~~0.0~~ | ~~1~~ | ~~5.0~~ | ~~1~~ | ~~5.6~~ | ~~0~~ | ~~0.0~~ | ~~0~~ | ~~0.0~~ |
| *~~> 6 months~~* | ~~0~~ | ~~0.0~~ | ~~0~~ | ~~0.0~~ | ~~0~~ | ~~0.0~~ | ~~0~~ | ~~0.0~~ | ~~0~~ | ~~0.0~~ | ~~0~~ | ~~0.0~~ | ~~1~~ | ~~25.0~~ |
| ~~No treatment~~ | ~~12~~ | ~~9.4~~ | ~~1~~ | ~~3.2~~ | ~~4~~ | ~~14.3~~ | ~~0~~ | ~~0.0~~ | ~~1~~ | ~~5.6~~ | ~~10~~ | ~~16.9~~ | ~~0~~ | ~~0.0~~ |
| ~~Supportive measures~~ | ~~3~~ | ~~2.3~~ | ~~0~~ | ~~0.0~~ | ~~1~~ | ~~3.6~~ | ~~2~~ | ~~10.0~~ | ~~0~~ | ~~0.0~~ | ~~0~~ | ~~0.0~~ | ~~0~~ | ~~0.0~~ |
| **~~Viral diagnosis month~~** |  |  |  |  |  |  |  |  |  |  |  |  |  |  |
| ~~September 2023~~ | ~~15~~ | ~~11.7~~ | ~~8~~ | ~~25.8~~ | ~~1~~ | ~~3.6~~ | ~~0~~ | ~~0.0~~ | ~~4~~ | ~~22.2~~ | ~~2~~ | ~~3.4~~ | ~~0~~ | ~~0.0~~ |
| ~~October 2023~~ | ~~20~~ | ~~15.6~~ | ~~2~~ | ~~6.5~~ | ~~0~~ | ~~0.0~~ | ~~4~~ | ~~20.0~~ | ~~5~~ | ~~27.8~~ | ~~2~~ | ~~3.4~~ | ~~0~~ | ~~0.0~~ |
| ~~November 2023~~ | ~~26~~ | ~~20.3~~ | ~~8~~ | ~~25.8~~ | ~~0~~ | ~~0.0~~ | ~~2~~ | ~~10.0~~ | ~~2~~ | ~~11.1~~ | ~~7~~ | ~~11.9~~ | ~~0~~ | ~~0.0~~ |
| ~~December 2023~~ | ~~24~~ | ~~18.8~~ | ~~2~~ | ~~6.5~~ | ~~3~~ | ~~10.7~~ | ~~2~~ | ~~10.0~~ | ~~2~~ | ~~11.1~~ | ~~9~~ | ~~15.3~~ | ~~2~~ | ~~50.0~~ |
| ~~January 2024~~ | ~~16~~ | ~~12.5~~ | ~~4~~ | ~~12.9~~ | ~~6~~ | ~~21.4~~ | ~~2~~ | ~~10.0~~ | ~~0~~ | ~~0.0~~ | ~~18~~ | ~~30.5~~ | ~~0~~ | ~~0.0~~ |
| ~~February 2024~~ | ~~17~~ | ~~13.3~~ | ~~3~~ | ~~9.7~~ | ~~3~~ | ~~10.7~~ | ~~4~~ | ~~20.0~~ | ~~3~~ | ~~16.7~~ | ~~14~~ | ~~23.7~~ | ~~1~~ | ~~25.0~~ |
| ~~March 2024~~ | ~~10~~ | ~~7.8~~ | ~~4~~ | ~~12.9~~ | ~~15~~ | ~~53.6~~ | ~~6~~ | ~~30.0~~ | ~~2~~ | ~~11.1~~ | ~~7~~ | ~~11.9~~ | ~~1~~ | ~~25.0~~ |
| **~~Symptoms at viral infection onset~~** |  |  |  |  |  |  |  |  |  |  |  |  |  |  |
| ~~No symptoms~~ | ~~111~~ | ~~86.7~~ | ~~29~~ | ~~93.5~~ | ~~20~~ | ~~71.4~~ | ~~17~~ | ~~85.0~~ | ~~12~~ | ~~66.7~~ | ~~18~~ | ~~30.5~~ | ~~3~~ | ~~75.0~~ |
| ~~Extrapulmonary symptoms~~ | ~~3~~ | ~~2.3~~ | ~~0~~ | ~~0.0~~ | ~~1~~ | ~~3.6~~ | ~~1~~ | ~~5.0~~ | ~~0~~ | ~~0.0~~ | ~~34~~ | ~~57.6~~ | ~~0~~ | ~~0.0~~ |
| ~~Pulmonary symptoms~~ | ~~9~~ | ~~7.0~~ | ~~0~~ | ~~0.0~~ | ~~2~~ | ~~7.1~~ | ~~0~~ | ~~0.0~~ | ~~1~~ | ~~5.6~~ | ~~0~~ | ~~0.0~~ | ~~0~~ | ~~0.0~~ |
| **~~Viral infection severity~~** |  |  |  |  |  |  |  |  |  |  |  |  |  |  |
| ~~Asymptomatic~~ | ~~12~~ | ~~9.4~~ | ~~1~~ | ~~3.2~~ | ~~0~~ | ~~0.0~~ | ~~1~~ | ~~5.0~~ | ~~0~~ | ~~0.0~~ | ~~0~~ | ~~0.0~~ | ~~0~~ | ~~0.0~~ |
| ~~Mild~~ | ~~96~~ | ~~75.0~~ | ~~18~~ | ~~58.1~~ | ~~17~~ | ~~60.7~~ | ~~17~~ | ~~85.0~~ | ~~10~~ | ~~55.6~~ | ~~40~~ | ~~67.8~~ | ~~3~~ | ~~75.0~~ |
| ~~Severe~~ | ~~15~~ | ~~11.7~~ | ~~7~~ | ~~22.6~~ | ~~7~~ | ~~25.0~~ | ~~1~~ | ~~5.0~~ | ~~7~~ | ~~38.9~~ | ~~12~~ | ~~20.3~~ | ~~0~~ | ~~0.0~~ |
| ~~Critical~~ | ~~5~~ | ~~3.9~~ | ~~5~~ | ~~16.1~~ | ~~4~~ | ~~14.3~~ | ~~1~~ | ~~5.0~~ | ~~1~~ | ~~5.6~~ | ~~7~~ | ~~11.9~~ | ~~1~~ | ~~25.0~~ |
| **~~Viral infection treatment~~** |  |  |  |  |  |  |  |  |  |  |  |  |  |  |
| ~~No treatment~~ | ~~111~~ | ~~86.7~~ | ~~29~~ | ~~93.5~~ | ~~20~~ | ~~71.4~~ | ~~17~~ | ~~85.0~~ | ~~12~~ | ~~66.7~~ | ~~18~~ | ~~30.5~~ | ~~3~~ | ~~75.0~~ |
| ~~Antivirals ± corticosteroids~~ | ~~3~~ | ~~2.3~~ | ~~0~~ | ~~0.0~~ | ~~1~~ | ~~3.6~~ | ~~1~~ | ~~5.0~~ | ~~0~~ | ~~0.0~~ | ~~34~~ | ~~54.6~~ | ~~0~~ | ~~0.0~~ |
| ~~Immunoglobulins~~ | ~~9~~ | ~~7.0~~ | ~~0~~ | ~~0.0~~ | ~~2~~ | ~~7.1~~ | ~~0~~ | ~~0.0~~ | ~~1~~ | ~~5.6~~ | ~~0~~ | ~~0.0~~ | ~~0~~ | ~~0.0~~ |
| ~~Corticosteroids~~ | ~~5~~ | ~~3.9~~ | ~~2~~ | ~~6.5~~ | ~~5~~ | ~~17.9~~ | ~~2~~ | ~~10.0~~ | ~~5~~ | ~~27.8~~ | ~~7~~ | ~~11.9~~ | ~~1~~ | ~~25.0~~ |
| ~~Immunoglobulins in combination~~ | ~~0~~ | ~~0.0~~ | ~~0~~ | ~~0.0~~ | ~~0~~ | ~~0.0~~ | ~~0~~ | ~~0.0~~ | ~~0~~ | ~~0.0~~ | ~~0~~ | ~~0.0~~ | ~~0~~ | ~~0.0~~ |
| **~~Secondary infections~~** | ~~36~~ | ~~28.1~~ | ~~8~~ | ~~25.8~~ | ~~4~~ | ~~14.3~~ | ~~2~~ | ~~10.0~~ | ~~7~~ | ~~38.9~~ | ~~16~~ | ~~27.1~~ | ~~0~~ | ~~0.0~~ |
| ~~Bacterial~~ | ~~27~~ | ~~21.1~~ | ~~5~~ | ~~16.1~~ | ~~2~~ | ~~7.1~~ | ~~1~~ | ~~5.0~~ | ~~7~~ | ~~38.9~~ | ~~11~~ | ~~18.6~~ | ~~0~~ | ~~0.0~~ |
| ~~Fungal~~ | ~~6~~ | ~~4.7~~ | ~~3~~ | ~~9.7~~ | ~~0~~ | ~~0.0~~ | ~~0~~ | ~~0.0~~ | ~~0~~ | ~~0.0~~ | ~~4~~ | ~~6.8~~ | ~~0~~ | ~~0.0~~ |
| ~~Other viral~~ | ~~12~~ | ~~9.4~~ | ~~2~~ | ~~6.5~~ | ~~3~~ | ~~10.7~~ | ~~1~~ | ~~5.0~~ | ~~1~~ | ~~5.6~~ | ~~4~~ | ~~6.8~~ | ~~0~~ | ~~0.0~~ |
| **~~Patient stay during viral infection~~** |  |  |  |  |  |  |  |  |  |  |  |  | ~~0~~ | ~~0.0~~ |
| ~~Home~~ | ~~46~~ | ~~35.9~~ | ~~12~~ | ~~38.7~~ | ~~11~~ | ~~39.3~~ | ~~9~~ | ~~45.0~~ | ~~4~~ | ~~22.2~~ | ~~19~~ | ~~32.2~~ | ~~2~~ | ~~50.0~~ |
| ~~Hospital~~ | ~~79~~ | ~~61.7~~ | ~~17~~ | ~~54.8~~ | ~~17~~ | ~~60.7~~ | ~~10~~ | ~~50.0~~ | ~~12~~ | ~~66.7~~ | ~~40~~ | ~~67.8~~ | ~~2~~ | ~~50.0~~ |
| *~~Hospital, non-ICU~~* | ~~74~~ | ~~57.8~~ | ~~12~~ | ~~38.7~~ | ~~13~~ | ~~46.4~~ | ~~9~~ | ~~45.0~~ | ~~11~~ | ~~61.1~~ | ~~33~~ | ~~55.9~~ | ~~1~~ | ~~25.0~~ |
| *~~Hospital, ICU~~* | ~~5~~ | ~~3.9~~ | ~~5~~ | ~~16.1~~ | ~~4~~ | ~~14.3~~ | ~~1~~ | ~~5.0~~ | ~~1~~ | ~~5.6~~ | ~~7~~ | ~~11.9~~ | ~~1~~ | ~~25.0~~ |
| *~~Invasive MV~~* | ~~1~~ | ~~20.0~~ | ~~3~~ | ~~60.0~~ | ~~3~~ | ~~75.0~~ | ~~0~~ | ~~0.0~~ | ~~0~~ | ~~0.0~~ | ~~2~~ | ~~28.6~~ | ~~1~~ | ~~100.0~~ |
| *~~Non-invasive MV~~* | ~~2~~ | ~~40.0~~ | ~~1~~ | ~~20.0~~ | ~~1~~ | ~~25.0~~ | ~~0~~ | ~~0.0~~ | ~~1~~ | ~~100.0~~ | ~~4~~ | ~~57.1~~ | ~~0~~ | ~~0.0~~ |
| ~~Not reported~~ | ~~3~~ | ~~60.0~~ | ~~2~~ | ~~40.0~~ | ~~0~~ | ~~0.0~~ | ~~1~~ | ~~100.0~~ | ~~2~~ | ~~200.0~~ | ~~0~~ | ~~0.0~~ | ~~0~~ | ~~0.0~~ |
| **~~Mortality, d30~~** | ~~3~~ | ~~2.3~~ | ~~6~~ | ~~19.4~~ | ~~2~~ | ~~7.1~~ | ~~2~~ | ~~10.0~~ | ~~0~~ | ~~0.0~~ | ~~2~~ | ~~3.4~~ | ~~0~~ | ~~0.0~~ |
| ~~Reason for mortality~~ |  |  |  |  |  |  |  |  |  |  |  |  |  |  |
| *~~Haematological malignancy~~* | ~~2~~ | ~~66.7~~ | ~~4~~ | ~~66.7~~ | ~~2~~ | ~~100.0~~ | ~~1~~ | ~~50.0~~ | ~~0~~ | ~~0.0~~ | ~~0~~ | ~~0.0~~ | ~~0~~ | ~~0.0~~ |
| *~~Viral infection~~* | ~~0~~ | ~~0.0~~ | ~~3~~ | ~~50.0~~ | ~~1~~ | ~~50.0~~ | ~~0~~ | ~~0.0~~ | ~~0~~ | ~~0.0~~ | ~~2~~ | ~~100.0~~ | ~~0~~ | ~~0.0~~ |
| *~~Other reasons~~* | ~~3~~ | ~~100.0~~ | ~~3~~ | ~~50.0~~ | ~~2~~ | ~~100.0~~ | ~~2~~ | ~~100.0~~ | ~~0~~ | ~~0.0~~ | ~~0~~ | ~~0.0~~ | ~~0~~ | ~~0.0~~ |

~~* Combined infections: influenza + SARS-CoV-2 (n=12, 0.9%), RSV + SARS-CoV-2 (n=11, 0.8%), influenza + parainfluenza (n=6, 0.5%), influenza + RSV and rhinovirus + SARS-CoV-2 (n=4, 0.3% each), influenza + rhinovirus and metapneumovirus + SARS-CoV-2 n=3 each, coronavirus non-SARS-CoV-2 + rhinovirus, parainfluenza + SARS-CoV-2, and rhinovirus + RSV (n=2, 0.2% each) and adenovirus + rhinovirus, adenovirus + SARS-CoV-2, bocavirus + rhinovirus, coronavirus non SARS-CoV-2 + metapneumovirus + rhinovirus, coronavirus non-SARS-CoV-2 + metapneumovirus, coronavirus non-SARS-CoV-2 + parainfluenza, coronavirus non-SARS-CoV-2 + rhinovirus + RSV, influenza + parainfluenza + rhinovirus, metapneumovirus + RSV, and rhinovirus + RSV + SARS-CoV-2 (n=1, 0.1% each)~~

~~** Other virus: adenovirus n=2, bocavirus n=2, 0.2% each)~~

~~alloHSCT, allogeneic hematopoietic stem cell transplantation; autoHSCT, autologous hematopoietic stem cell transplantation; BMI, body mass index; CAR-T, chimeric antigen receptor T-cell therapy; d30, day 30; ICU, intensive care unit; n, number; MV, mechanical ventilation; RSV, respiratory syncytial virus; SARS-CoV-2, severe acute respiratory syndrome coronavirus 2~~

**~~Supplementary table 2.~~** ~~Factors associated with increased mortality in SARS-CoV-2 in EPICOVIDEHA-EPIFLUEHA participants with hematological patients diagnosed with respiratory viral infections (September 2023 – March 2024).~~

|  | **~~Univariable analysis~~** | | | | **~~Multivariable analysis~~** | | | |
| --- | --- | --- | --- | --- | --- | --- | --- | --- |
|  | **~~p~~** | **~~HR~~** | **~~95% CI~~** | | **~~p~~** | **~~HR~~** | **~~95% CI~~** | |
|  |  |  | **~~Lower~~** | **~~Upper~~** |  |  | **~~Lower~~** | **~~Upper~~** |
| **~~Sex~~** |  |  |  |  |  |  |  |  |
| ~~Female~~ | ~~-~~ | ~~-~~ | ~~-~~ | ~~-~~ | ~~-~~ | ~~-~~ | ~~-~~ | ~~-~~ |
| ~~Male~~ | ~~0.968~~ | ~~1.014~~ | ~~0.507~~ | ~~2.031~~ | ~~-~~ | ~~-~~ | ~~-~~ | ~~-~~ |
| **~~Age~~** | ~~0.136~~ | ~~1.021~~ | ~~0.994~~ | ~~1.049~~ | ~~-~~ | ~~-~~ | ~~-~~ | ~~-~~ |
| **~~Vaccination at infection onset~~** | ~~0.901~~ | ~~0.881~~ | ~~0.120~~ | ~~6.457~~ | ~~-~~ | ~~-~~ | ~~-~~ | ~~-~~ |
| **~~Comorbidities~~** |  |  |  |  |  |  |  |  |
| ~~0-1~~ | ~~-~~ | ~~-~~ | ~~-~~ | ~~-~~ | ~~-~~ | ~~-~~ | ~~-~~ | ~~-~~ |
| ~~2+~~ | ~~0.994~~ | ~~0.997~~ | ~~0.472~~ | ~~2.105~~ | ~~-~~ | ~~-~~ | ~~-~~ | ~~-~~ |
| *~~Chronic cardiopathy~~* | ~~0.791~~ | ~~1.098~~ | ~~0.549~~ | ~~2.196~~ | ~~-~~ | ~~-~~ | ~~-~~ | ~~-~~ |
| *~~Chronic pulmonary disease~~* | ~~0.585~~ | ~~1.305~~ | ~~0.502~~ | ~~3.388~~ | ~~-~~ | ~~-~~ | ~~-~~ | ~~-~~ |
| *~~Diabetes mellitus~~* | ~~0.485~~ | ~~1.372~~ | ~~0.565~~ | ~~3.334~~ | ~~-~~ | ~~-~~ | ~~-~~ | ~~-~~ |
| *~~Liver disease~~* | ~~0.145~~ | ~~2.901~~ | ~~0.693~~ | ~~12.139~~ | ~~-~~ | ~~-~~ | ~~-~~ | ~~-~~ |
| *~~Obesity (BMI >30)~~* | ~~0.352~~ | ~~0.045~~ | ~~<0.001~~ | ~~30.556~~ | ~~-~~ | ~~-~~ | ~~-~~ | ~~-~~ |
| *~~Renal impairment~~* | ~~0.800~~ | ~~1.166~~ | ~~0.355~~ | ~~3.828~~ | ~~-~~ | ~~-~~ | ~~-~~ | ~~-~~ |
| *~~Smoking history~~* | ~~0.170~~ | ~~0.041~~ | ~~<0.001~~ | ~~3.946~~ | ~~-~~ | ~~-~~ | ~~-~~ | ~~-~~ |
| **~~Neutrophils~~** |  |  |  |  |  |  |  |  |
| ~~< 500~~ | ~~-~~ | ~~-~~ | ~~-~~ | ~~-~~ | ~~-~~ | ~~-~~ | ~~-~~ | ~~-~~ |
| ~~500-999~~ | ~~0.538~~ | ~~0.587~~ | ~~0.107~~ | ~~3.203~~ | ~~-~~ | ~~-~~ | ~~-~~ | ~~-~~ |
| ~~≥ 1000~~ | ~~0.238~~ | ~~0.524~~ | ~~0.179~~ | ~~1.533~~ | ~~-~~ | ~~-~~ | ~~-~~ | ~~-~~ |
| **~~Lymphocytes~~** |  |  |  |  |  |  |  |  |
| ~~≤ 200~~ | ~~-~~ | ~~-~~ | ~~-~~ | ~~-~~ | ~~-~~ | ~~-~~ | ~~-~~ | ~~-~~ |
| ~~201-499~~ | **~~0.038 *~~** | ~~0.272~~ | ~~0.080~~ | ~~0.930~~ | **~~0.011 *~~** | ~~0.177~~ | ~~0.047~~ | ~~0.672~~ |
| ~~≥ 500~~ | **~~<0.001 *~~** | ~~0.167~~ | ~~0.066~~ | ~~0.424~~ | **~~0.001 *~~** | ~~0.170~~ | ~~0.059~~ | ~~0.492~~ |
| **~~Baseline haematological malignancy~~** |  |  |  |  |  |  |  |  |
| ~~Lymphoma~~ | ~~-~~ | ~~-~~ | ~~-~~ | ~~-~~ | ~~-~~ | ~~-~~ | ~~-~~ | ~~-~~ |
| ~~Plasma cell malignancies~~ | ~~0.177~~ | ~~0.462~~ | ~~0.151~~ | ~~1.418~~ | ~~0.871~~ | ~~1.107~~ | ~~0.326~~ | ~~3.754~~ |
| ~~Acute myeloid leukaemia~~ | ~~0.068~~ | ~~0.151~~ | ~~0.020~~ | ~~1.154~~ | ~~0.058~~ | ~~0.134~~ | ~~0.017~~ | ~~1.069~~ |
| ~~Chronic lymphocytic leukaemia~~ | ~~0.496~~ | ~~0.647~~ | ~~0.184~~ | ~~2.270~~ | ~~0.802~~ | ~~0.761~~ | ~~0.091~~ | ~~6.387~~ |
| ~~Acute lymphoblastic leukaemia~~ | ~~0.508~~ | ~~1.528~~ | ~~0.435~~ | ~~5.361~~ | ~~0.901~~ | ~~1.105~~ | ~~0.228~~ | ~~5.371~~ |
| ~~Myelodysplastic syndrome~~ | ~~0.078~~ | ~~2.527~~ | ~~0.901~~ | ~~7.090~~ | **~~0.005 *~~** | ~~6.102~~ | ~~1.705~~ | ~~21.842~~ |
| ~~Chronic myeloid malignancies~~ | ~~0.271~~ | ~~2.024~~ | ~~0.577~~ | ~~7.104~~ | ~~0.068~~ | ~~4.551~~ | ~~0.894~~ | ~~23.161~~ |
| ~~Aplastic anaemia~~ | ~~0.974~~ | ~~-~~ | ~~-~~ | ~~-~~ | ~~0.987~~ | ~~-~~ | ~~-~~ | ~~-~~ |
| **~~Status haematological malignancy at infection onset~~** |  |  |  |  |  |  |  |  |
| ~~Controlled malignancy~~ | ~~-~~ | ~~-~~ | ~~-~~ | ~~-~~ | ~~-~~ | ~~-~~ | ~~-~~ | ~~-~~ |
| ~~Active malignancy~~ | **~~<0.001 *~~** | ~~5.355~~ | ~~2.062~~ | ~~13.905~~ | **~~0.036 *~~** | ~~3.844~~ | ~~1.093~~ | ~~13.519~~ |
| **~~Last chemotherapy strategy before infection~~** |  |  |  |  |  |  |  |  |
| ~~Conventional chemotherapy~~ | ~~-~~ | ~~-~~ | ~~-~~ | ~~-~~ | ~~-~~ | ~~-~~ | ~~-~~ | ~~-~~ |
| ~~Demethylating agents~~ | ~~0.986~~ | ~~0.986~~ | ~~0.199~~ | ~~4.884~~ | ~~-~~ | ~~-~~ | ~~-~~ | ~~-~~ |
| ~~Immuno-chemotherapy~~ | ~~0.870~~ | ~~0.924~~ | ~~0.358~~ | ~~2.381~~ | ~~-~~ | ~~-~~ | ~~-~~ | ~~-~~ |
| ~~Targeted therapy~~ | ~~0.548~~ | ~~0.679~~ | ~~0.192~~ | ~~2.405~~ | ~~-~~ | ~~-~~ | ~~-~~ | ~~-~~ |
| ~~alloHSCT~~ | ~~0.979~~ | ~~<0.001~~ | ~~<0.001~~ | ~~-~~ | ~~-~~ | ~~-~~ | ~~-~~ | ~~-~~ |
| ~~autoHSCT~~ | ~~0.987~~ | ~~<0.001~~ | ~~<0.001~~ | ~~-~~ | ~~-~~ | ~~-~~ | ~~-~~ | ~~-~~ |
| ~~CAR-T~~ | ~~0.993~~ | ~~<0.001~~ | ~~<0.001~~ | ~~-~~ | ~~-~~ | ~~-~~ | ~~-~~ | ~~-~~ |
| ~~No treatment~~ | ~~0.605~~ | ~~1.368~~ | ~~0.417~~ | ~~4.482~~ | ~~-~~ | ~~-~~ | ~~-~~ | ~~-~~ |
| ~~Supportive measures~~ | ~~0.991~~ | ~~<0.001~~ | ~~<0.001~~ | ~~-~~ | ~~-~~ | ~~-~~ | ~~-~~ | ~~-~~ |
| **~~Symptoms at viral infection onset~~** |  |  |  |  |  |  |  |  |
| ~~No symptoms~~ | ~~-~~ | ~~-~~ | ~~-~~ | ~~-~~ | ~~-~~ | ~~-~~ | ~~-~~ | ~~-~~ |
| ~~Extrapulmonary symptoms~~ | ~~0.204~~ | ~~0.535~~ | ~~0.204~~ | ~~1.405~~ | ~~-~~ | ~~-~~ | ~~-~~ | ~~-~~ |
| ~~Pulmonary symptoms~~ | ~~0.202~~ | ~~1.853~~ | ~~0.718~~ | ~~4.780~~ | ~~-~~ | ~~-~~ | ~~-~~ | ~~-~~ |
| **~~Infection treatment~~** |  |  |  |  |  |  |  |  |
| ~~No treatment~~ | ~~-~~ | ~~-~~ | ~~-~~ | ~~-~~ | ~~-~~ | ~~-~~ | ~~-~~ | ~~-~~ |
| ~~Antivirals ± corticosteroids~~ | ~~0.645~~ | ~~1.220~~ | ~~0.523~~ | ~~2.843~~ | ~~-~~ | ~~-~~ | ~~-~~ | ~~-~~ |
| ~~Corticosteroids~~ | ~~0.111~~ | ~~3.585~~ | ~~0.744~~ | ~~17.261~~ | ~~-~~ | ~~-~~ | ~~-~~ | ~~-~~ |
| ~~Immunoglobulins in combination~~ | ~~0.974~~ | ~~-~~ | ~~-~~ | ~~-~~ | ~~-~~ | ~~-~~ | ~~-~~ | ~~-~~ |
| **~~Secondary bacterial infection~~** | ~~0.075~~ | ~~2.144~~ | ~~0.927~~ | ~~4.956~~ | ~~0.417~~ | ~~1.528~~ | ~~0.549~~ | ~~4.251~~ |
| **~~Secondary fungal infection~~** | **~~<0.001 *~~** | ~~7.732~~ | ~~3.182~~ | ~~18.791~~ | ~~0.037 *~~ | ~~3.761~~ | ~~1.084~~ | ~~13.051~~ |
| **~~Secondary viral infection~~** | ~~0.983~~ | ~~0.978~~ | ~~0.134~~ | ~~7.165~~ | ~~-~~ | ~~-~~ | ~~-~~ | ~~-~~ |
| **~~Patient stay during infection episode~~** |  |  |  |  |  |  |  |  |
| ~~Home~~ | ~~-~~ | ~~-~~ | ~~-~~ | ~~-~~ | ~~-~~ | ~~-~~ | ~~-~~ | ~~-~~ |
| ~~Hospital, non-ICU~~ | **~~0.001 *~~** | ~~12.742~~ | ~~2.978~~ | ~~54.514~~ | **~~0.014 *~~** | ~~13.319~~ | ~~1.704~~ | ~~104.137~~ |
| ~~Hospital, ICU~~ | **~~<0.001 *~~** | ~~45.717~~ | ~~10.014~~ | ~~208.714~~ | **~~0.001 *~~** | ~~39.351~~ | ~~4.384~~ | ~~353.185~~ |
| ~~Not reported~~ | ~~0.981~~ | ~~-~~ | ~~-~~ | ~~-~~ | ~~0.995~~ | ~~-~~ | ~~-~~ | ~~-~~ |

~~* statistically significant difference~~

~~alloHSCT, allogeneic hematopoietic stem cell transplant; autoHSCT, autologous hematopoietic stem cell transplant; BMI, body mass index; CAR-T, chimeric antigen receptor T-cell; CI, confidence interval; HR, hazard ratio; ICU, intensive care unit; SARS-CoV-2, severe acute respiratory syndrome coronavirus 2~~

**~~Supplementary table 3.~~** ~~Factors associated with increased mortality in influenza in EPICOVIDEHA-EPIFLUEHA participants with hematological patients diagnosed with respiratory viral infections (September 2023 – March 2024).~~

|  | **~~Univariable analysis~~** | | | | **~~Multivariable analysis~~** | | | |
| --- | --- | --- | --- | --- | --- | --- | --- | --- |
|  | ~~p~~ | ~~HR~~ | ~~95% CI~~ | | ~~p~~ | ~~HR~~ | ~~95% CI~~ | |
|  |  |  | ~~Lower~~ | ~~Upper~~ |  |  | ~~Lower~~ | ~~Upper~~ |
| **~~Sex~~** |  |  |  |  |  |  |  |  |
| ~~Female~~ | ~~-~~ | ~~-~~ | ~~-~~ | ~~-~~ | ~~-~~ | ~~-~~ | ~~-~~ | ~~-~~ |
| ~~Male~~ | ~~0.647~~ | ~~1.225~~ | ~~0.514~~ | ~~2.921~~ | ~~-~~ | ~~-~~ | ~~-~~ | ~~-~~ |
| **~~Age~~** | **~~0.031 *~~** | ~~1.039~~ | ~~1.003~~ | ~~1.076~~ | ~~0.139~~ | ~~1.033~~ | ~~0.990~~ | ~~1.077~~ |
| **~~Vaccination at infection onset~~** | ~~0.864~~ | ~~1.099~~ | ~~0.372~~ | ~~3.247~~ | ~~-~~ | ~~-~~ | ~~-~~ | ~~-~~ |
| **~~Comorbidities~~** |  |  |  |  |  |  |  |  |
| ~~0-1~~ | ~~-~~ | ~~-~~ | ~~-~~ | ~~-~~ | ~~-~~ | ~~-~~ | ~~-~~ | ~~-~~ |
| ~~2+~~ | ~~0.288~~ | ~~1.586~~ | ~~0.677~~ | ~~3.715~~ | ~~-~~ | ~~-~~ | ~~-~~ | ~~-~~ |
| *~~Chronic cardiopathy~~* | ~~0.328~~ | ~~1.529~~ | ~~0.653~~ | ~~3.577~~ | ~~-~~ | ~~-~~ | ~~-~~ | ~~-~~ |
| *~~Chronic pulmonary disease~~* | ~~0.854~~ | ~~1.107~~ | ~~0.374~~ | ~~3.274~~ | ~~-~~ | ~~-~~ | ~~-~~ | ~~-~~ |
| *~~Diabetes mellitus~~* | ~~0.152~~ | ~~2.073~~ | ~~0.765~~ | ~~5.621~~ | ~~-~~ | ~~-~~ | ~~-~~ | ~~-~~ |
| *~~Liver disease~~* | ~~0.290~~ | ~~2.191~~ | ~~0.512~~ | ~~9.378~~ | ~~-~~ | ~~-~~ | ~~-~~ | ~~-~~ |
| *~~Obesity (BMI >30)~~* | ~~0.168~~ | ~~2.356~~ | ~~0.697~~ | ~~7.962~~ | ~~-~~ | ~~-~~ | ~~-~~ | ~~-~~ |
| *~~Renal impairment~~* | ~~0.447~~ | ~~1.759~~ | ~~0.411~~ | ~~7.526~~ | ~~-~~ | ~~-~~ | ~~-~~ | ~~-~~ |
| *~~Smoking history~~* | ~~0.486~~ | ~~0.596~~ | ~~0.139~~ | ~~2.551~~ | ~~-~~ | ~~-~~ | ~~-~~ | ~~-~~ |
| **~~Neutrophils~~** |  |  |  |  |  |  |  |  |
| ~~< 500~~ | ~~-~~ | ~~-~~ | ~~-~~ | ~~-~~ | ~~-~~ | ~~-~~ | ~~-~~ | ~~-~~ |
| ~~500-999~~ | ~~0.467~~ | ~~0.410~~ | ~~0.037~~ | ~~4.522~~ | ~~-~~ | ~~-~~ | ~~-~~ | ~~-~~ |
| ~~≥ 1000~~ | ~~0.802~~ | ~~0.828~~ | ~~0.189~~ | ~~3.624~~ | ~~-~~ | ~~-~~ | ~~-~~ | ~~-~~ |
| **~~Lymphocytes~~** |  |  |  |  |  |  |  |  |
| ~~≤ 200~~ | ~~-~~ | ~~-~~ | ~~-~~ | ~~-~~ | ~~-~~ | ~~-~~ | ~~-~~ | ~~-~~ |
| ~~201-499~~ | **~~0.021 *~~** | ~~0.087~~ | ~~0.011~~ | ~~0.696~~ | **~~0.008 *~~** | ~~0.058~~ | ~~0.007~~ | ~~0.479~~ |
| ~~≥ 500~~ | **~~0.005 *~~** | ~~0.252~~ | ~~0.097~~ | ~~0.655~~ | **~~0.006 *~~** | ~~0.260~~ | ~~0.099~~ | ~~0.684~~ |
| **~~Baseline haematological malignancy~~** |  |  |  |  |  |  |  |  |
| ~~Lymphoma~~ | ~~-~~ | ~~-~~ | ~~-~~ | ~~-~~ | ~~-~~ | ~~-~~ | ~~-~~ | ~~-~~ |
| ~~Plasma cell malignancies~~ | ~~0.994~~ | ~~0.995~~ | ~~0.267~~ | ~~3.708~~ | ~~-~~ | ~~-~~ | ~~-~~ | ~~-~~ |
| ~~Acute myeloid leukaemia~~ | ~~0.318~~ | ~~1.870~~ | ~~0.548~~ | ~~6.390~~ | ~~-~~ | ~~-~~ | ~~-~~ | ~~-~~ |
| ~~Chronic lymphocytic leukaemia~~ | ~~0.219~~ | ~~2.559~~ | ~~0.573~~ | ~~11.434~~ | ~~-~~ | ~~-~~ | ~~-~~ | ~~-~~ |
| ~~Acute lymphoblastic leukaemia~~ | ~~0.627~~ | ~~1.722~~ | ~~0.192~~ | ~~15.417~~ | ~~-~~ | ~~-~~ | ~~-~~ | ~~-~~ |
| ~~Myelodysplastic syndrome~~ | ~~0.833~~ | ~~1.201~~ | ~~0.220~~ | ~~6.558~~ | ~~-~~ | ~~-~~ | ~~-~~ | ~~-~~ |
| ~~Chronic myeloid malignancies~~ | ~~0.984~~ | ~~-~~ | ~~-~~ | ~~-~~ | ~~-~~ | ~~-~~ | ~~-~~ | ~~-~~ |
| ~~Aplastic anaemia~~ | ~~0.993~~ | ~~-~~ | ~~-~~ | ~~-~~ | ~~-~~ | ~~-~~ | ~~-~~ | ~~-~~ |
| **~~Status haematological malignancy at infection onset~~** |  |  |  |  |  |  |  |  |
| ~~Controlled malignancy~~ | ~~-~~ | ~~-~~ | ~~-~~ | ~~-~~ | ~~-~~ | ~~-~~ | ~~-~~ | ~~-~~ |
| ~~Active malignancy~~ | ~~0.058~~ | ~~2.482~~ | ~~0.971~~ | ~~6.345~~ | ~~0.227~~ | ~~1.969~~ | ~~0.656~~ | ~~5.910~~ |
| **~~Last chemotherapy strategy before infection~~** |  |  |  |  |  |  |  |  |
| ~~Conventional chemotherapy~~ | ~~-~~ | ~~-~~ | ~~-~~ | ~~-~~ | ~~-~~ | ~~-~~ | ~~-~~ | ~~-~~ |
| ~~Demethylating agents~~ | ~~0.763~~ | ~~0.802~~ | ~~0.191~~ | **~~3.361~~** | ~~-~~ | ~~-~~ | ~~-~~ | ~~-~~ |
| ~~Immuno-chemotherapy~~ | ~~0.168~~ | ~~0.396~~ | ~~0.106~~ | ~~1.477~~ | ~~-~~ | ~~-~~ | ~~-~~ | ~~-~~ |
| ~~Targeted therapy~~ | ~~0.622~~ | ~~1.348~~ | ~~0.411~~ | ~~4.420~~ | ~~-~~ | ~~-~~ | ~~-~~ | ~~-~~ |
| ~~alloHSCT~~ | ~~0.984~~ | ~~-~~ | ~~-~~ | ~~-~~ | ~~-~~ | ~~-~~ | ~~-~~ | ~~-~~ |
| ~~autoHSCT~~ | ~~0.987~~ | ~~-~~ | ~~-~~ | ~~-~~ | ~~-~~ | ~~-~~ | ~~-~~ | ~~-~~ |
| ~~CAR-T~~ | ~~0.995~~ | ~~-~~ | ~~-~~ | ~~-~~ | ~~-~~ | ~~-~~ | ~~-~~ | ~~-~~ |
| ~~No treatment~~ | ~~0.331~~ | ~~1.921~~ | ~~0.516~~ | ~~7.158~~ | ~~-~~ | ~~-~~ | ~~-~~ | ~~-~~ |
| ~~Supportive measures~~ | ~~0.990~~ | ~~-~~ | ~~-~~ | ~~-~~ | ~~-~~ | ~~-~~ | ~~-~~ | ~~-~~ |
| **~~Symptoms at viral infection onset~~** |  |  |  |  |  |  |  |  |
| ~~No symptoms~~ | ~~-~~ | ~~-~~ | ~~-~~ | ~~-~~ | ~~-~~ | ~~-~~ | ~~-~~ | ~~-~~ |
| ~~Extrapulmonary symptoms~~ | ~~0.937~~ | ~~-~~ | ~~-~~ | ~~-~~ | ~~-~~ | ~~-~~ | ~~-~~ | ~~-~~ |
| ~~Pulmonary symptoms~~ | ~~0.924~~ | ~~-~~ | ~~-~~ | ~~-~~ | ~~-~~ | ~~-~~ | ~~-~~ | ~~-~~ |
| **~~Infection treatment~~** |  |  |  |  |  |  |  |  |
| ~~No treatment~~ | ~~-~~ | ~~-~~ | ~~-~~ | ~~-~~ | ~~-~~ | ~~-~~ | ~~-~~ | ~~-~~ |
| ~~Antivirals ± corticosteroids~~ | ~~0.638~~ | ~~1.342~~ | ~~0.395~~ | ~~4.555~~ | ~~-~~ | ~~-~~ | ~~-~~ | ~~-~~ |
| ~~Corticosteroids~~ | ~~0.982~~ | ~~-~~ | ~~-~~ | ~~-~~ | ~~-~~ | ~~-~~ | ~~-~~ | ~~-~~ |
| ~~Immunoglobulins in combination~~ | ~~0.056~~ | ~~9.132~~ | ~~0.943~~ | ~~88.462~~ | ~~-~~ | ~~-~~ | ~~-~~ | ~~-~~ |
| **~~Secondary bacterial infection~~** | **~~<0.001 *~~** | ~~8.945~~ | ~~3.816~~ | ~~20.969~~ | **~~<0.001 *~~** | ~~10.837~~ | ~~4.085~~ | ~~28.750~~ |
| **~~Secondary fungal infection~~** | ~~0.088~~ | ~~2.884~~ | ~~0.853~~ | ~~9.748~~ | ~~0.162~~ | ~~2.451~~ | ~~0.697~~ | ~~8.623~~ |
| **~~Secondary viral infection~~** | **~~0.017 *~~** | ~~5.893~~ | ~~1.375~~ | ~~25.257~~ | ~~0.581~~ | ~~1.666~~ | ~~0.272~~ | ~~10.216~~ |
| **~~Patient stay during infection episode~~** |  |  |  |  |  |  |  |  |
| ~~Home~~ | ~~-~~ | ~~-~~ | ~~-~~ | ~~-~~ | ~~-~~ | ~~-~~ | ~~-~~ | ~~-~~ |
| ~~Hospital, non-ICU~~ | ~~0.908~~ | ~~-~~ | ~~-~~ | ~~-~~ | ~~-~~ | ~~-~~ | ~~-~~ | ~~-~~ |
| ~~Hospital, ICU~~ | ~~0.899~~ | ~~-~~ | ~~-~~ | ~~-~~ | ~~-~~ | ~~-~~ | ~~-~~ | ~~-~~ |
| ~~Not reported~~ | ~~1.000~~ | ~~-~~ | ~~-~~ | ~~-~~ | ~~-~~ | ~~-~~ | ~~-~~ | ~~-~~ |

~~* statistically significant difference~~

~~alloHSCT, allogeneic hematopoietic stem cell transplant; autoHSCT, autologous hematopoietic stem cell transplant; BMI, body mass index; CAR-T, chimeric antigen receptor T-cell; CI, confidence interval; HR, hazard ratio; ICU, intensive care unit~~

**~~Supplementary table 4.~~** ~~Factors associated with increased mortality in respiratory syncytial virus infection in EPICOVIDEHA-EPIFLUEHA participants with hematological patients diagnosed with respiratory viral infections (September 2023 – March 2024).~~

|  | **~~Univariable analysis~~** | | | | **~~Multivariable analysis~~** | | | |
| --- | --- | --- | --- | --- | --- | --- | --- | --- |
|  | **~~p~~** | **~~HR~~** | **~~95% CI~~** | | **~~p~~** | **~~HR~~** | **~~95% CI~~** | |
|  |  |  | **~~Lower~~** | **~~Upper~~** |  |  | **~~Lower~~** | **~~Upper~~** |
| **~~Sex~~** |  |  |  |  |  |  |  |  |
| ~~Female~~ | ~~-~~ | ~~-~~ | ~~-~~ | ~~-~~ | ~~-~~ | ~~-~~ | ~~-~~ | ~~-~~ |
| ~~Male~~ | ~~0.131~~ | ~~5.031~~ | ~~0.619~~ | ~~40.903~~ | ~~-~~ | ~~-~~ | ~~-~~ | ~~-~~ |
| **~~Age~~** | ~~0.666~~ | ~~1.011~~ | ~~0.961~~ | ~~1.064~~ | ~~-~~ | ~~-~~ | ~~-~~ | ~~-~~ |
| **~~Vaccination at infection onset~~** | ~~0.863~~ | ~~-~~ | ~~-~~ | ~~-~~ | ~~-~~ | ~~-~~ | ~~-~~ | ~~-~~ |
| **~~Comorbidities~~** |  |  |  |  |  |  |  |  |
| ~~0-1~~ | ~~-~~ | ~~-~~ | ~~-~~ | ~~-~~ | ~~-~~ | ~~-~~ | ~~-~~ | ~~-~~ |
| ~~2+~~ | ~~0.475~~ | ~~1.685~~ | ~~0.402~~ | ~~7.059~~ | ~~-~~ | ~~-~~ | ~~-~~ | ~~-~~ |
| *~~Chronic cardiopathy~~* | ~~0.926~~ | ~~1.070~~ | ~~0.255~~ | ~~4.481~~ | ~~-~~ | ~~-~~ | ~~-~~ | ~~-~~ |
| *~~Chronic pulmonary disease~~* | ~~0.829~~ | ~~1.260~~ | ~~0.155~~ | ~~10.249~~ | ~~-~~ | ~~-~~ | ~~-~~ | ~~-~~ |
| *~~Diabetes mellitus~~* | ~~0.344~~ | ~~2.164~~ | ~~0.437~~ | ~~10.726~~ | ~~-~~ | ~~-~~ | ~~-~~ | ~~-~~ |
| *~~Liver disease~~* | ~~0.341~~ | ~~2.767~~ | ~~0.340~~ | ~~22.504~~ | ~~-~~ | ~~-~~ | ~~-~~ | ~~-~~ |
| *~~Obesity (BMI >30)~~* | ~~0.582~~ | ~~1.803~~ | ~~0.221~~ | ~~14.675~~ | ~~-~~ | ~~-~~ | ~~-~~ | ~~-~~ |
| *~~Renal impairment~~* | ~~0.675~~ | ~~-~~ | ~~-~~ | ~~-~~ | ~~-~~ | ~~-~~ | ~~-~~ | ~~-~~ |
| *~~Smoking history~~* | ~~0.523~~ | ~~-~~ | ~~-~~ | ~~-~~ | ~~-~~ | ~~-~~ | ~~-~~ | ~~-~~ |
| **~~Neutrophils~~** |  |  |  |  |  |  |  |  |
| ~~< 500~~ | ~~-~~ | ~~-~~ | ~~-~~ | ~~-~~ | ~~-~~ | ~~-~~ | ~~-~~ | ~~-~~ |
| ~~500-999~~ | ~~0.839~~ | ~~0.780~~ | ~~0.071~~ | ~~8.606~~ | ~~-~~ | ~~-~~ | ~~-~~ | ~~-~~ |
| ~~≥ 1000~~ | ~~0.544~~ | ~~0.601~~ | ~~0.116~~ | ~~3.106~~ | ~~-~~ | ~~-~~ | ~~-~~ | ~~-~~ |
| **~~Lymphocytes~~** |  |  |  |  |  |  |  |  |
| ~~≤ 200~~ | ~~-~~ | ~~-~~ | ~~-~~ | ~~-~~ | ~~-~~ | ~~-~~ | ~~-~~ | ~~-~~ |
| ~~201-499~~ | ~~0.109~~ | ~~0.165~~ | ~~0.018~~ | ~~1.491~~ | ~~0.300~~ | ~~0.300~~ | ~~0.031~~ | ~~2.932~~ |
| ~~≥ 500~~ | **~~0.029 *~~** | ~~0.188~~ | ~~0.042~~ | ~~0.845~~ | ~~0.225~~ | ~~0.370~~ | ~~0.074~~ | ~~1.845~~ |
| **~~Baseline haematological malignancy~~** |  |  |  |  |  |  |  |  |
| ~~Lymphoma~~ | ~~-~~ | ~~-~~ | ~~-~~ | ~~-~~ | ~~-~~ | ~~-~~ | ~~-~~ | ~~-~~ |
| ~~Plasma cell malignancies~~ | ~~0.160~~ | ~~0.214~~ | ~~0.025~~ | ~~1.839~~ | ~~-~~ | ~~-~~ | ~~-~~ | ~~-~~ |
| ~~Acute myeloid leukaemia~~ | ~~0.165~~ | ~~0.218~~ | ~~0.025~~ | ~~1.874~~ | ~~-~~ | ~~-~~ | ~~-~~ | ~~-~~ |
| ~~Chronic lymphocytic leukaemia~~ | ~~0.784~~ | ~~0.741~~ | ~~0.087~~ | ~~6.346~~ | ~~-~~ | ~~-~~ | ~~-~~ | ~~-~~ |
| ~~Acute lymphoblastic leukaemia~~ | ~~0.988~~ | ~~-~~ | ~~-~~ | ~~-~~ | ~~-~~ | ~~-~~ | ~~-~~ | ~~-~~ |
| ~~Myelodysplastic syndrome~~ | ~~0.991~~ | ~~-~~ | ~~-~~ | ~~-~~ | ~~-~~ | ~~-~~ | ~~-~~ | ~~-~~ |
| ~~Chronic myeloid malignancies~~ | ~~0.997~~ | ~~-~~ | ~~-~~ | ~~-~~ | ~~-~~ | ~~-~~ | ~~-~~ | ~~-~~ |
| ~~Aplastic anaemia~~ | ~~0.997~~ | ~~-~~ | ~~-~~ | ~~-~~ | ~~-~~ | ~~-~~ | ~~-~~ | ~~-~~ |
| **~~Status haematological malignancy at infection onset~~** |  |  |  |  |  |  |  |  |
| ~~Controlled malignancy~~ | ~~-~~ | ~~-~~ | ~~-~~ | ~~-~~ | ~~-~~ | ~~-~~ | ~~-~~ | ~~-~~ |
| ~~Active malignancy~~ | ~~0.167~~ | ~~3.093~~ | ~~0.623~~ | ~~15.347~~ | ~~-~~ | ~~-~~ | ~~-~~ | ~~-~~ |
| **~~Last chemotherapy strategy before infection~~** |  |  |  |  |  |  |  |  |
| ~~Conventional chemotherapy~~ | ~~-~~ | ~~-~~ | ~~-~~ | ~~-~~ | ~~-~~ | ~~-~~ | ~~-~~ | ~~-~~ |
| ~~Demethylating agents~~ | ~~0.991~~ | ~~-~~ | ~~-~~ | ~~-~~ | ~~-~~ | ~~-~~ | ~~-~~ | ~~-~~ |
| ~~Immuno-chemotherapy~~ | ~~0.474~~ | ~~1.822~~ | ~~0.353~~ | ~~9.394~~ | ~~-~~ | ~~-~~ | ~~-~~ | ~~-~~ |
| ~~Targeted therapy~~ | ~~0.879~~ | ~~0.830~~ | ~~0.075~~ | ~~9.165~~ | ~~-~~ | ~~-~~ | ~~-~~ | ~~-~~ |
| ~~alloHSCT~~ | ~~0.986~~ | ~~-~~ | ~~-~~ | ~~-~~ | ~~-~~ | ~~-~~ | ~~-~~ | ~~-~~ |
| ~~autoHSCT~~ | ~~0.994~~ | ~~-~~ | ~~-~~ | ~~-~~ | ~~-~~ | ~~-~~ | ~~-~~ | ~~-~~ |
| ~~CAR-T~~ | ~~0.995~~ | ~~-~~ | ~~-~~ | ~~-~~ | ~~-~~ | ~~-~~ | ~~-~~ | ~~-~~ |
| ~~No treatment~~ | ~~0.989~~ | ~~-~~ | ~~-~~ | ~~-~~ | ~~-~~ | ~~-~~ | ~~-~~ | ~~-~~ |
| ~~Supportive measures~~ | ~~0.994~~ | ~~-~~ | ~~-~~ | ~~-~~ | ~~-~~ | ~~-~~ | ~~-~~ | ~~-~~ |
| **~~Symptoms at viral infection onset~~** |  |  |  |  |  |  |  |  |
| ~~No symptoms~~ | ~~-~~ | ~~-~~ | ~~-~~ | ~~-~~ | ~~-~~ | ~~-~~ | ~~-~~ | ~~-~~ |
| ~~Extrapulmonary symptoms~~ | ~~0.959~~ | ~~-~~ | ~~-~~ | ~~-~~ | ~~-~~ | ~~-~~ | ~~-~~ | ~~-~~ |
| ~~Pulmonary symptoms~~ | ~~0.951~~ | ~~-~~ | ~~-~~ | ~~-~~ | ~~-~~ | ~~-~~ | ~~-~~ | ~~-~~ |
| **~~Infection treatment~~** |  |  |  |  |  |  |  |  |
| ~~No treatment~~ | ~~-~~ | ~~-~~ | ~~-~~ | ~~-~~ | ~~-~~ | ~~-~~ | ~~-~~ | ~~-~~ |
| ~~Antivirals ± corticosteroids~~ | ~~0.974~~ | ~~0.964~~ | ~~0.108~~ | ~~8.630~~ | ~~-~~ | ~~-~~ | ~~-~~ | ~~-~~ |
| ~~Immunoglobulins~~ | ~~0.991~~ | ~~-~~ | ~~-~~ | ~~-~~ | ~~-~~ | ~~-~~ | ~~-~~ | ~~-~~ |
| ~~Corticosteroids~~ | ~~0.199~~ | ~~3.043~~ | ~~0.557~~ | ~~16.632~~ | ~~-~~ | ~~-~~ | ~~-~~ | ~~-~~ |
| ~~Immunoglobulins in combination~~ | **~~0.034 *~~** | ~~10.701~~ | ~~1.192~~ | ~~96.109~~ | ~~-~~ | ~~-~~ | ~~-~~ | ~~-~~ |
| **~~Secondary bacterial infection~~** | **~~0.003 *~~** | ~~11.050~~ | ~~2.230~~ | ~~54.758~~ | **~~0.006 *~~** | ~~9.830~~ | ~~1.902~~ | ~~50.815~~ |
| **~~Secondary fungal infection~~** | **~~0.044 *~~** | ~~5.217~~ | ~~1.049~~ | ~~25.955~~ | ~~0.866~~ | ~~1.264~~ | ~~0.083~~ | ~~19.229~~ |
| **~~Secondary viral infection~~** | **~~0.013 *~~** | ~~7.536~~ | ~~1.520~~ | ~~37.365~~ | **~~0.031 *~~** | ~~6.298~~ | ~~1.182~~ | ~~33.551~~ |
| **~~Patient stay during infection episode~~** |  |  |  |  |  |  |  |  |
| ~~Home~~ | ~~-~~ | ~~-~~ | ~~-~~ | ~~-~~ | ~~-~~ | ~~-~~ | ~~-~~ | ~~-~~ |
| ~~Hospital, non-ICU~~ | ~~0.936~~ | ~~-~~ | ~~-~~ | ~~-~~ | ~~-~~ | ~~-~~ | ~~-~~ | ~~-~~ |
| ~~Hospital, ICU~~ | ~~0.921~~ | ~~-~~ | ~~-~~ | ~~-~~ | ~~-~~ | ~~-~~ | ~~-~~ | ~~-~~ |
| ~~Not reported~~ | ~~1.000~~ | ~~-~~ | ~~-~~ | ~~-~~ | ~~-~~ | ~~-~~ | ~~-~~ | ~~-~~ |

~~* statistically significant difference~~

~~alloHSCT, allogeneic hematopoietic stem cell transplant; autoHSCT, autologous hematopoietic stem cell transplant; BMI, body mass index; CAR-T, chimeric antigen receptor T-cell; CI, confidence interval; HR, hazard ratio; ICU, intensive care unit; RSV, respiratory syncytial virus~~

**~~SUPPLEMENTARY FIGURES~~**

**~~Supplementary figure 1.~~** ~~Geographical distribution of EPICOVIDEHA-EPIFLUEHA participants with haematological patients diagnosed with respiratory viral infections during the winter season September 2023 - March 2024.~~

~~
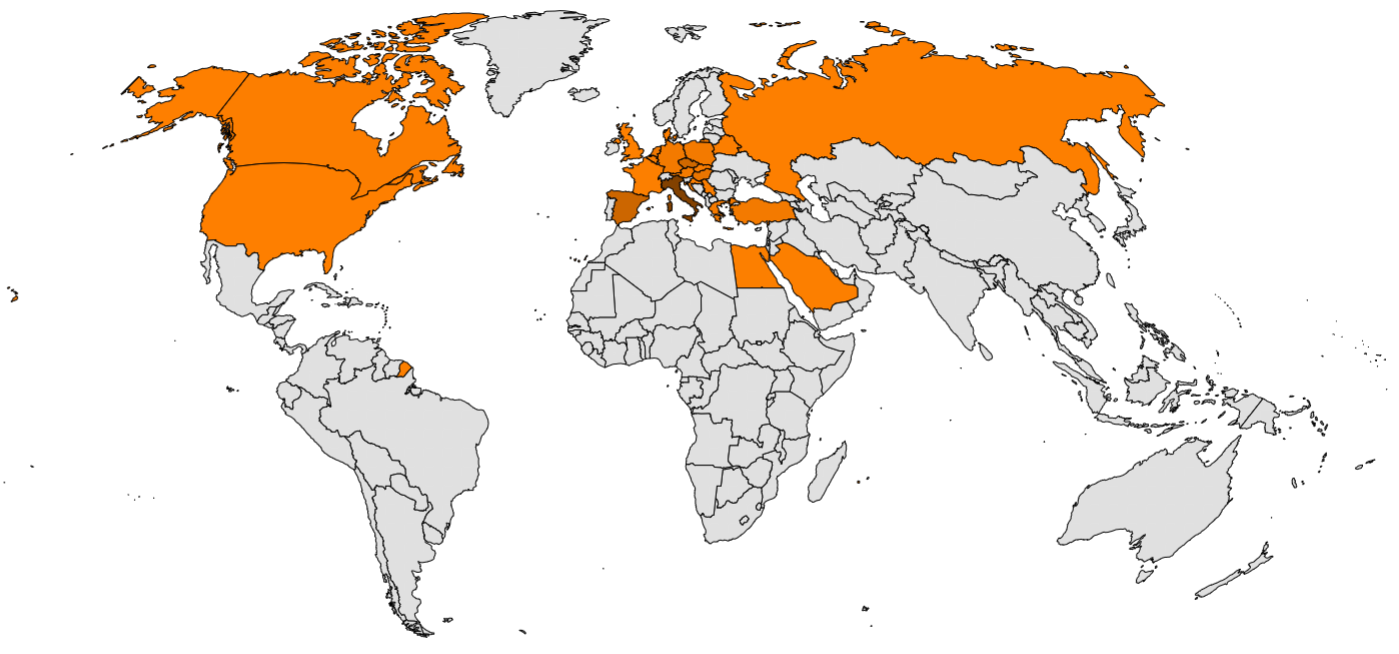
~~

~~Participants were being treated in centres from Italy (n=457, 34.8%), Spain (n=183, 13.9%), Czechia (n=171, 13.0%), Hungary (n=79, 6.0%), Netherlands (n=55, 4.2%), Germany (n=43, 3.3%), Poland and Turkey (n=42, 3.2% each), Austria (n=38, 2.9%), Greece (n=37, 2.8%), Belgium (n=30, 2.3%), Serbia (n=23, 1.8%), Slovakia (n=19, 1.4%), Canada (n=13, 1.0%), Belarus (n=12, 0.9%), Croatia, Egypt, and France (n=11 each, 0.8%), Saudi Arabia (n=10, 0.8%), Russia (n=9, 0.7%), North Macedonia (n=6, 0.5%), United States (n=5, 0.4%), United Kingdom (n=4, 0.3%), and Denmark (n=1, 0.1%).~~

**~~Supplementary figure 2.~~** ~~Baseline underlying condition distribution by pathogen in EPICOVIDEHA-EPIFLUEHA participants with hematological patients diagnosed with respiratory viral infections (September 2023 – March 2024).~~

1. ~~SARS-CoV-2, influenza, RSV, rhinovirus, parainfluenza, and metapneumovirus~~

**~~Supplementary figure 2.~~** ~~Baseline underlying condition distribution by pathogen in EPICOVIDEHA-EPIFLUEHA participants with hematological patients diagnosed with respiratory viral infections (September 2023 – March 2024). (continued)~~

1. ~~Coronavirus non-SARS-CoV-2, enterovirus/rhinovirus, virus combinations, and other virus~~

**~~BMI~~**~~, body mass index,~~ **~~CARV~~**~~, community-acquired respiratory virus;~~ **~~RSV~~**~~, respiratory syncytial virus;~~ **~~SARS-CoV-2~~**~~, severe acute respiratory syndrome coronavirus type 2~~

**~~Supplementary figure 3.~~** ~~30-day mortality distribution by pathogen in EPICOVIDEHA-EPIFLUEHA participants with hematological patients diagnosed with respiratory viral infections (September 2023 – March 2024).~~

**~~CARV~~**~~, community-acquired respiratory virus;~~ **~~RSV~~**~~, respiratory syncytial virus;~~ **~~SARS-CoV-2~~**~~, severe acute respiratory syndrome coronavirus type 2~~
